# Supplementary material for: Ligand coupling mechanism of the human serotonin transporter differentiates substrates from inhibitors
Source: Nat Commun. 2024 Jan 10;15:417. doi: 10.1038/s41467-023-44637-6 (PMC10776687; doi:10.1038/s41467-023-44637-6)
Supplement: Supplementary file 1 — Supplementary Information [file 41467_2023_44637_MOESM1_ESM.pdf]

SUPPLEMENTARY INFORMATION

TITLE

Ligand coupling mechanism of the human serotonin transporter differentiates substrates from inhibitors

AUTHORS

Ralph Gradisch[1], Katharina Schlögl[2], Erika Lazzarin[1], Marco Niello[1,3], Julian Maier[1], Felix P. Mayer[4,5,6], Leticia Alves da Silva[1], Sophie MC Skopec[1], Randy D Blakely[4,5], Harald H Sitte[1,7,8], Marko D Mihovilovic[2], Thomas Stockner[1]*

thomas.stockner@meduniwien.ac.at

AFFILIATIONS

[1] Medical University of Vienna, Institute of Physiology and Pharmacology, Waehringer Straße 13A, 1090 Vienna, Austria

[2] TU Wien, Institute of Applied Synthetic Chemistry, Getreidemarkt 9, 1060 Vienna, Austria

[3] Genetics of Cognition Laboratory, Neuroscience area, Istituto Italiano di Tecnologia, via Morego, 30, 16163 Genova, Italy

[4] Florida Atlantic University, Department of Biomedical Science, Brain Institute, Jupiter, FL 33458, USA

[5] Stiles-Nicholson Brain Institute, Jupiter, FL 33458, USA

[6] Present address: Department of Neuroscience, Faculty of Health and Medical Sciences, University of Copenhagen, DK-2200 Copenhagen, Denmark

[7] Al-Ahliyya Amman University, Hourani Center for Applied Scientific Research, Amman, Jordan

[8] Medical University of Vienna, Center for Addiction Research and Science, Waehringer Straße 13A, 1090 Vienna, Austria

CONTENTS

Supplementary Tables

Supplementary Figures (1-8)

Supplementary Notes

Supplementary Figures (9-12)

Supplementary Methodology

Supplementary Figures (13-19)

Supplementary References

SUPPLEMENTARY TABLES

Supplementary Table 1: Input variables for tICA subspace reduction

	**distance**	**distance**	**angle**
**Extracellular gate closure**	TM1b-TM9up	TM6a-TM9up	TM9up-TM1b-TM6a
**TM6a unwinding**	A331-F335		
**Phenylalanine path**	F334-F335	F334-F548	
**Hydrophobic gate**	I172-Y176	Y176-F335	
**Salt bridge**	R84-E493		

Supplementary Table 2: Calculated kinetic parameters and rate constants

	**IC_{50} (µM)**	**Ratio IC_{50}**	**k_{on} (10^6 M^{-1} s^{-1})**	**k_{off} (s^{-1}) OO**	**k_D (nM) OO**	**Ratio k_D**	**k_{off} (s^{-1}) total**	**k_{off} (s^{-1}) IO**
**M-5HT**	79.35 [69.53, 90.57]	0.06	1.24	3.16 [2.01, 5.34]	2548.39	0.03	0.24 [0.11,?]	2.92
**5HT**	4.49 [3.92, 5.12]	1	6.30	0.55 [0.29, 0.92]	87.05	1	NA	NA
**P-5HT**	0.59 [0.53, 0.68]	7.5	26.06	0.31 [0.22, 0.41]	11.78	7.39	0.04 [0.02, 0.07]	0.27
**B-5HT**	0.27 [0.23, 0.29]	15.0	20.67	0.15 [0.07, 0.24]	7.02	12,4	0.03 [0.02, 0.04]	0.12

OO = outward-open; IO = inward-open. k_{off} IO = k_{off} OO - k_{off} total. Values are represented as mean [95% Confidence Interval]

SUPPLEMENTARY FIGURES

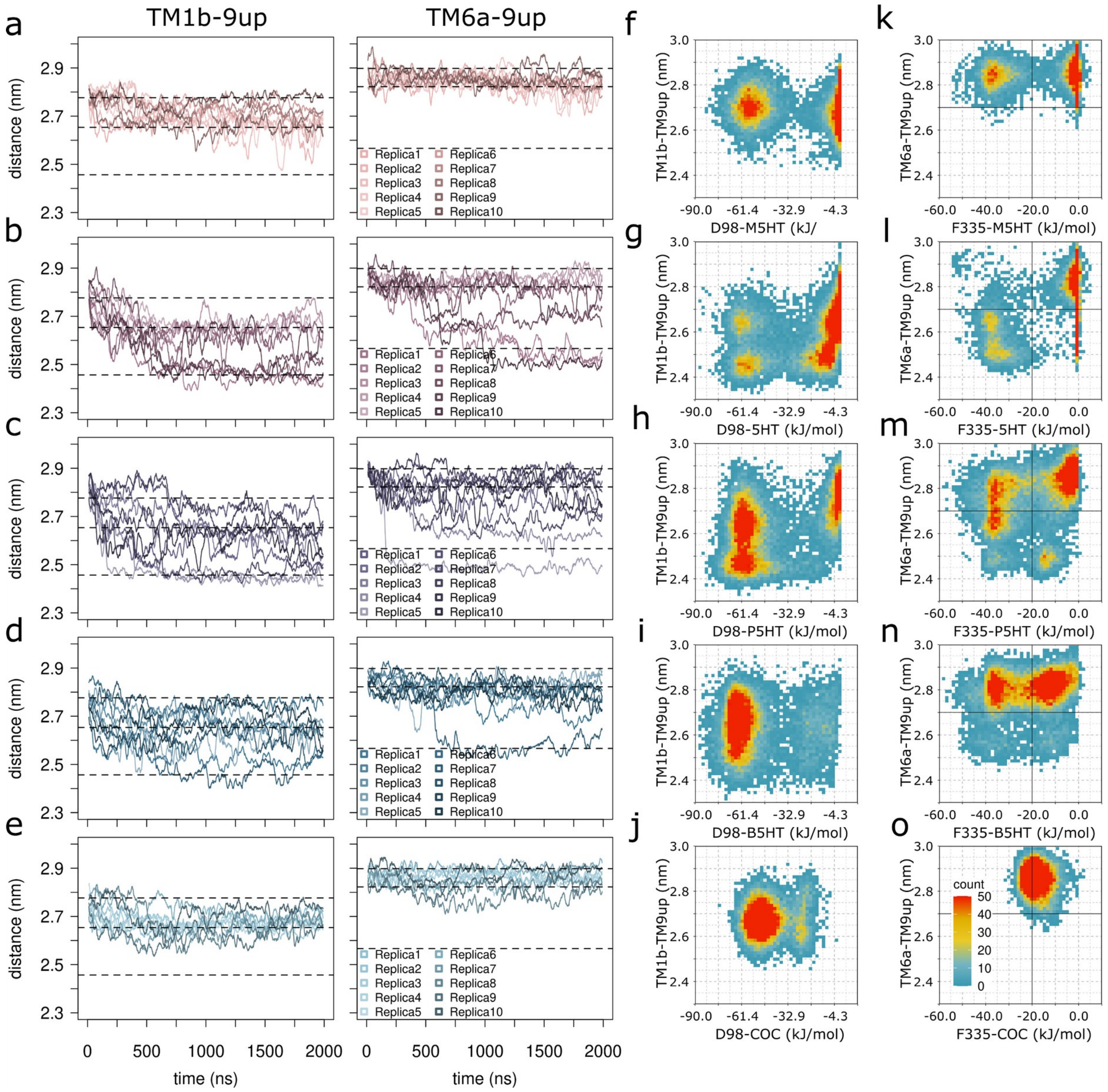

Supplementary Figure 1: Differences in occlusion-behaviour due to modification of the 5HT's alkylamine moiety. *Change of distance (transmembrane helix (TM)1b-TM9up and TM6a-TM9up) over time of all 10 replicas (n=10) of* ***a)*** *methyl (M)-serotonin (5HT),* ***b)*** *5HT,* ***c)*** *propyl (P)-5HT,* ***d)*** *butyl (B)-5HT and* ***e)*** *cocaine (COC). 3D frequency plot of the change in distance (TM1b-TM9up) and the associated change in the total interaction energy between* ***f)*** *M-5HT,* ***g)*** *5HT,* ***h)*** *P-5HT,* ***i)*** *B-5HT,* ***j)*** *COC and the residue D98. 3D frequency plot of the change in distance (TM6a-TM9up) and the associated change in the total interaction energy (kJ/mol) between* ***k)*** *M-5HT,* ***l)*** *5HT,* ***m)*** *P-5HT,* ***n)*** *B-5HT,* ***o)*** *COC and the gating residue F335. The weak and strong interacting conformations are separated by a vertical line. The outward-open and occluded conformations are separated by a horizontal line. M-5HT, 5HT, P-5HT, and B-5HT are colour-coded in peach, mauve, cyber grape, and petrol, respectively.*

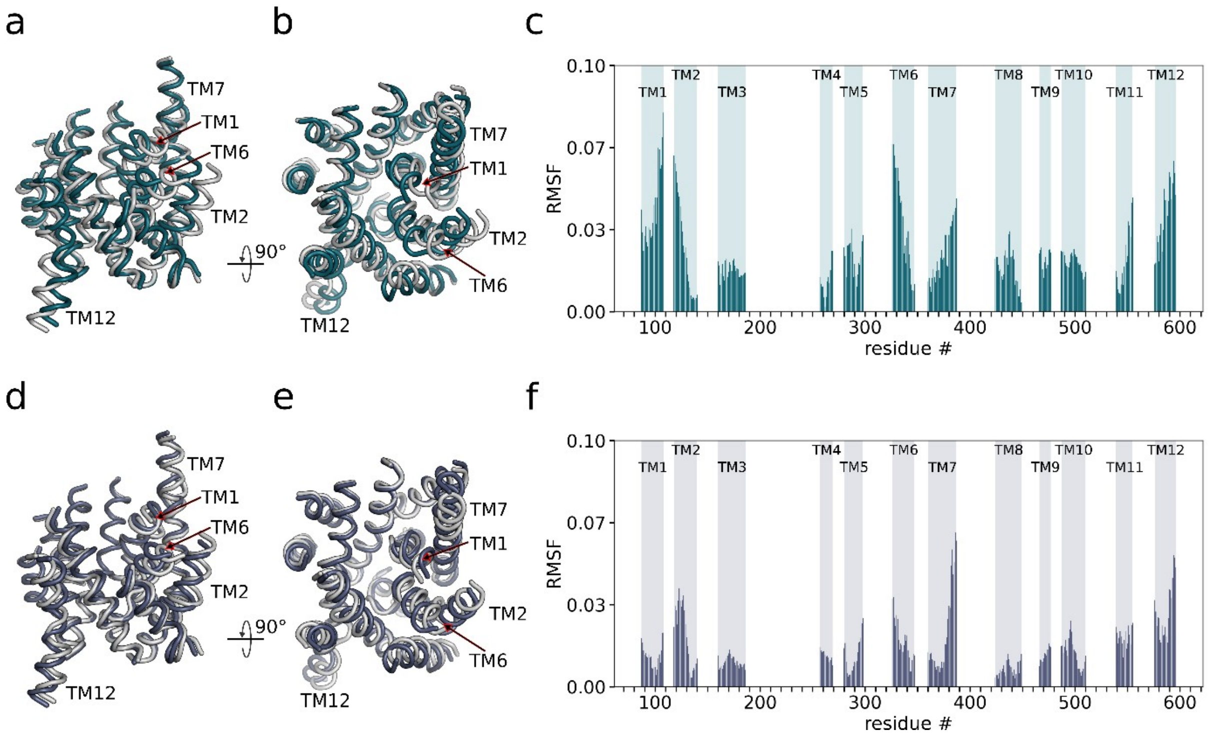

Supplementary Figure 2: Collective motions of hSERT in response to the presence of different compounds. *The principal component analysis was carried out using all 50 trajectories on the* C_{α}*-atoms of all transmembrane helices (TMs) to identify the most extensive conformational changes. The largest motion (principal component 1 (PC1)) is a tilt of TM1, TM2, TM6 and TM7 towards the centre depicted in petrol as* ***a)*** *front view and* ***b)*** *top view. The second largest motion (PC2) represents a rotation mainly of TM2, TM6, TM7 and TM12 highlighted in cyber-grape and shown as* ***d)*** *front view and* ***e)*** *top view. The white structures represent the starting conformation. Root mean square fluctuation (RMSF) per residue of the extreme structures along* ***c)*** *PC1 and* ***f)*** *PC2 are shown.*

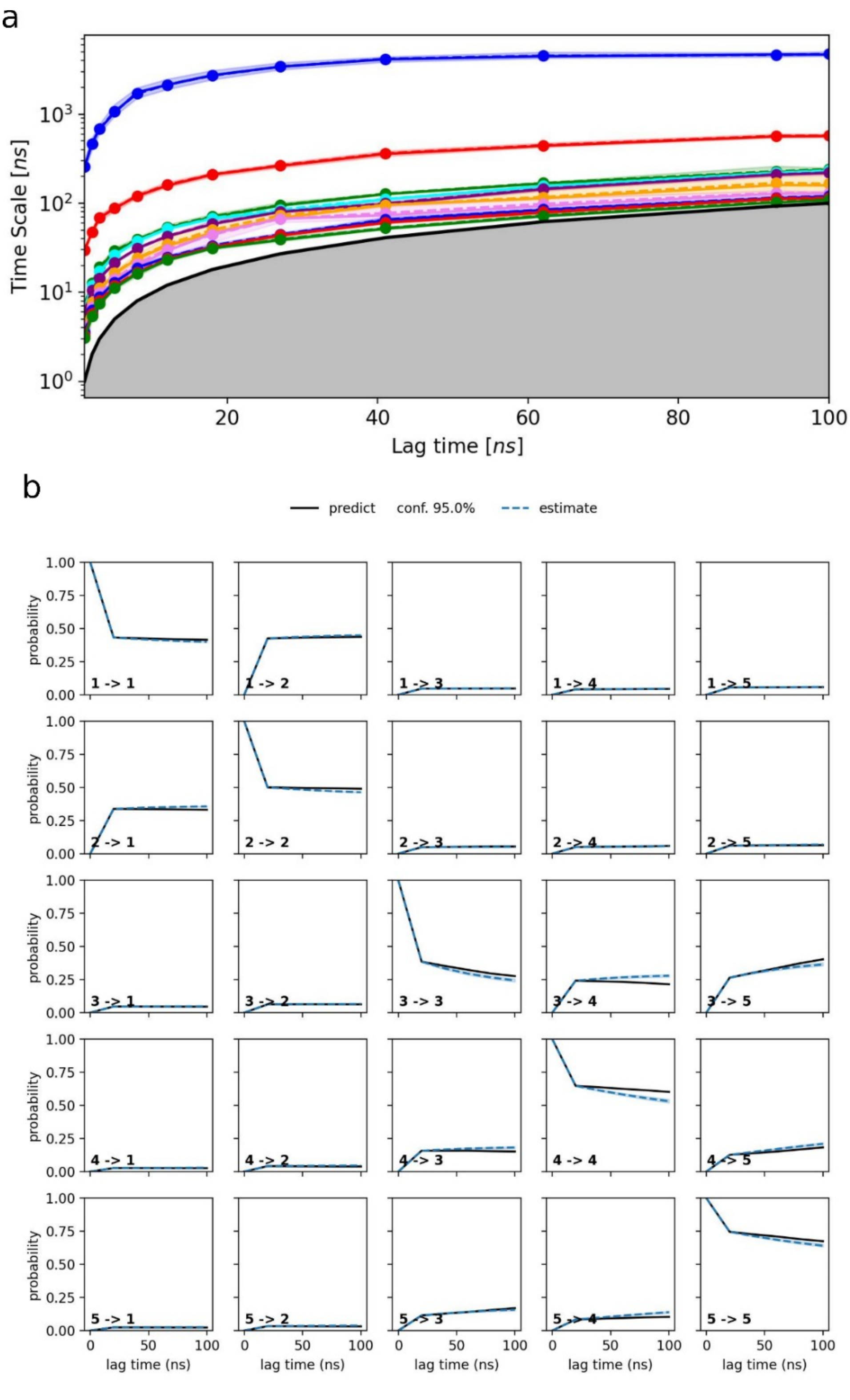

Supplementary Figure 3: Markovian behaviour validation. a) *Time traces are shown for the relation between the lag time and the implied timescales associated with the ten slowest processes, with the blue trace indicating the slowest process. The solid lines refer to the maximum likelihood result, the dashed lines show the ensemble mean computed with a Bayesian sampling procedure. The black line with the grey area indicates the timescale threshold where the MSM cannot resolve processes.* ***b)*** *displays the Chapman-Kolmogorov (CK) test calculated using a lag time of 25 ns and assuming five macrostates. The solid traces show the predicted values obtained from the MSMs, while the dashed*

traces show the estimated values for a longer lag time. The superposition of predicted and estimated values indicates that the MSM assures Markovian behaviour. The non-grey shaded areas in both panels indicate 95% confidence intervals computed using the Bayesian sampling procedure mentioned above.

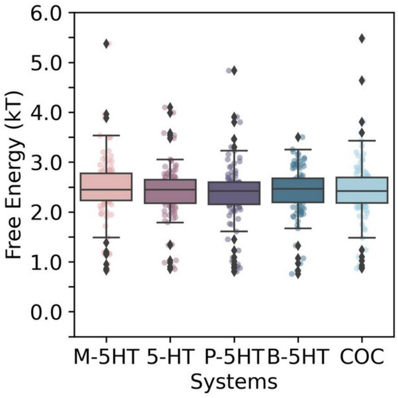

Supplementary Figure 4: Validation of the free energy analysis. *Box plots quantifying the mean value of the average free energy surface of the 2D free energy landscapes are computed by applying the bootstrap methods with replacement. The original population dataset comprising all SERT WT trajectories in complex with compounds was used as input for bootstrapping. The sub-samples originating from this procedure were used first to estimate the MSMs and then the mean value of the free energy surface values of the different sub-populations. A total of 100 sub-populations were generated during bootstrapping to gain 100 mean free energy surface values and estimate the variance. The boxes are defined by the 1st and 3rd quantile, a line within the box representing the median, and two whiskers extending up to 1.5 inter-quartile ranges (IQR) of the lower and upper quartile. Data points plotted as grey squares are supposed to represent outliers as they fall outside the 1.5 IQR range. Conditions are coloured according to the default colour scheme. Methyl (M)-serotonin (5HT), 5HT, propyl (P)-5HT, butyl (B)-5HT, and cocaine (COC) are colour-coded in peach, mauve, cyber grape, petrol, and light blue, respectively.*

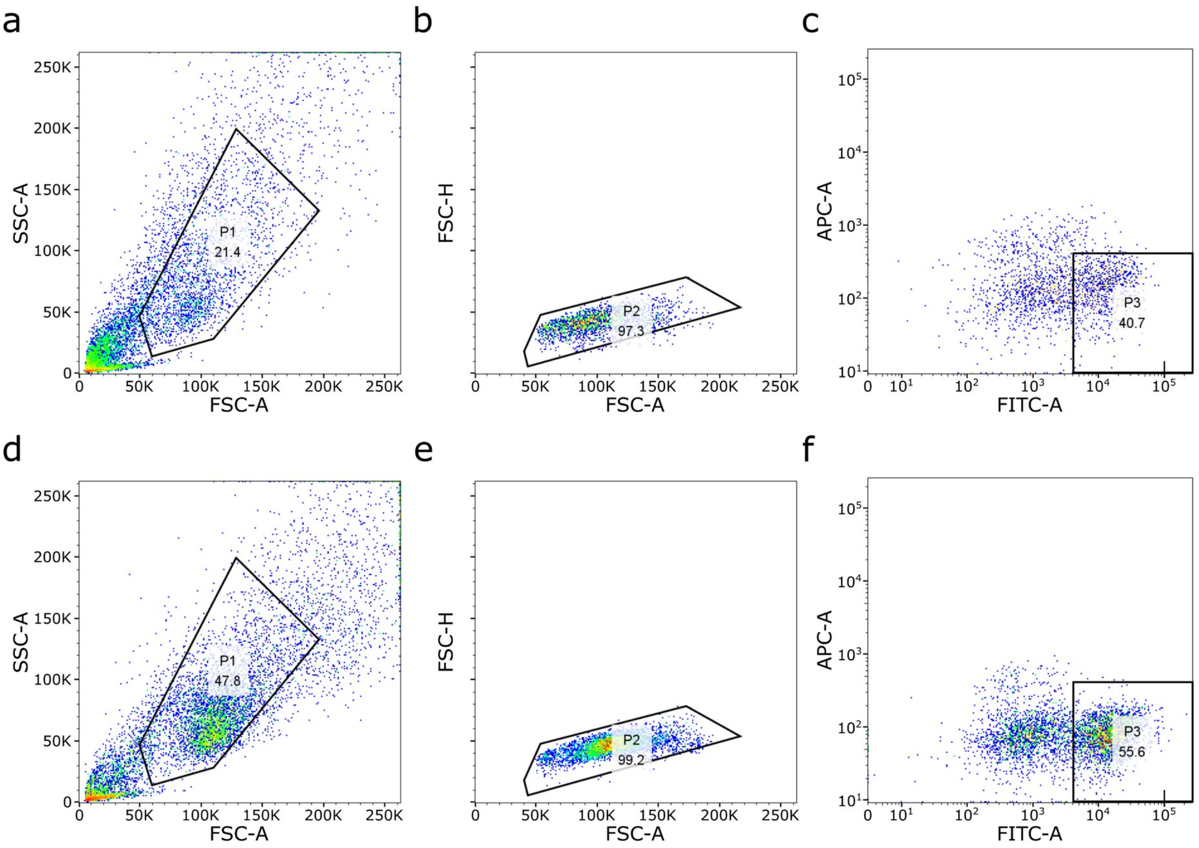

Supplementary Figure 5: FACS sorting strategy. *Flow cytometry density scatter plots representing the sorting strategy of the **a-c)** single poly sorted (SPS) and **d-f)** double poly sorted (DPS) N-terminally fused hSERT (YhSERT) wild type cell lines. Population (P) 3 originated from P2, which was derived from P1. Abbreviations: Side Scatter (SSC)-Area (A), Forward Scatter (FSC)-Height (H), Fluorescein isothiocyanate (FITC), and Allophycocyanin (APC).*

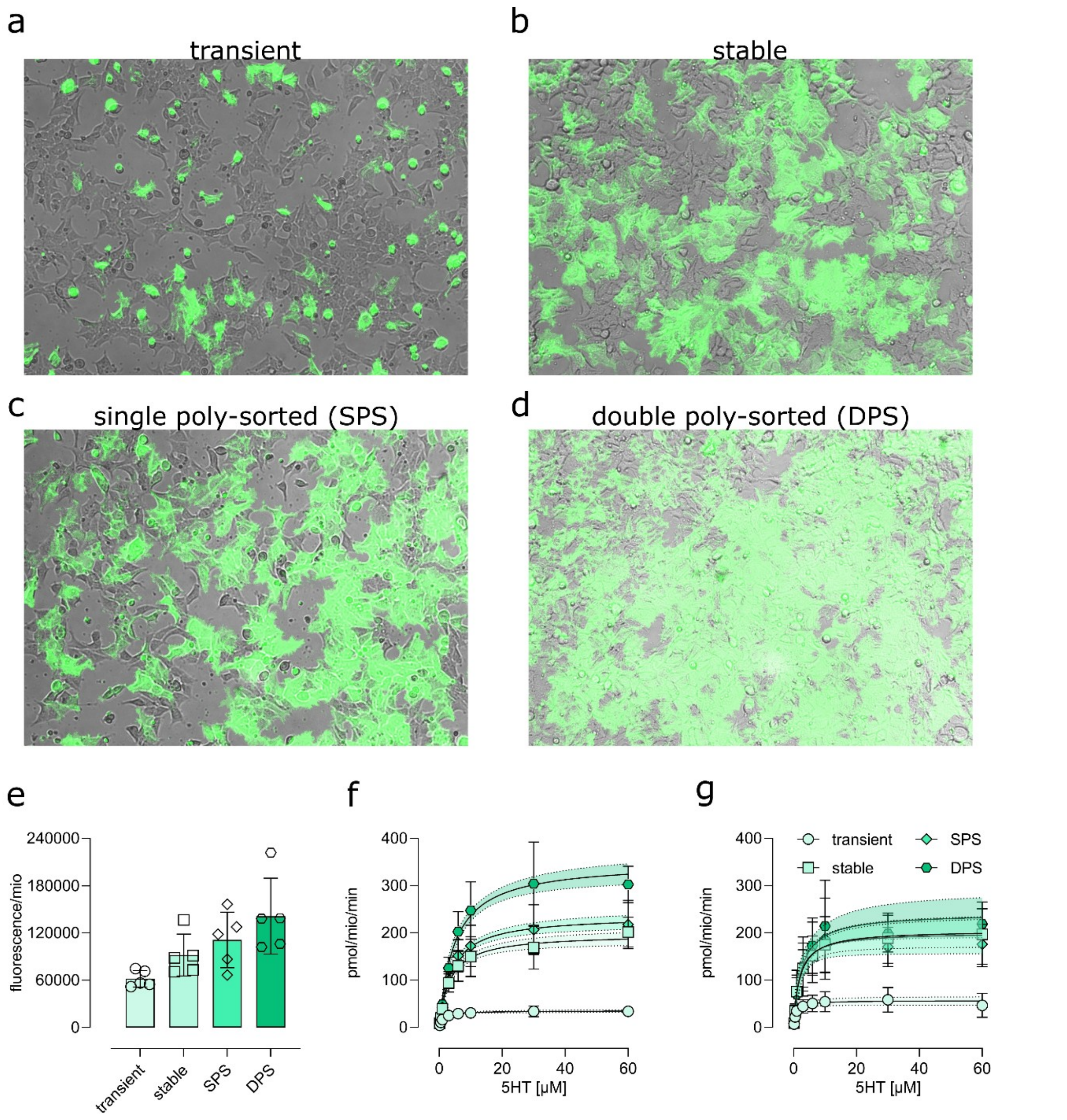

Supplementary Figure 6: Establishing the double poly-sorted (DPS) YhSERT expressing HEK cell line. *Expression level of N-terminally fused eYFP-hSERT (YhSERT) in HEK cells **a)** 24 hours after transfection, **b)** after maintaining stable expression, **c)** after the first round of fluorescence-activated cell sorting (FACS) and **d)** after the second round of FACS. **e)** Increase of total fluorescence associated with an increase of expression of YhSERT. Data are represented as mean ± SD from n=5 independent dishes used to conduct the experiments shown in f. **f)** Dose-dependent SERT-mediated [3H]-serotonin (5HT) uptake within 1 minute of the 4 different cell lines. Data are shown as mean ± SD from n=5 independent experiments measured in triplicates. **g)** Plot f normalised to the total fluorescence shown in e. Confidence intervals (95 %) of the curve fittings are plotted within ± SD and delimited by dashed lines.*

The dose dependence of the maximal rate of inhibition (K_{app}) allows for detecting if a compound stabilises SERT in only one conformation of the transport cycle, such as cocaine or in multiple states, like ibogaine. The K_{app} reaches a stable plateau if only the one state is stabilised, while it does not saturate if it binds multiple states of the transport cycle, e.g. ibogaine. 5HT and its derivatives, as well as cocaine, saturate at a K_{app} of approximately 2 s^{-1}, consistent with a binding mode requiring only one state.

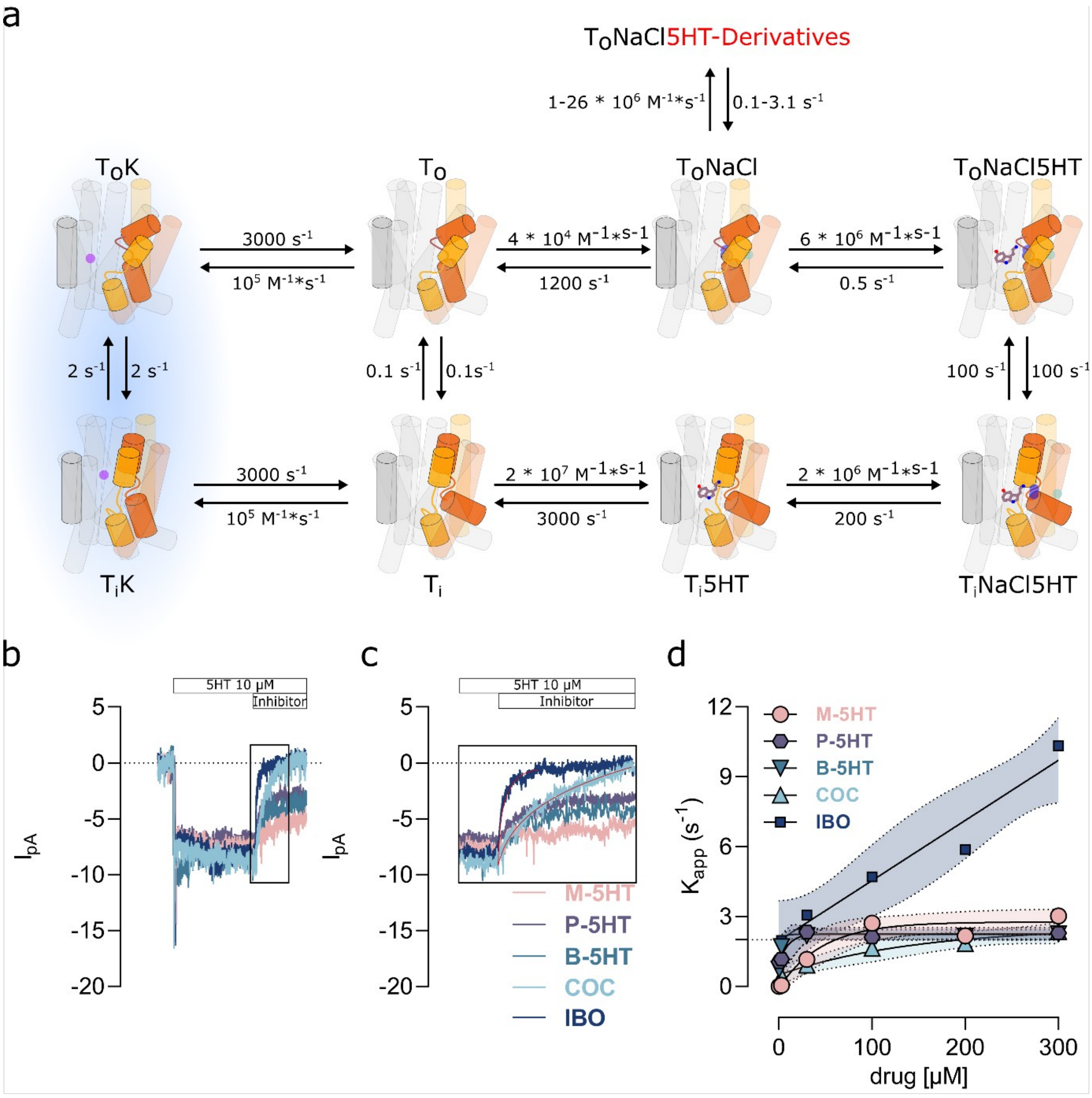

Supplementary Figure 7: Conformational state dependence and its impact on drug binding kinetics. ***a)*** *Simplified kinetic model of SERT-mediated 5HT reuptake and inhibitor binding adapted from Schicker et al.*[1] *and Niello et al.*[2]*. The kinetic model displays main and intermediate states of SERT within the transport cycle as described by the alternating access model. Arrows and numbers display the kinetics between the states. The scaffold domain is kept in grey. Transmembrane helix 1 (TM1), TM2 are coloured in red and TM6, TM7 are highlighted in yellow. The blurred and light blue ellipse highlights the slowest (time-limiting) step of the transport cycle. Serotonin (5HT), Na^+, K^+, and Cl^- are coloured in mauve, blue, purple, and cyan, respectively. Abbreviations: T_o: transporter outward-open,*

T_i: transporter inward-open. ***b)*** *Representative trace of the binding kinetics of 5HT-mediated current inhibition (5HT concertation: 10 µM) by saturating concentrations of M-5HT (200 µM), P-5HT (200 µM), B-5HT (200 µM), cocaine (200 µM) and ibogaine (200 µM).* ***c)*** *Close-up of the kinetics of inhibitor binding shown in b. Mono-exponential curves fitted into the current inhibition by COC and IBO are shown in red.* ***d)*** *Current inhibition rates (s^{-1}) of cocaine (COC), ibogaine (IBO) and the 5HT-derivatives (methyl (M)-5HT, propyl (P)-5HT, and butyl (B)-5HT) as a function of the compound concentration. COC and all 5HT-derivatives saturate around the rate-limiting step at 2 s^{-1}. In contrast, IBO shows a linear dose-dependency, which implies that its binding is not dependent on binding the outward-open conformation[3]. Data are shown as the mean of n=5 (M-5HT), n=8 (P-5HT), n=5 (B-5HT), n=9 (COC), and n=7 (IBO) recordings of independent cells. M-5HT, P-5HT, B-5HT, COC, and IBO are colour-coded in peach, cyber grape, petrol, light blue, and dark blue, respectively. Confidence intervals (95 %) of the curve fittings are plotted within ± SD and delimited by dashed lines.*

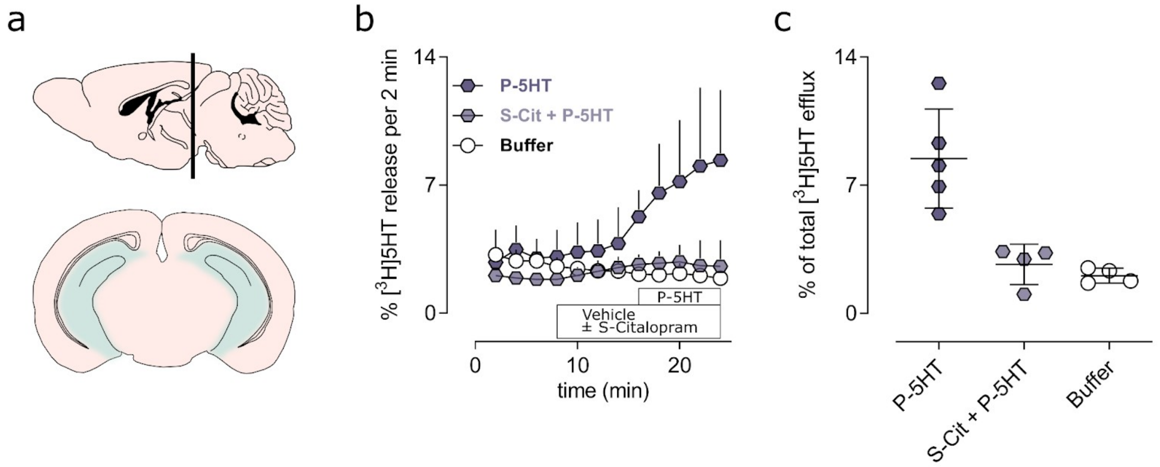

Supplementary Figure 8: Ex-vivo release properties of P-5HT. a) *top) Cartoon of a sagittal section of a mouse brain. bottom) Cartoon of a coronal section highlighting in light-petrol the hippocampal tissue used for experiments (Anterior/Posterior: -3.00 mm relative to bregma). As for the in-vitro release assay, the tissue punches were loaded with 0.4 µM [3H]5HT.* ***b)*** *Averaged traces of n=5 (propyl (P)-5HT) and n=4 (escitalopram (S-Cit) + P-5HT and buffer) independent experiment. Data are given as the mean + SD. After collecting three basal efflux fractions, four fractions a' 2 min were collected where either S-Cit or vehicle was added to the superfusion system. At t=14 min, the brain punches were challenged with P-5HT (10 µM) in the presence or absence of S-Cit (10 µM) or vehicle for a total of five 2-minute fractions.* ***c)*** *Percentage of hSERT-mediated total efflux. Plotted is the average total efflux of the last four fractions shown in b) of each independent experiment. Data is shown as the mean ± SD. P-5HT, S-Cit + P-5HT, and Buffer are coloured cyber grape, light cyber grape and white, respectively.*

SUPPLEMENTARY NOTES:

Simulations of hSERT D98E

MD simulations show that the D98E mutant alters the balance of key interactions needed for substrate transport

The single point mutation (D98E) was introduced in each of the 50 starting structures of SERT WT apo and in complex with M-5HT, 5HT, P-5HT, and B-5HT by using the Wizard Mutagenesis tool provided by PyMOL. All atom simulations and analyses were performed in accordance with the procedure described in the Material and Methods section. Each system reached a simulation length of 1 µs, hence a total simulation length of 50 µs was computed for the hSERT D98E mutant.

The D98E mutant alters the conformational equilibrium of apo SERT

A comparison between SERT wildtype (WT) and the D98E mutant in the apo state shows that the interactions of residue 98 with residues and ions in close proximity differ between aspartate (D98) and glutamate (E98). The key interacting residues are shown in SI Fig. 9f,g. Binding and stabilisation of Na1 differ as D98 shows one specific interaction with Na1 that completes the interaction shell of Na1 **(SI Fig. 9a)**, while the D98E mutant shows multiple conformations, suggesting that the D98E SERT variant has difficulties to maintain the same interaction with Na1. Consistently, Na1 is not as stably bound **(SI Fig. 9b)** and can reposition to the region, which overlaps with the region in the S1 that the amino group of the substrate occupies in SERT WT. The carboxylate group of residue 98 (bundle domain) shows one consistent distance to the hydroxyl group of Y176 in SERT WT **(SI Fig. 9c)**, but two distances for the D98E mutant. The sidechain of D98E is sufficiently long to directly interact with R104, which is part of the outer gate bridge **(SI Fig. 9d)**. These local changes alter the global conformation of SERT. We find one broad distribution of distances between TM1b (bundle domain) and TM9up (scaffold domain), while in the D98E mutant, the distribution is changed to a bimodal distribution with the outer vestibule further open or more closed.

These data show that the local change of the interaction pattern of residue 98 in the S1 leads to global conformational changes that alter the accessibility to the S1 through the outer vestibule.

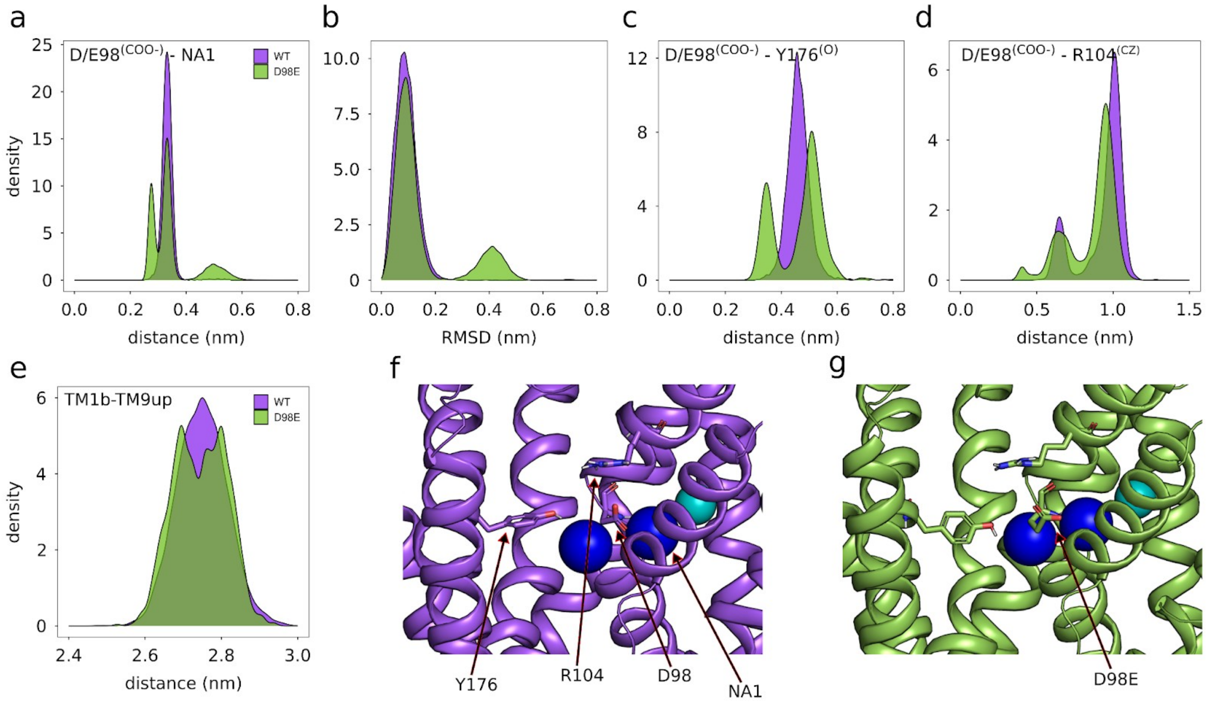

Supplementary Figure 9*: Conformational consequences of the D98E mutation in the apo state**. *Distance distribution of the apo state of the 10 SERT WT and the 10 SERT D98E mutant trajectories* ***a) *measured between the carboxylate (COO-) of residue 98 (aspartate or glutamate (D/E)) and Na1.* ***b)*** *Histogram of RMSD values of Na1 measured with respect to sodium bound to the NA1 site.* ***c)*** *Distribution of distances between the carboxylate (COO-) of residue 98 (aspartate or glutamate (D/E)), respectively and the hydroxyl oxygen (O) of Y176.* ***d)*** *Distance distribution between the carboxylate (COO-) of residue D/E98 and atom CZ of R104. WT is coloured in purple, while D98E is coloured in green.* ***e)*** *Distance distribution measured between the Cα atoms of TM1b (residue 99-111) and TM9up (residue 475-477). Front view of the S1 of* ***f)*** *WT and* ***g)*** *the D98E mutant displayed as cartoons. Residues are represented as sticks and coloured according to atom type. The two sodium and chloride are visualised as spheres and coloured in blue and cyan, respectively.*

The D98E mutant hampers the substrate-triggered occlusion

A comparison of simulations of WT SERT with simulations of the D98E mutant showed that SERT occlusion is altered by the D98E mutation. For M-5HT, the increased interaction between the glutamate in position 98 with the positively charged nitrogen of the compound **(SI Fig. 10a)**, indicated by a smaller distance, leads only to a small repositioning of TM1b **(SI Fig. 10c)**. At the same time the longer glutamate hinders the formation of a continuous interaction between the positively charged nitrogen of M-5HT and the helical dipole of TM6a, specifically with the backbone carbonyl oxygen of F335 **(SI Fig. 10b)**. This lack of interaction prevents occlusion of SERT **(SI Fig. 10d)**. For 5HT, the longer sidechain of the glutamate in position 98 **(SI Fig. 10a)** can reach to the substrate without the need of a partial closing motions of TM1b **(SI Fig. 10c)**. In parallel, the interaction with the carbonyl oxygen of F335 is hampered **(SI Fig. 10b)**, hence the occlusion motion of TM6a was prevented **(SI Fig. 10d)**, resulting in an inhibition of the occlusion of the D98E variant of SERT in presence of 5HT. For P-5HT and B-5HT, the longer sidechain of the glutamate in position 98 opposes occlusion in addition to the already too long aliphatic chain of the two substrates.

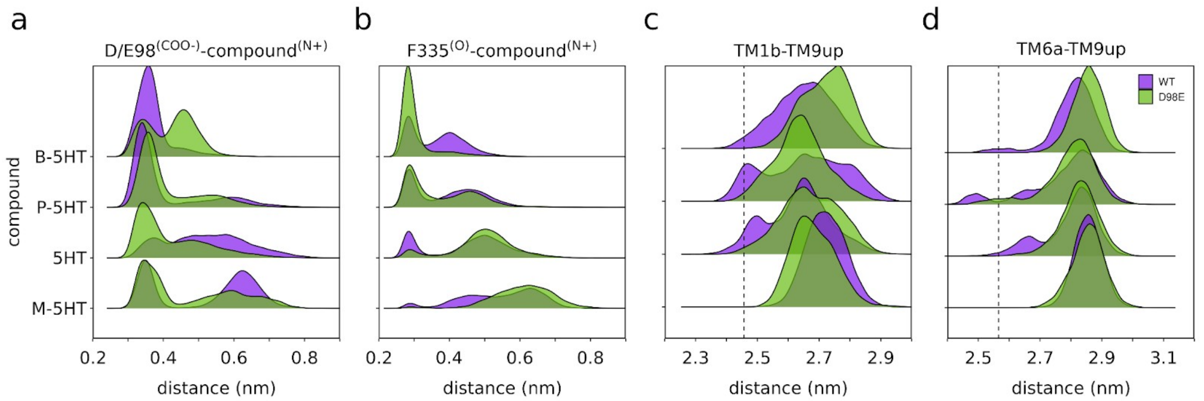

Supplementary Figure 10: D98E and its impact on substrate interaction and occlusion. *Distances of the 40 SERT WT and the 40 SERT D98E mutant trajectories in complex with the 5HT homologous series, displayed as histograms of **a)** distance between the carboxylate (COO-) of residue 98 (aspartate or glutamate (D/E)) and the tertiary amino group (N+) of the four tested compounds, **b)** distance between the backbone carbonyl oxygen (O) of F335 and the charged amino group (N+) of the four tested compounds, **c)** distance between the Cα atoms of TM1b and TM9up, and **d)** between the Cα atoms of TM6a and TM9up, coloured in purple and green, respectively. The vertical dashed lines indicate the distances between TM1b-TM9up and TM6a-TM9up as measured in the cryo-EM structure of hSERT in the inward-open conformation (PDB ID: 6DZZ).*

The direction of motions of the bundle domain is unchanged, but the amplitude is reduced

Projection of the trajectories of the D98E mutant of apo SERT onto the two main principal components (PC) as observed for SERT WT **(Fig. 2 and SI Fig. 2)** revealed the same type of motions **(SI Fig. 11a-f)**. Motions of PC1 describe the main motion of occlusion **(SI Fig. 11a,b)**, while PC2 represents a rotation of the bundle domain perpendicular to the motion of occlusion **(SI Fig. 11d,e)**. The per residue pattern of motion amplitudes of SERT WT **(SI Fig. 2)** and the D98E mutant **(SI Fig. 11c,f)** along PC1 and PC2 are the same. The amplitudes of motions **(SI Fig. 11c,f)** of individual residues of the bundle domain were smaller for PC1, while for PC2 also the amplitudes of motions are consistent between WT SERT and the D98E mutant. The smaller amplitudes of the bundle domain motions are consistent with the measures of distances across the outer vestibule between TM1b-TM9up and TM6a-TM9up and shows that the D98E mutation did not change the mechanics of the closure motions but altered its amplitude.

The 2D projections **(SI Fig. 11g)** of all trajectories of the D98E mutant (SERT apo and in complex with the M-5HT, 5HT, P-5HT and B-5HT) show that the mutation inhibits occlusion as compared to WT SERT **(Fig. 2)**. While M-5HT and B-5HT do not induce occlusion, 5HT and P-5HT have a smaller propensity to induce SERT occlusion.

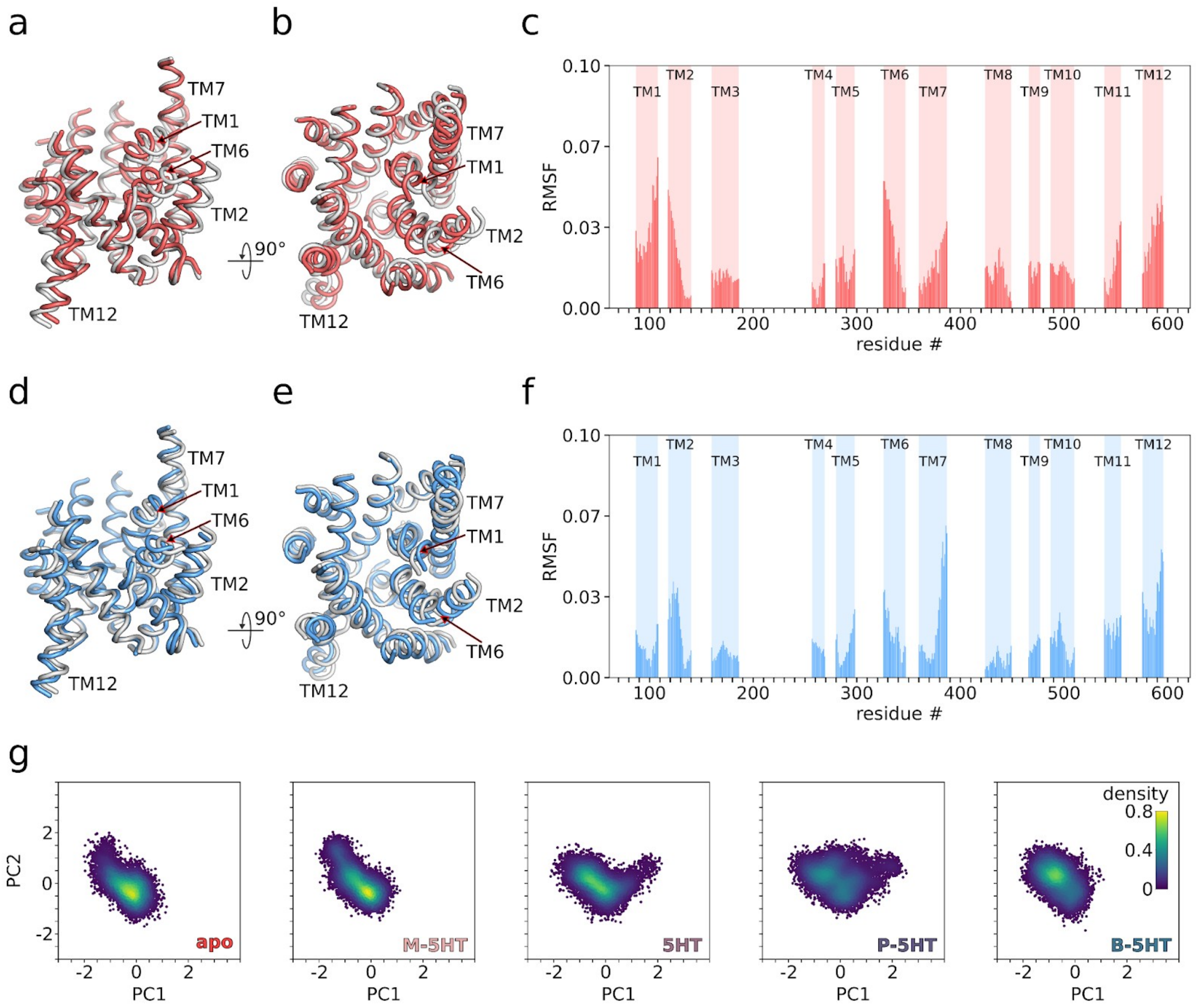

Supplementary Figure 11: Principal component analysis of hSERT D98E apo and in complex with 5HT and its derivatives. *The principal component analysis (PCA) was carried out using all 50 trajectories of the D98E mutant of SERT on the Cα-atoms of all transmembrane helices (TMs) and projected onto the eigenvectors derived for the WT simulations as shown in Fig. 2 and SI Fig. 2 to allow for direct comparison. Front view of the two extreme structures of* ***a)*** *Principal component 1 (PC1) and* ***d)*** *PC2. Top view of the extreme structures of* ***b)*** *PC1 and* ***e)*** *PC2. Structures in white represent the starting structures, whereas the coloured structures (red and blue) depict the conformations with the largest changes. The associated per residue root mean square fluctuation (RMSF) of motions along* ***c)*** *PC1 and* ***f)*** *PC2, coloured in red and blue, respectively.* ***g)*** *2D projections of all 10 (n=10) simulations of the SERT D98E mutant (apo and in complex with methyl (M)-5HT, 5HT, propyl (P)-5HT, and butyl (B)-5HT) along PC1 and PC2 that describe the largest motions of SERT WT. Apo, M-5HT, 5HT, P-5HT, and B-5HT are colour-coded in red, peach, mauve, cyber grape, and petrol, respectively.*

The D98E mutant alters the conformations of TM1b and TM6a and opening of the outer vestibule

The distances between TM1b (residue 99-111) and TM9up (residue 475-477) as well as between TM6a (residue 328-338) and TM9up are a surrogate measure of the closure of the outer vestibule and of bundle domain motions **(SI Fig. 12)**. TM9 is part of the scaffold domain, while TM1 and TM6 are part of the bundle domain. These helices reside on

opposite sides of the vestibule. Consistent with the above measures, the time traces show a changed open geometry of the apo D98E mutant and altered ligand-induced closing dynamics.

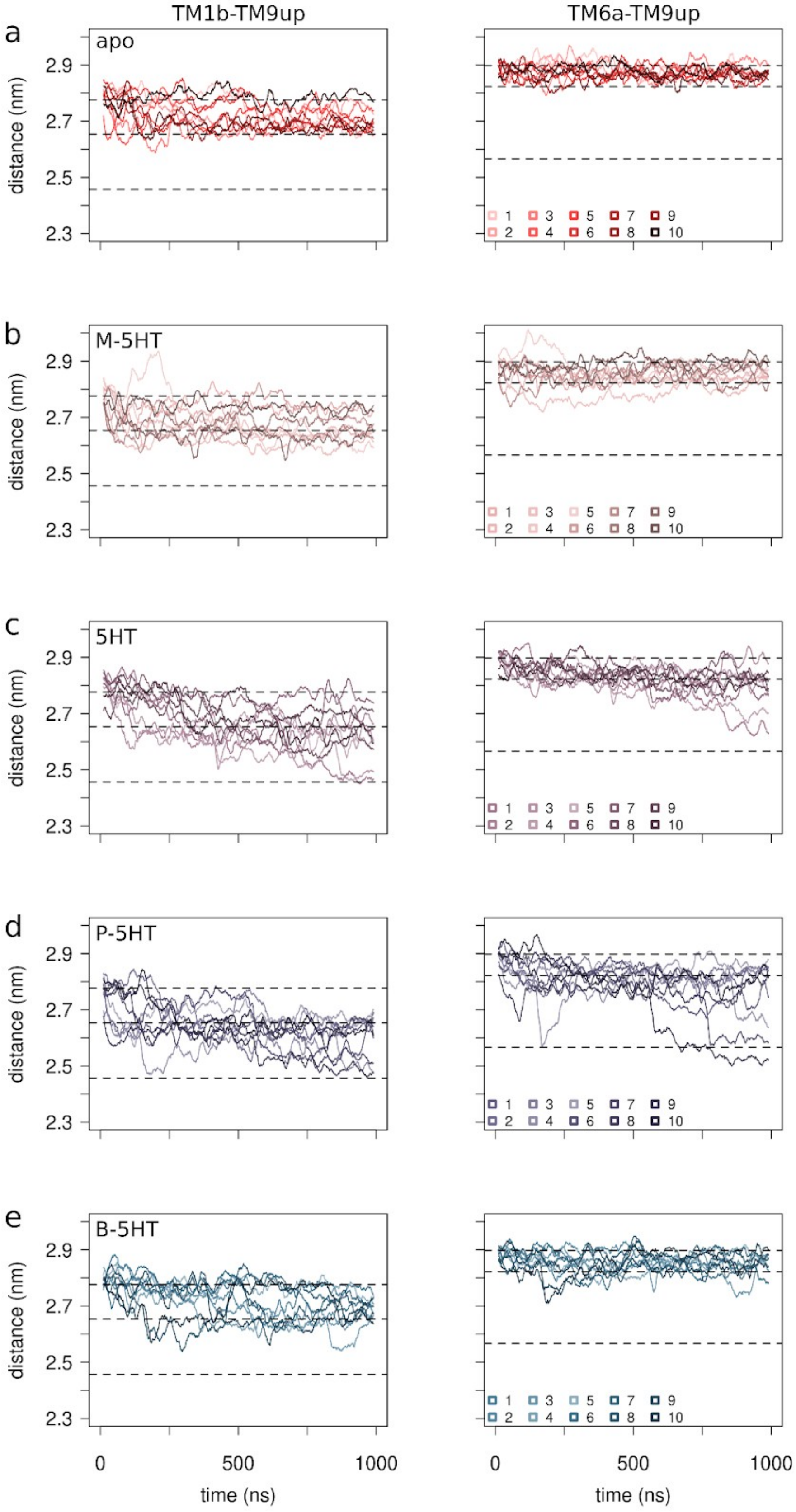

Supplementary Figure 12: Distance measurements of the apo hSERT D98E mutant and in complex with 5HT and its derivatives. *Change of distances (nm) of left) transmembrane helix (TM)1b-TM9up and right) TM6a-TM9up over time (ns) of all 10 replicas (n=10) of* ***a)*** *apo,* ***b)*** *methyl (M)-5HT,* ***c)*** *5HT,* ***d)*** *propyl (P)-5HT, and* ***e)*** *butyl (B)-5HT. Apo, M-5HT, 5HT, P-5HT, and B-5HT are colour-coded in red, peach, mauve, cyber grape, and petrol, respectively.*

SUPPLEMENTARY METHODOLOGY

Synthesis of 5HT analogues with modified alkyl chain length

Analysis of the simulation data generated the testable hypothesis that a modification of the length of the alkyl chain of 5HT should change transport properties of SERT. To experimentally verify this prediction, we developed a synthetic strategy for the compounds M-5HT, P-5HT and B-5HT, as these compounds were rarely investigated[4,5] and no feasible syntheses were described.

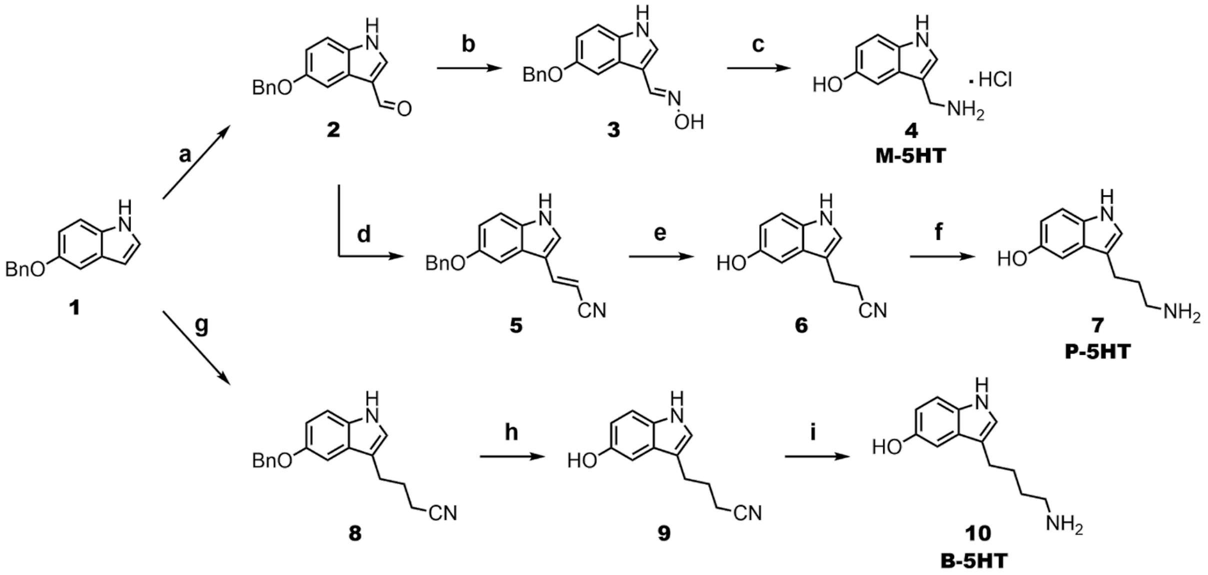

***Supplementary Figure 13: Chemical synthesis of 5HT analogues**. **Synthetic pathway towards M-5HT, P-5HT and B-5HT**. Conditions: **a)** $POCl_3$, AcOH, DMF, 55°C, **b)** NH_2OH•HCl, MeOH, rt, **c)** 10% Pd/C, conc. HCl, H_2, MeOH, rt, **d)** methyltriphenylphosphonium chloride, DBU, toluene, 115°C, **e)** 10% Pd/C, H_2, MeOH, 55°C, **f)** $LiAlH_4$, THF, 57°C, **g)** MeMgI, toluene, rt, then 4-brombutyronitrile, 70°C, **h)** 10% Pd/C, H_2, MeOH, 40°C, **i)** $LiAlH_4$, THF, 55°C.*

The steps of the chemical syntheses are outlined in SI Fig 13. As starting material, 5-(benzyloxy)-1H-indole (**1**) was selected, as protecting the 5-hydroxy group was necessary for easier synthetic manipulation and the benzyl group additionally allowed for reductive cleavage, conveniently fitting later synthetic steps. In the first step, an aldehyde moiety was introduced by a regioselective Vilsmeyer-Haack reaction[6] yielding compound **2**. Noteworthy, adding catalytic amounts of acetic acid accelerated the reaction considerably by facilitating acid-catalysed hydrolysis of the imine-intermediate. The obtained aldehyde **2** was interconverted to oxime **3** using hydroxylamine hydrochloride[6]. Deprotection of the 5-hydroxy group and the reduction of the oxime to the amine could be performed simultaneously to reach the hydrochloride salt of M-5HT (**4**) in 87% yield over 3 steps[7].

For the synthesis of P-5HT (**7**), first, a Wittig reaction[6] was performed on compound **2**, simultaneously elongating the carbon chain and introducing a nitrile group yielding compound **5**. Next, we had planned to reduce the alkene and the nitrile group while also

cleaving the benzyl group by choosing the correct reducing conditions. However, this was not possible as even under harsh conditions the nitrile group would not be reduced. Hence, a sequential approach was employed. First, compound **6** was obtained *via* reductive hydrogenation that led to deprotection of the 5-hydroxy group and reduction of the double bond[8]. P-5HT (**7**) was reached through reduction of the nitrile of compound **6** using $LiAlH_4$[9,10]. The yield of the synthesis of P-5HT (**7**) was 71% over three steps. The hydrochloride salt of P-5HT (**7**) could not be obtained as exposure to conc. HCl in dioxane led to decomposition of the material. However, NMR-based stability studies **(SI Fig. 18, 18a-e)** confirmed high chemical stability of the pure P-5HT as free amine base, as no degradation in solution (DMSO) could be observed even after storage up to five weeks at room temperature.

The synthesis of B-5HT (**10**) required a different synthetic route as analogous Wittig reactions with compound **2** failed, most likely due to lacking conjugation of the yield to the electron withdrawing nitrile group. Starting from compound **1,** compound **8** could be synthesised in 28% yield using Grignard reagent MeMgI as base and a suitable CN-bearing alkyl bromide as reagent[11]. Next, a similar synthetic cascade as performed for compound P-5HT (**7**) led to the isolation of B-5HT (**10**) with an overall yield of 15% over three steps[8–10]. The stability of B-5HT was comparable to P-5HT as confirmed by NMR stability studies **(SI Fig. 19, 19a,b)**.

5-(Benzyloxy)-1*H*-indole-3-carbaldehyde (2)

Compound **2** was prepared according to a modified literature procedure[6]. 5-(Benzyloxy)-1*H*-indole **1** (2.52 g, 11.28 mmol, 1.0 equiv.) was dissolved in dry DMF (22 mL) under argon. The mixture was cooled to 0°C. $POCl_3$ (1.55 mL, 16.58 mmol, 1.5 equiv.) was added in portions followed by acetic acid (0.1 mL). The reaction was conducted at 55°C overnight. After TLC monitoring (1:1 LP:EtOAc) confirmed full conversion, the reaction mixture was poured into ice water (150 mL) and 2 N NaOH (50 mL) was added. The product was filtered off as yellow precipitate, washed with water twice and dried *in vacuo*. The material was obtained as yellow solid (2.53 g, 90%) and used crude (purity ≥ 90%) for the next step.
mp: 240.2-244.7 °C, ^{1}H NMR (400 MHz, DMSO-d_6) δ = 5.12 (s, 2H), 6.97 (dd, *J* = 2.5, 8.8 Hz, 1H), 7.29 – 7.35 (m, 1H), 7.36 – 7.44 (m, 3H), 7.46 – 7.50 (m, 2H), 7.69 (d, *J* = 2.5 Hz, 1H), 8.21 (s, 1H), 9.88 (s, 1H) ppm, **(SI Fig. 14)**. ^{13}C NMR (101 MHz, DMSO-d_6) δ = 69.7, 104.1, 113.3, 113.9, 118.1, 124.9, 127.6 (2C), 127.8, 128.4 (2C), 132.1, 137.5, 138.6, 154.7, 184.9 ppm, **(SI Fig. 14)**.

(*E*)-5-(Benzyloxy)-1*H*-indole-3-carbaldehyde oxime (3)

Compound **3** was prepared according to a modified literature procedure[6]. Starting material **2** (403.5 mg, 1.61 mmol, 1.0 equiv.) and hydroxylamine hydrochloride (137.2 mg, 1.97 mmol, 1.2 equiv.) were dissolved in MeOH (16 mL) and stirred at rt overnight. After TLC monitoring (1:2 LP:EtOAc) confirmed full conversion, water (20 mL) was added to the reaction mixture whereupon the product crashed out as pink solid. The material was filtered

off, washed with water, and dried under *vacuo*. The material was obtained as pink solid (432.0 mg, quant.) and used crude (purity ≥ 90%) for the next step.
mp: 174.2-175.3 °C, ^{1}H NMR (400 MHz, DMSO-d_6) δ = 5.13 (s, 2H), 6.87 (dd, *J* = 2.4, 8.8 Hz, 1H), 7.33 (d, *J* = 8.5 Hz, 2H), 7.36 – 7.43 (m, 2H), 7.46 – 7.53 (m, 3H), 7.75 (s, 1H), 8.16 (d, *J* = 2.8 Hz, 1H), 11.09 (s, 1H), 11.43 (d, *J* = 2.9 Hz, 1H) ppm, **(SI Fig. 14)**. ^{13}C NMR (101 MHz, DMSO-d_6) δ = 69.7, 101.8, 106.3, 112.4 (2C), 126.8, 127.7, 127.8 (2C), 128.4 (2C), 130.1, 131.0, 137.7, 138.5, 153.2 ppm, **(SI Fig. 14)**. HRMS calcd. $[C_{16}H_{14}N_2O_2\text{-}H]^-$: 265.0983; found: 265.0980.

3-(Aminomethyl)-1*H*-indol-5-ol hydrochloride (4)

Compound **4** was prepared according to a modified literature procedure[7]. To starting material **3** (342.9 mg, 1.29 mmol, 1.0 equiv.) and 10% Pd/C (35.1 mg, 5w%), EtOH (11.5 mL) was added. Next, conc. HCl (1 mL) was added. Hydrogen atmosphere of 1 bar was established and the reaction was stirred at rt for 3.5 hours until TLC monitoring (1:1 LP:EtOAc) showed full consumption of the starting material. The reaction mixture was filtered over a pad of Celite and washed with methanol. After evaporation of the solvent *in vacuo*, compound **4** was obtained as purple oil (246.6 g, 97%).
^{1}H NMR (600 MHz, MeOD-d_4) δ = 4.24 (s, 2H), 6.75 (dd, *J* = 2.3, 8.7 Hz, 1H), 7.03 (dd, *J* = 0.5, 2.3 Hz, 1H), 7.24 (dd, *J* = 0.6, 8.7 Hz, 1H), 7.35 (s, 1H) ppm, **(SI Fig. 14)**. ^{13}C NMR (151 MHz, MeOD-d_4) δ = 35.8, 103.0, 107.2, 113.2, 113.4, 127.3, 128.4, 132.9, 152.2 ppm, **(SI Fig. 14)**.
^{1}H NMR (600 MHz, DMSO-d_6) δ = 4.50 (s, 2H), 7.14 (d, *J* = 8.4 Hz, 1H), 7.43 (s, 1H), 7.62 – 7.68 (m, 1H), 7.85 (s, 1H), 8.73 (t, *J* = 24.9 Hz, 2H), 9.24 (br s, 1H), 11.49 (s, 1H) ppm, **(SI Fig. 14)**.

(*E*)-3-(5-(Benzyloxy)-1*H*-indol-3-yl)acrylonitrile (5)

Compound **5** was prepared according to a modified literature procedure[6]. Starting material **2** (1.00 g, 3.99 mmol, 1.0 equiv.) and methyltriphenylphosphonium chloride (2.69 g, 7.94 mmol, 2.0 equiv.) were dissolved in toluene (10 mL) under argon. Next, DBU (1.2 mL, 7.88 mmol, 2.0 equiv.) was added and the reaction mixture was stirred at 115°C until TLC monitoring (1:8 LP:EtOAc) showed full consumption of the starting material after 5.5 hours. After cooling to room temperature, the reaction mixture was distributed between EtOAc (20 mL) and water (20 mL) and the aqueous phase was extracted with EtOAc (4 x 25 mL). The combined organic phase was washed with water (40 mL), dried over anh. Na_2SO_4, filtered, and evaporated to give a brown crude oil. The material was purified by column chromatography over 40 g silica. A gradient of 2:1 LP:EtOAc (300 mL) to 1:1 LP:EtOAc (500 mL) was used. Compound **5** was obtained as slightly yellow solid (768.8 mg, 70%).
mp: 141.4-143.3 °C, ^{1}H NMR (400 MHz, $CDCl_3$-*d*) δ = 5.14 (s, 2H), 5.67 (d, *J* = 16.6 Hz, 1H), 7.03 (dd, *J* = 2.4, 8.9 Hz, 1H), 7.27 (d, *J* = 2.8 Hz, 1H), 7.33 (d, *J* = 8.8 Hz, 1H), 7.34 – 7.37 (m, 1H), 7.39 – 7.44 (m, 2H), 7.47 – 7.50 (m, 3H), 7.52 (d, *J* = 16.6 Hz, 3H), 8.47 (s, 1H) ppm, **(SI Fig. 15)**. ^{13}C NMR (101 MHz, $CDCl_3$-*d*) δ = 71.2, 90.3, 104.1, 112.8, 113.4, 114.3, 120.0, 125.5,

127.7 (2C), 128.2, 128.8 (2C), 129.1, 132.2, 137.3, 143.9, 155.1 ppm, **(SI Fig. 15)**. HRMS calcd. [$C_{18}H_{14}N_2O$-H]$^-$: 273.1033; found: 273.1036.

3-(5-Hydroxy-1*H*-indol-3-yl)propanenitrile (6)

Compound **6** was prepared according to a modified literature procedure[8]. MeOH (90 mL) was added to starting material **5** (1.11 g, 4.06 mmol, 1.0 equiv.) and 10% Pd/C (59.0 mg, 5w %). Hydrogen atmosphere of 1 bar was established and the reaction was stirred at 55°C for 7 hours until TLC monitoring (1:1 LP:EtOAc) showed full conversion. The reaction mixture was filtered over a pad of Celite and washed with methanol. After evaporation of the solvent *in vacuo*, compound **6** was obtained as off-white solid (803.6 mg, 106%, 5w% methanol, 95% purity).
mp: 112.8-115.0 °C,^{1}H NMR (400 MHz, MeOD-d_4) δ = 2.73 (t, *J* = 7.2 Hz, 2H), 3.01 (td, *J* = 1.1, 7.4 Hz, 2H), 6.69 (ddd, *J* = 0.4, 2.3, 8.7 Hz, 1H), 6.90 (dd, *J* = 0.6, 2.3 Hz, 1H), 7.09 (d, *J* = 0.6 Hz, 1H), 7.18 (dd, *J* = 0.6, 8.7 Hz, 1H) ppm, **(SI Fig. 15)**. ^{13}C NMR (101 MHz, MeOD-d_4) δ = 19.0, 22.7, 103.1, 112.3, 112.6, 112.8, 121.3, 124.4, 128.7, 133.1, 151.3 ppm, **(SI Fig. 15)**. HRMS calcd. [$C_{11}H_{10}N_2O$-H]$^-$: 185.0720; found: 185.0719.

3-(3-Aminopropyl)-1*H*-indol-5-ol (7)

Compound **7** was prepared according to modified literature procedures[9,10]. Starting material **6** (64.9 mg, 0.35 mmol, 1.0 equiv. was dissolved in dry THF (3 mL) under argon. The solution was cooled to 0°C and $LiAlH_4$ (69.8 mg, 1.74 mmol, 5.0 equiv.) was slowly added to it. After complete addition and finished H_2 formation, the reaction mixture was heated to 57°C under argon. After 3 hours, TLC monitoring (1:1 LP:EtOAc and MeOH + 1% NH_3) indicated full conversion. The reaction was cooled with a water bath and quenched by slow addition of MeOH. The heterogeneous grey solution was directly applied on a silica column with about 10 g silica and a diameter of 3 cm for purification with MeOH + 1% NH_3 as solvent. After evaporation of the solvent desired compound **7** was obtained as brown to orange oil (52.7 mg, 79%).
^{1}H NMR (400 MHz, MeOD-d_4) δ = 1.86 (p, *J* = 7.4 Hz, 2H), 2.71 (td, *J* = 4.0, 7.3 Hz, 4H), 6.65 (dd, *J* = 2.4, 8.6 Hz, 1H), 6.91 (dd, *J* = 0.5, 2.4 Hz, 1H), 6.96 (s, 1H), 7.14 (dd, *J* = 0.5, 8.6 Hz, 1H) ppm, **(SI Fig. 15)**. ^{13}C NMR (101 MHz, MeOD-d_4) δ = 23.5, 33.8, 42.2, 103.6, 112.2, 112.6, 115.1, 123.7, 129.4, 133.2, 151.0 ppm, **(SI Fig. 15)**. HRMS calcd. [$C_{11}H_{14}N_2O$+H]$^+$: 191.1179; found: 191.1186.

4-(5-(Benzyloxy)-1*H*-indol-3-yl)butanenitrile (8)

Compound **8** was prepared according to a modified literature procedure[11]. 5-(Benzyloxy)-1*H*-indole **1** (1.01 g, 4.54 mmol, 1.0 equiv.) was dissolved in dry toluene (5 mL) and MeMgI (3.0M in Et_2O, 1.65 mL, 4.95 mmol, 1.1 equiv.) was added. The reaction was run for 20 minutes under argon at rt. Then, 4-bromobutyronitrile (450 µL, 4.49 mmol, 1.0 equiv.) was added. The reaction mixture was heated to 70°C and ran for 24 hours. Then, the

reaction was worked up even though TLC (1:4 LP:EtOAc) showed leftover starting material **1**. Satd. NH_4Cl (3 mL) was added to the reaction mixture and the reaction mixture was diluted with water and EtOAc. The aqueous phase was extracted with EtOAc (3 x 50 mL). The combined organic phase was dried over Na_2SO_4, filtered, and evaporated to give a red oil as crude material. The material was purified by column chromatography over 120 g silica with a gradient of 4:1 LP:EtOAc (500 mL) followed by 3:1 LP:EtOAc (800 mL), 2:1 LP:EtOAc (750 mL) and 1:1 LP:EtOAc (500 mL). Pure compound **8** was obtained as pale colourless to yellow oil (373.9 mg, 28%).

^{1}H NMR (600 MHz, DMSO-d_6) δ = 1.90 (p, *J* = 7.2 Hz, 2H), 2.48 (d, *J* = 7.1 Hz, 2H), 2.74 (t, *J* = 7.5 Hz, 2H), 5.09 (s, 2H), 6.80 (dd, *J* = 2.4, 8.7 Hz, 1H), 7.11 (t, *J* = 2.4 Hz, 2H), 7.24 (d, *J* = 8.7 Hz, 1H), 7.29 – 7.34 (m, 1H), 7.39 (t, *J* = 7.5 Hz, 2H), 7.48 (d, *J* = 7.1 Hz, 2H), 10.69 (s, 1H) ppm, **(SI Fig. 15)**. ^{13}C NMR (151 MHz, DMSO-d_6) δ = 16.0, 23.7, 25.6, 69.8, 101.7, 111.7, 112.1, 112.4, 120.8, 123.4, 127.3, 127.6, 127.7 (2C), 128.3 (2C), 131.6, 137.8, 152.0 ppm, **(SI Fig. 15)**. HRMS calcd. $[C_{19}H_{18}N_2O\text{-}H]^-$: 289.1346; found: 289.1345.

4-(5-Hydroxy-1*H*-indol-3-yl)butanenitrile (9)

Compound **8** was prepared according to a modified literature procedure[8]. To starting material **8** (232.8 mg, 0.81 mmol, 1.0 equiv.) and 10% Pd/C (16.2 mg, 7w%), MeOH (15 mL) was added. Hydrogen atmosphere of 1 bar was established and the reaction was stirred at 40°C overnight. As TLC monitoring (1:1 LP:EtOAc) showed leftover starting material **8**, more 10% Pd/C (12.1 mg, 5w%) was added and new hydrogen atmosphere was established several times over the course of six more hours by evacuating the flask followed by flushing it with hydrogen. After that, TLC monitoring confirmed full conversion. The reaction mixture was filtered over a pad of Celite and washed with methanol. After evaporation of the solvent *in vacuo*, compound **9** was obtained as beige oil (172.2 mg, 107%, 7 w% methanol, 90 % purity).

^{1}H NMR (600 MHz, DMSO-d_6) δ = 1.88 (p, *J* = 7.2 Hz, 2H), 2.49 (d, *J* = 7.3 Hz, 2H), 2.69 (t, *J* = 7.5 Hz, 2H), 6.59 (dd, *J* = 2.1, 8.6 Hz, 1H), 6.80 (d, *J* = 1.9 Hz, 1H), 7.04 (d, *J* = 1.7 Hz, 1H), 7.12 (d, *J* = 8.6 Hz, 1H), 8.60 (s, 1H), 10.51 (s, 1H) ppm, **(SI Fig. 15)**. ^{13}C NMR (151 MHz, DMSO-d_6) δ = 16.0, 23.8, 25.6, 102.1, 111.3, 111.7, 111.8, 120.7, 123.1, 127.6, 130.9, 150.2 ppm, **(SI Fig. 15)**. HRMS calcd. $[C_{12}H_{12}N_2O+H]^+$: 201.1022; found: 201.1024.

3-(4-Aminobutyl)-1*H*-indol-5-ol (10)

Compound **10** was prepared according to modified literature procedures[9,10]. Starting material **9** (58.8 mg, 0.29 mmol, 1.0 equiv. was dissolved in dry THF (2.5 mL) under argon. The solution was cooled to 0°C and $LiAlH_4$ (56.0 mg, 1.48 mmol, 5.0 equiv.) was slowly added to it. After complete addition and finished H_2 formation, the reaction mixture was heated to 55°C under argon. After 3 hours TLC monitoring (1:1 LP:EtOAc and MeOH + 1% NH_3) showed full conversion. The reaction was cooled with a water bath and quenched by slowly adding MeOH. The heterogeneous grey solution was directly applied on a silica column with about 10 g silica and a diameter of 3 cm for purification with MeOH + 1% NH_3 as solvent. After

evaporation of the solvent *in vacuo* desired compound **10** was obtained as yellow to orange oil (33.2 mg, 55%, 4w% methanol, 90% purity).

^{1}H NMR (600 MHz, DMSO-d_6) δ = 1.39 (p, *J* = 7.2, 14.4 Hz, 2H), 1.60 (p, *J* = 7.6 Hz, 2H), 2.55 (dt, *J* = 7.2, 10.2 Hz, 4H), 6.56 (dd, *J* = 2.3, 8.6 Hz, 1H), 6.77 (d, *J* = 2.3 Hz, 1H), 6.97 (d, *J* = 2.3 Hz, 1H), 7.09 (d, *J* = 8.6 Hz, 1H), 8.54 (s, 1H), 10.34 – 10.42 (m, 1H) ppm, **(SI Fig. 15)**. ^{13}C NMR (151 MHz, DMSO-d_6) δ = 24.8, 27.3, 33.5, 41.7, 102.3, 111.0, 111.6, 113.8, 122.6, 127.9, 130.9, 150.0 ppm, **(SI Fig. 15)**. HRMS calcd. $[C_{12}H_{16}N_2O+H]^+$: 205.1335; found: 205.1341.

^{1}H AND EITHER ^{13}C NMR OR APT NMR SPECTRA

5-(Benzyloxy)-1*H*-indole-3-carbaldehyde (2) – ^{1}H and ^{13}C spectra

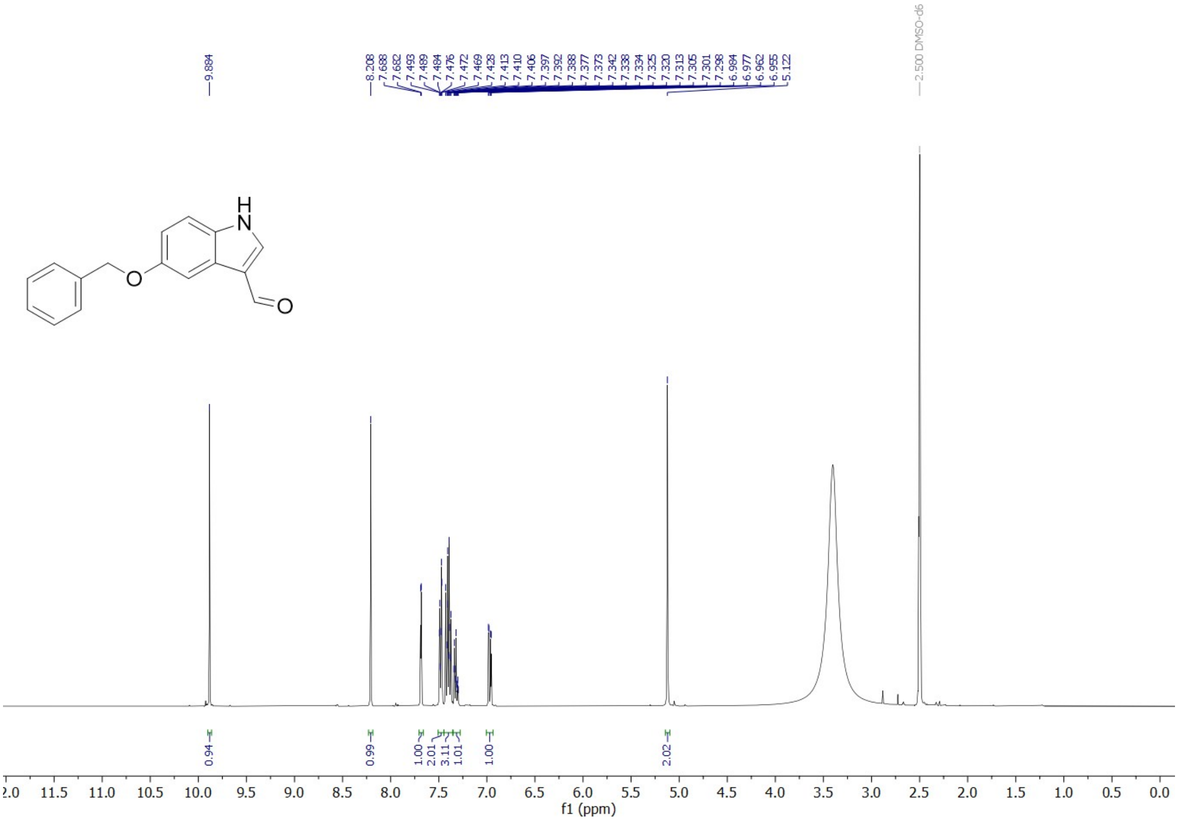

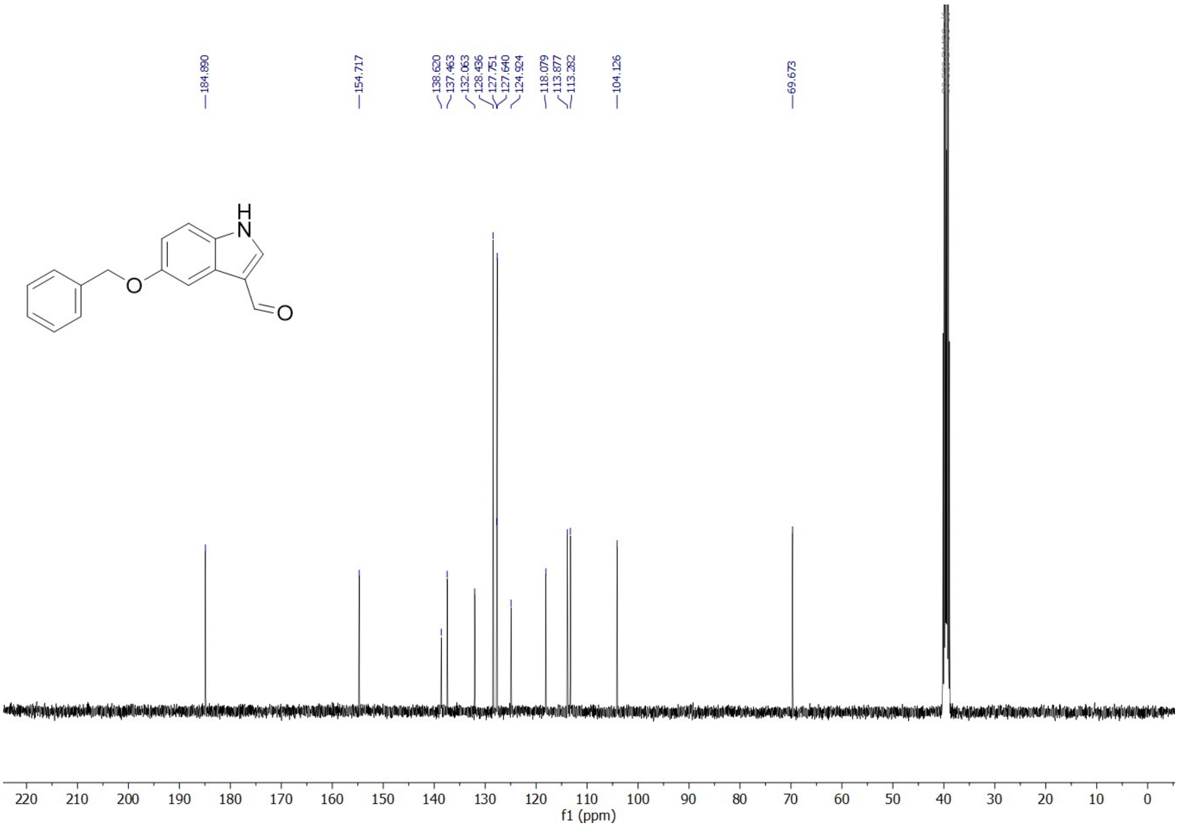

(*E*)-5-(Benzyloxy)-1*H*-indole-3-carbaldehyde oxime (3) – ^{1}H and APT spectra

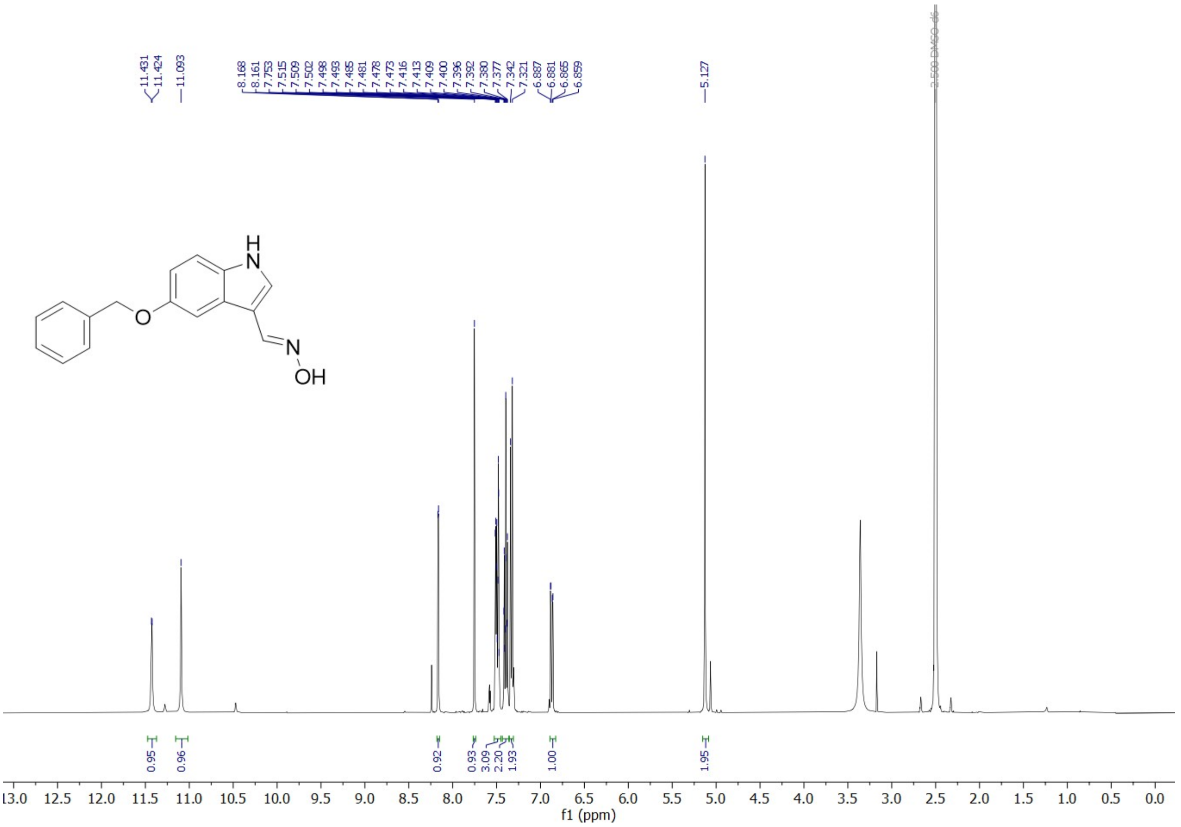

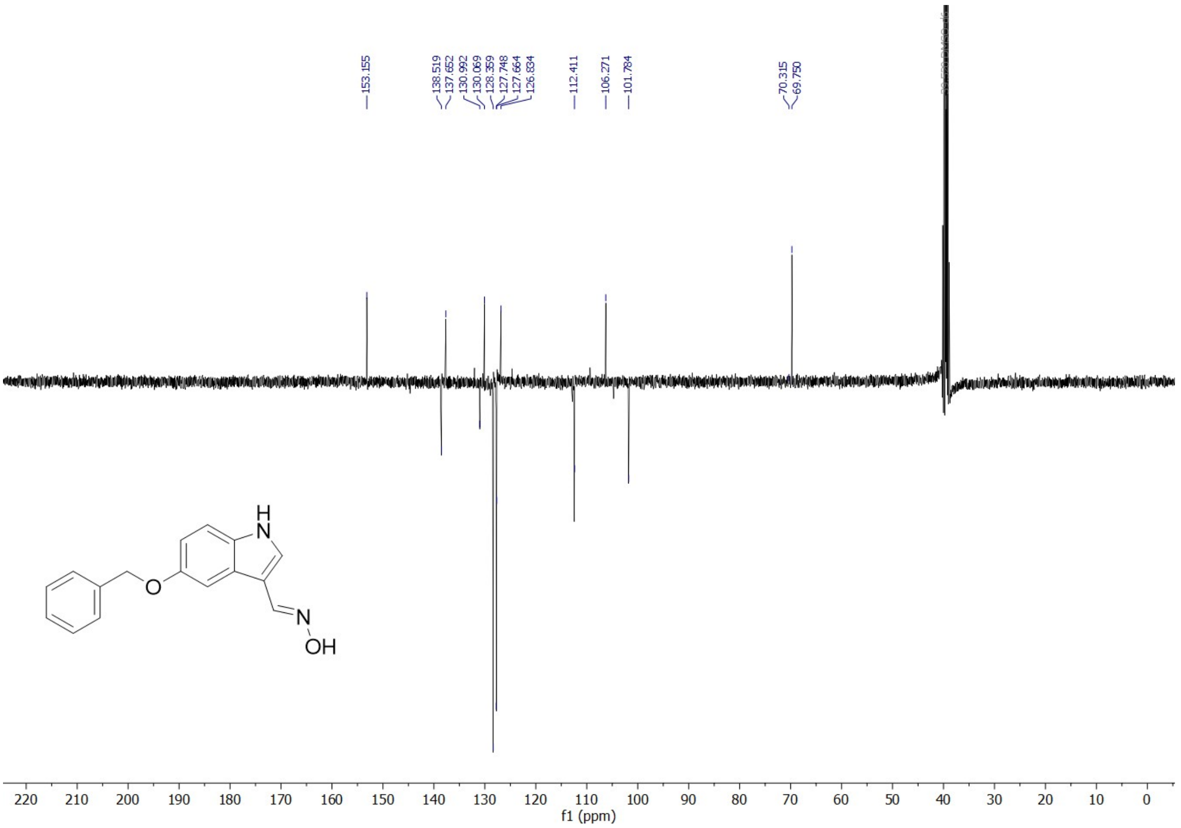

3-(Aminomethyl)-1*H*-indol-5-ol hydrochloride (4) – ^{1}H and APT (MeOD-d$_4$), ^{1}H (DMSO-d$_6$) spectra

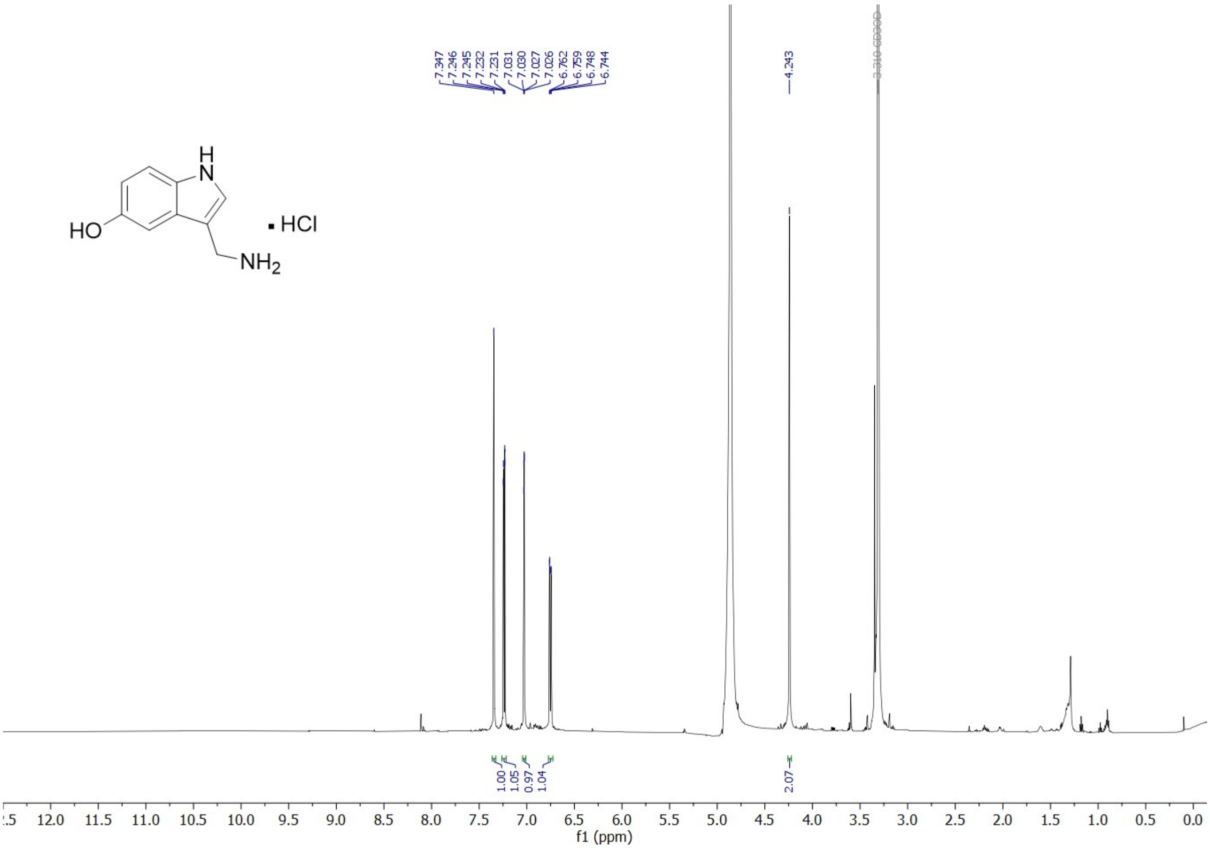

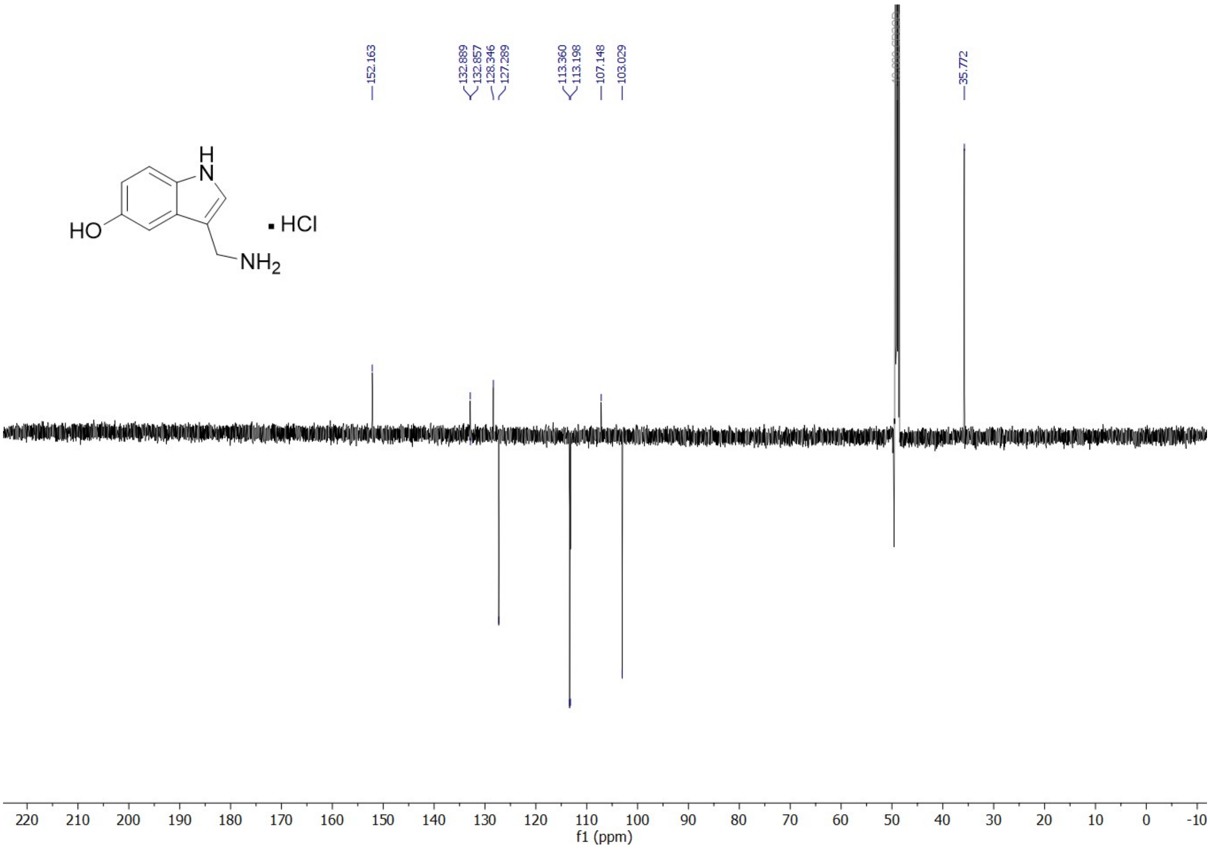

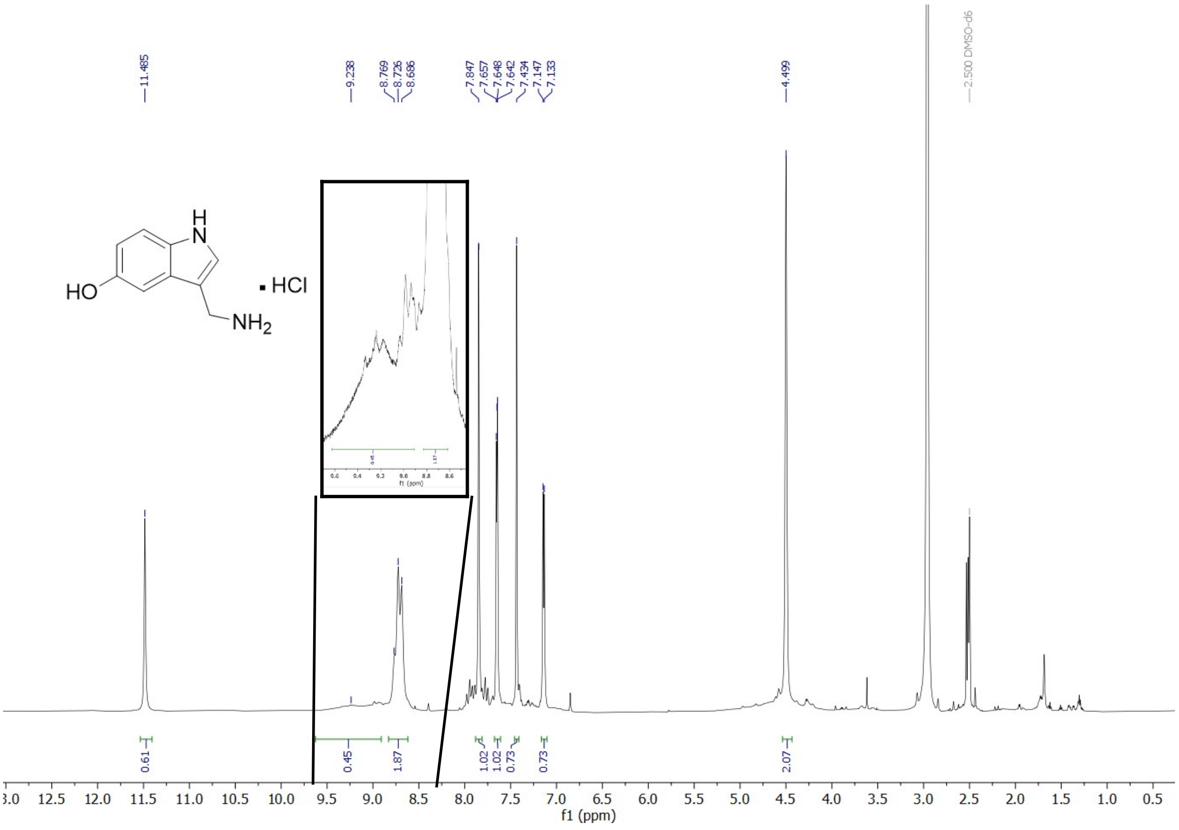

Supplementary Figure 14: ^{1}H and ^{13}C NMR and APT NMR spectra of the intermediate compounds 2, 3, and of M-5HT (compound 4).

Note: The ^{1}H spectrum **(SI Fig. 14)** of compound **4** in DMSO-d6 is provided to show the signals of the indole-NH and the sidechain NH_2, as they are not visible in the ^{1}H (MeOD-d_4) spectrum. The OH-signal (9.24 ppm, (br s)) remains hardly visible even in DMSO-d_6. This can be attributed to the fact that, depending on the NMR solvent and the state of the sample, deuterium exchange between compound **4** and the NMR solvent was observed **(SI Fig. 14)**. Therefore, some of the integrals in the ^{1}H (DMSO-d_6) spectrum are lower as they should be. The mentioned deuterium exchange also leads to a ^{13}C-NMR spectrum in DMSO-d_6 with complex coupling patterns. As the APT spectrum of compound **4** in MeOD-d_4 allows for assignment of all peaks, the ^{13}C spectrum in DMSO-d_6 is not provided here.

(*E*)-3-(5-(Benzyloxy)-1*H*-indol-3-yl)acrylonitrile (5) - ^{1}H and ^{13}C spectra

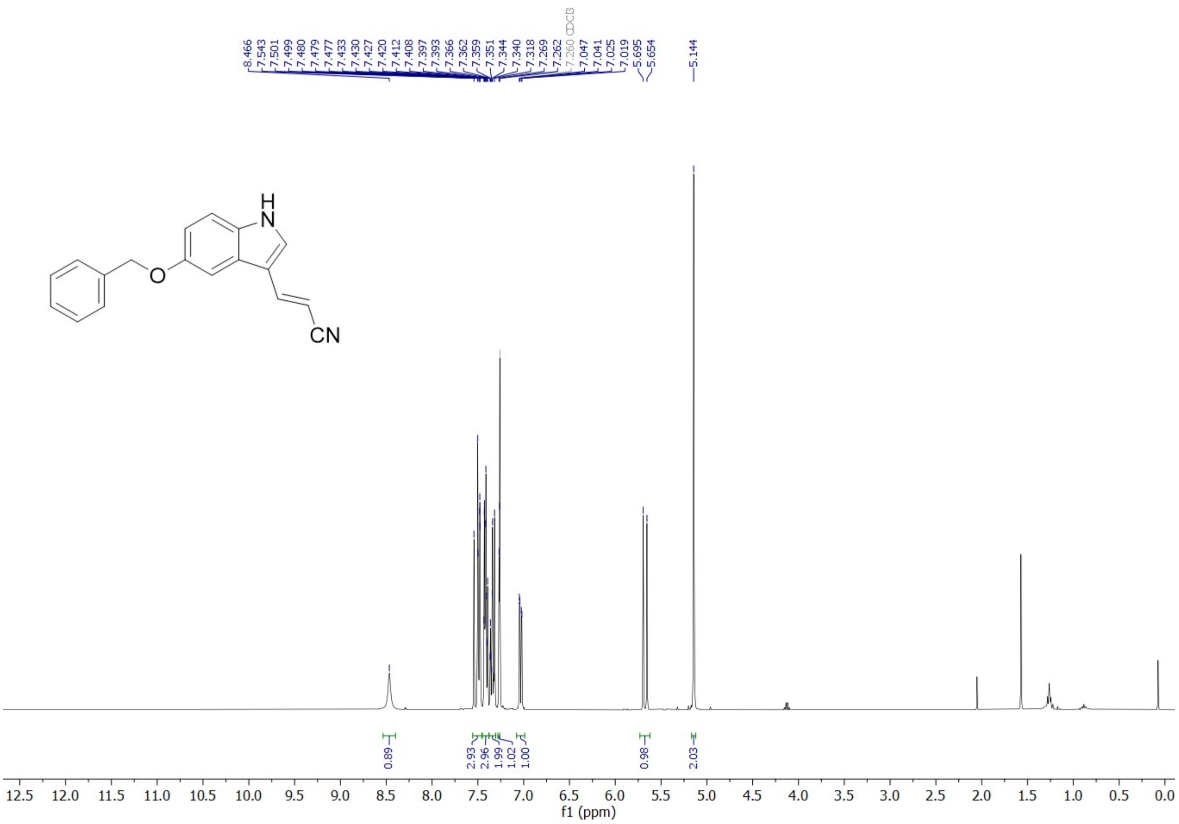

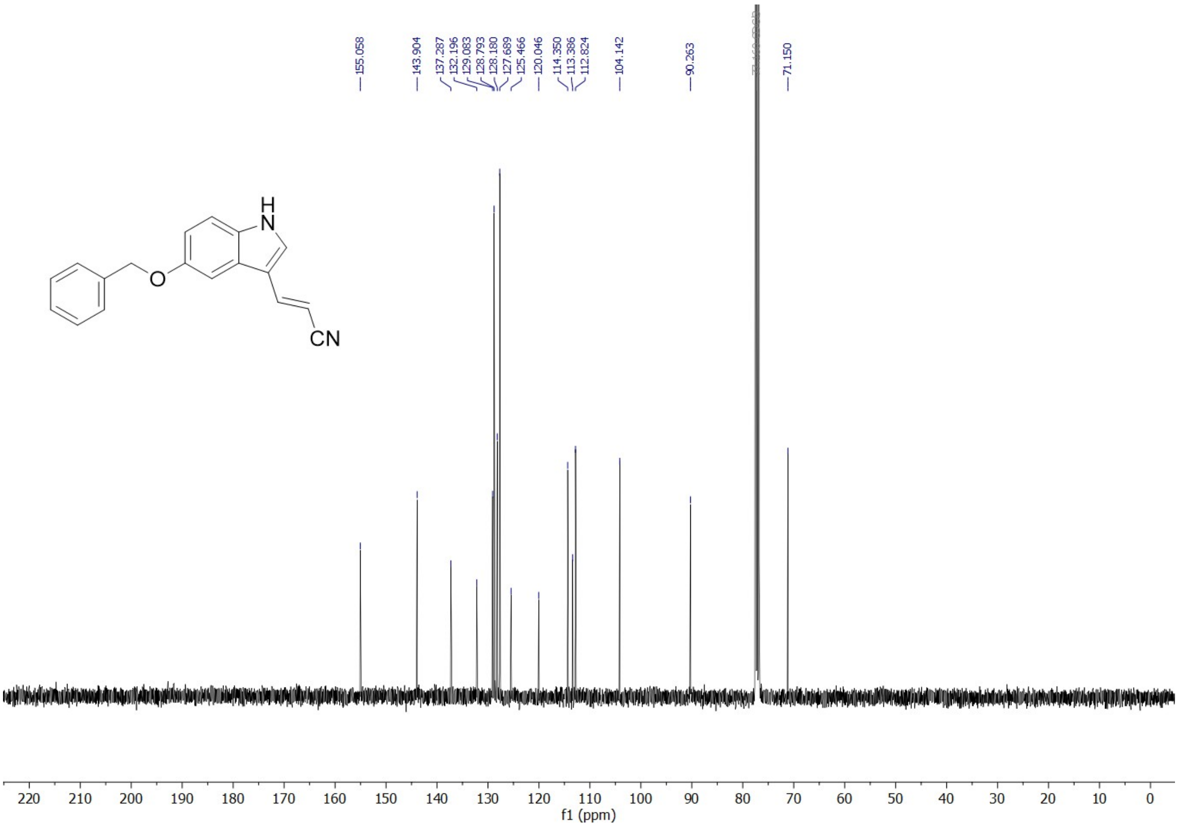

3-(5-Hydroxy-1*H*-indol-3-yl)propanenitrile (6) - ^{1}H and APT spectra

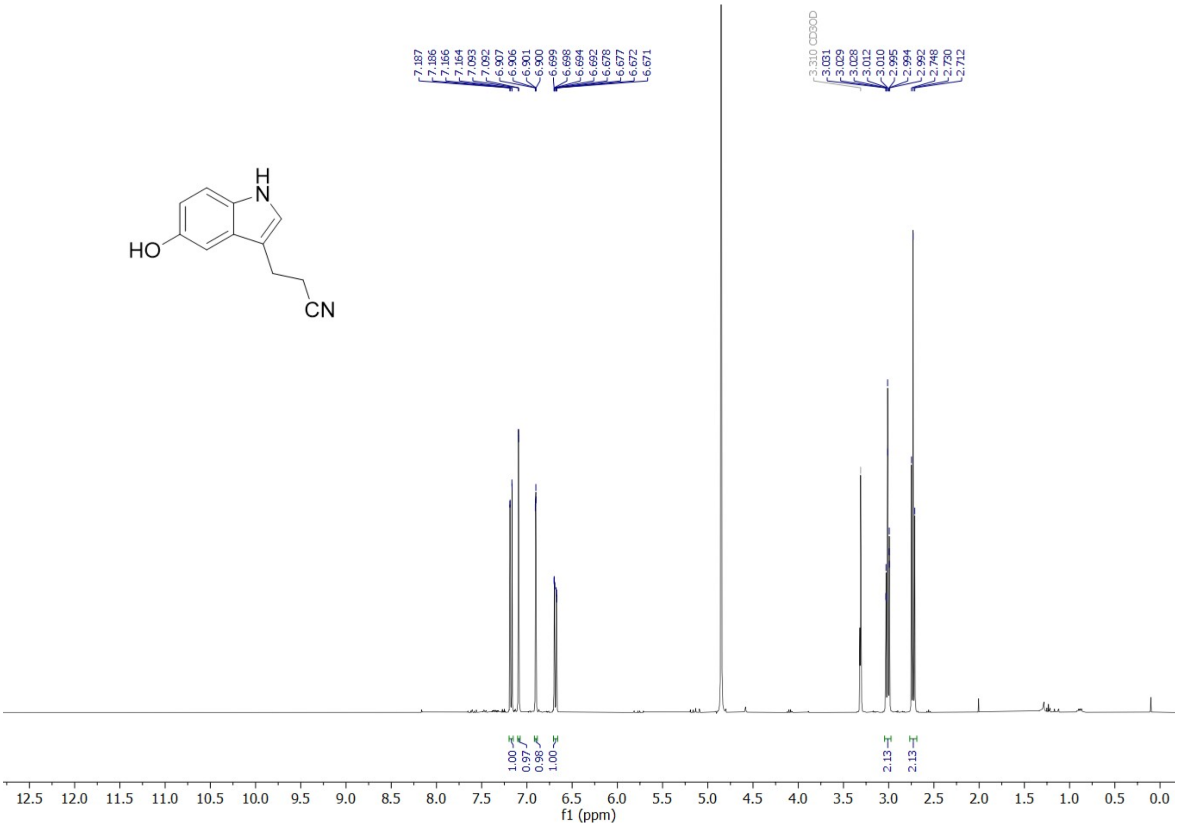

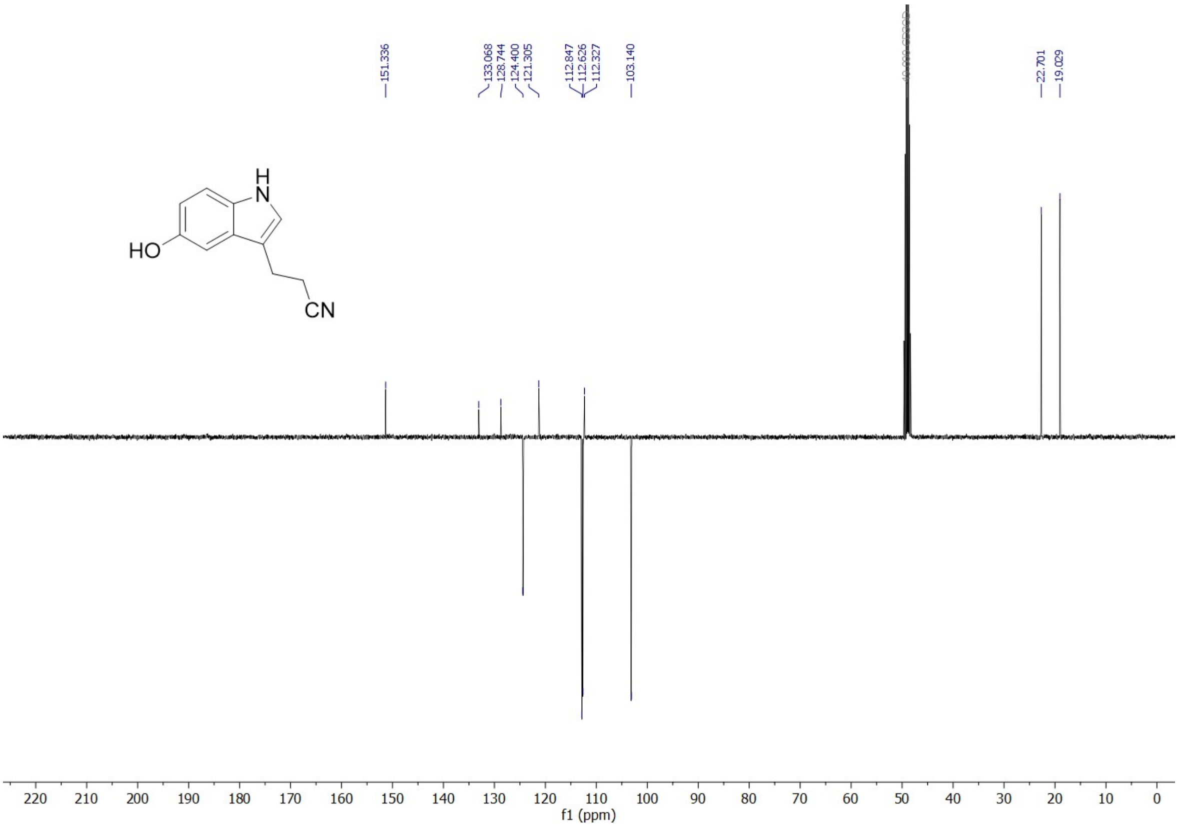

3-(3-Aminopropyl)-1*H*-indol-5-ol (7) - ^{1}H and APT spectra

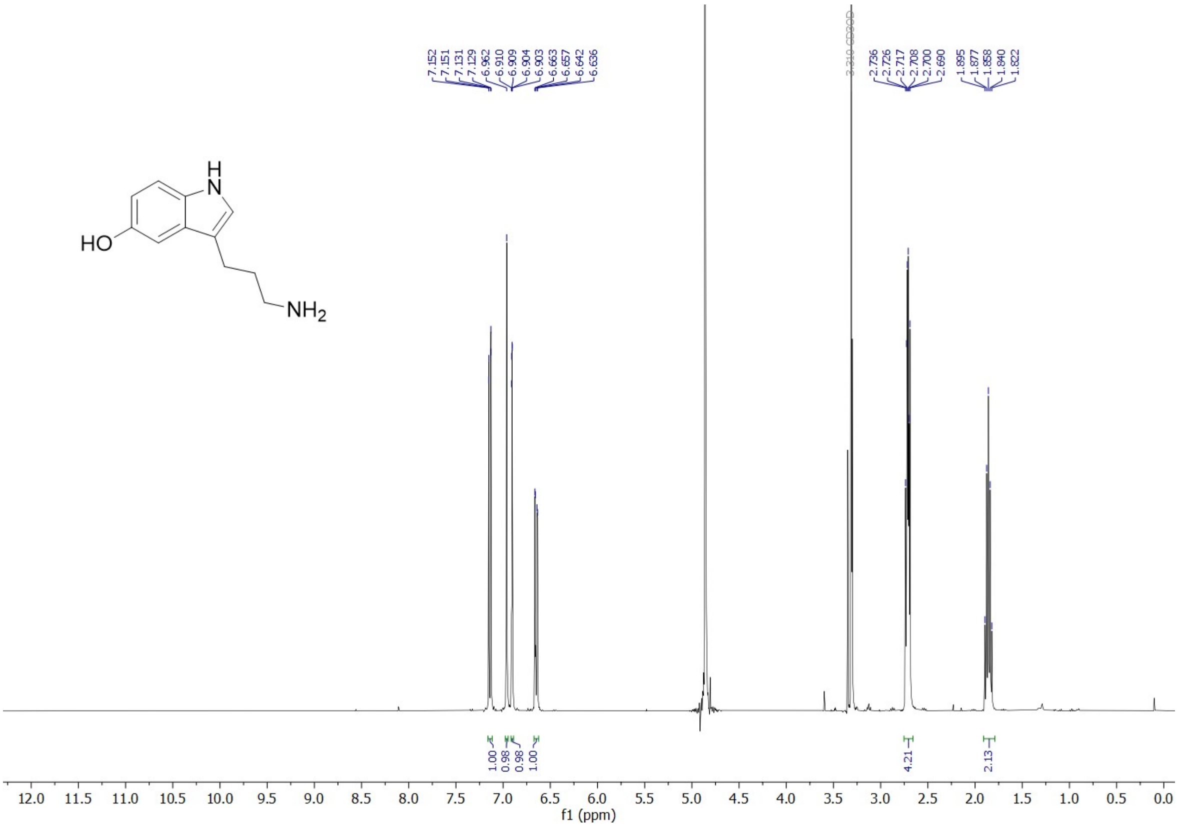

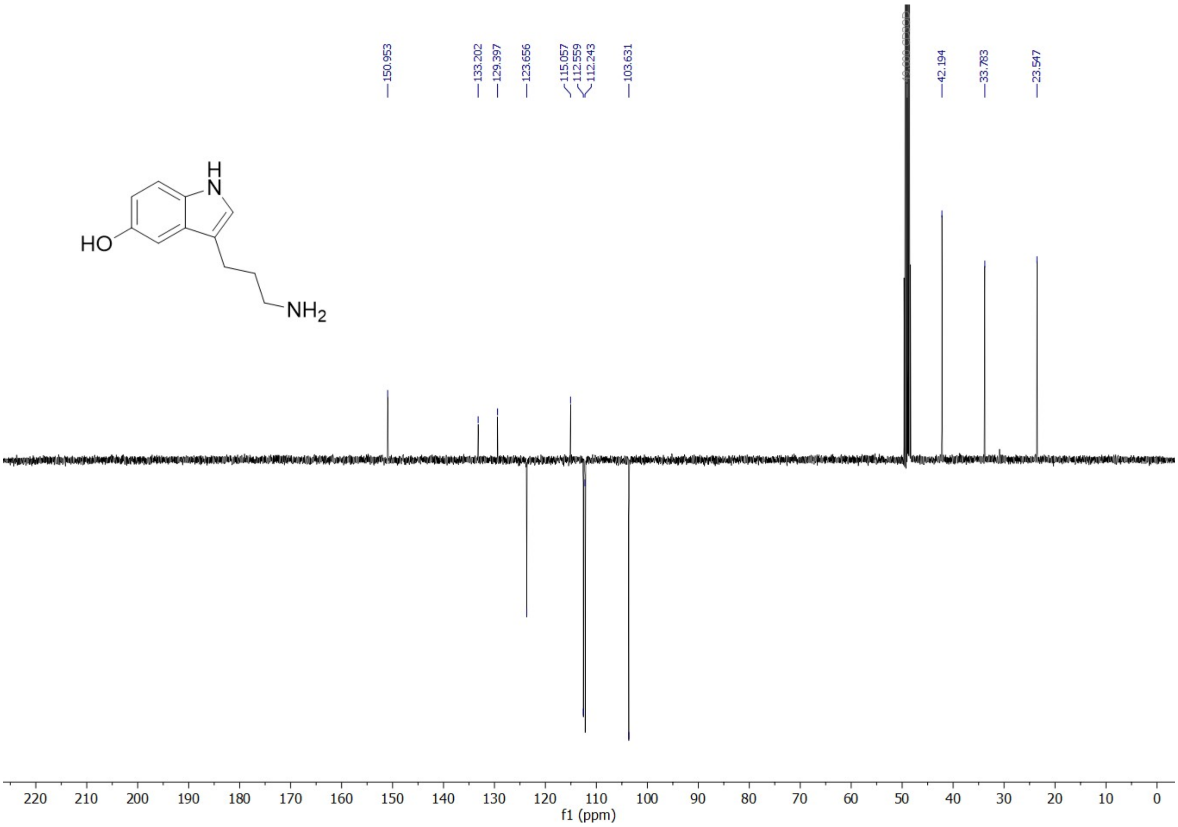

4-(5-(Benzyloxy)-1*H*-indol-3-yl)butanenitrile (8) - ^{1}H and APT spectra

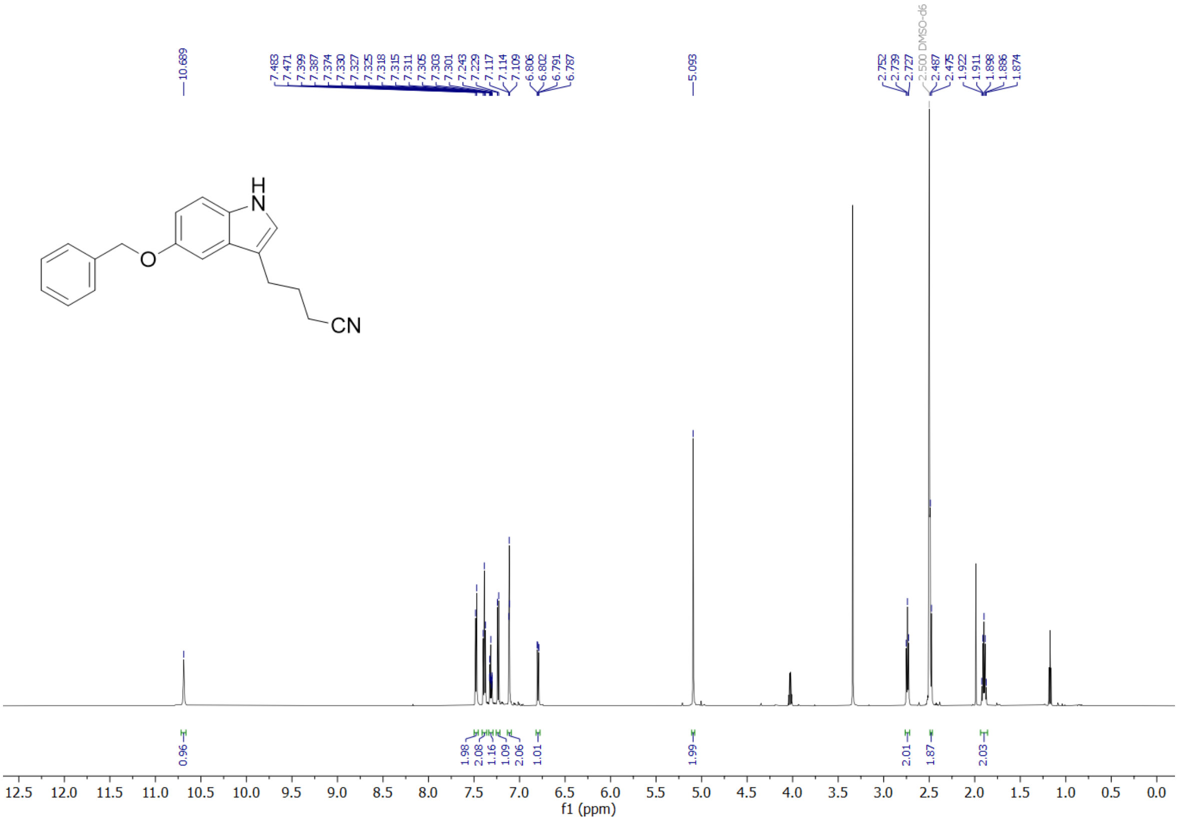

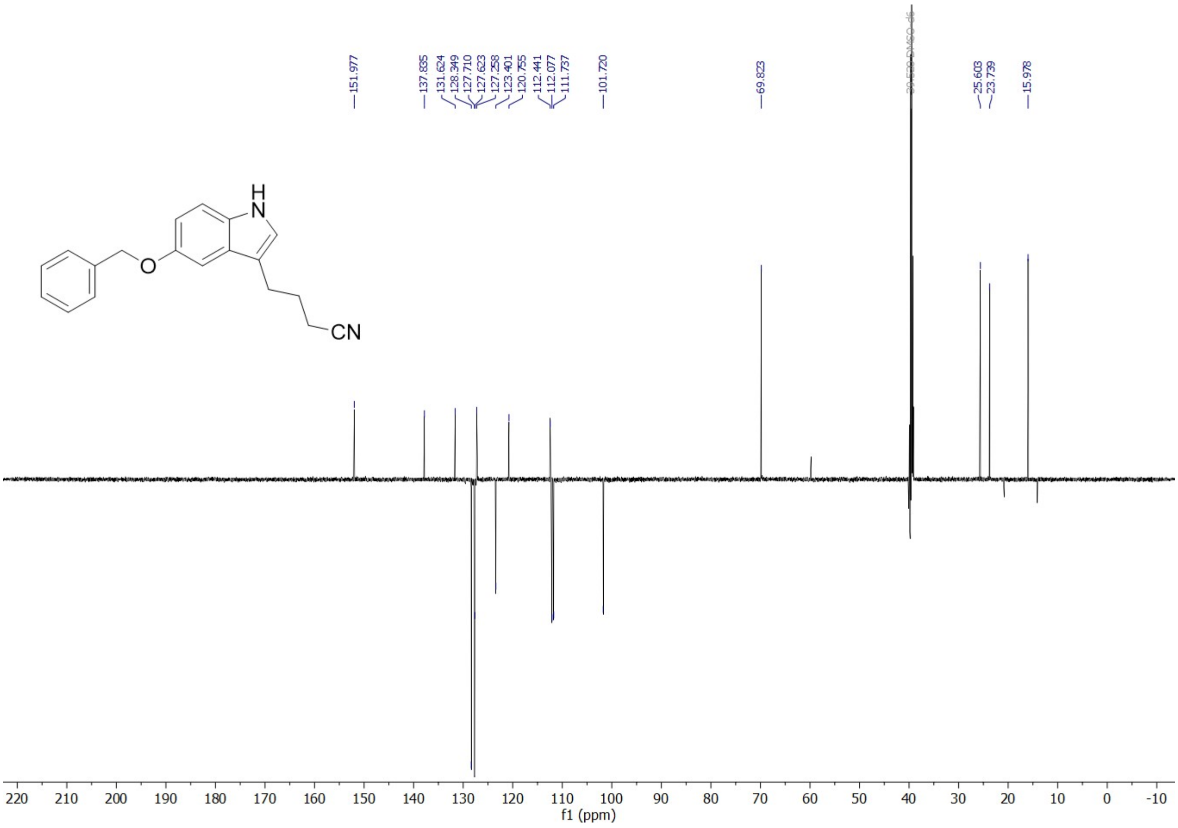

4-(5-Hydroxy-1*H*-indol-3-yl)butanenitrile (9) – ¹H and APT spectra

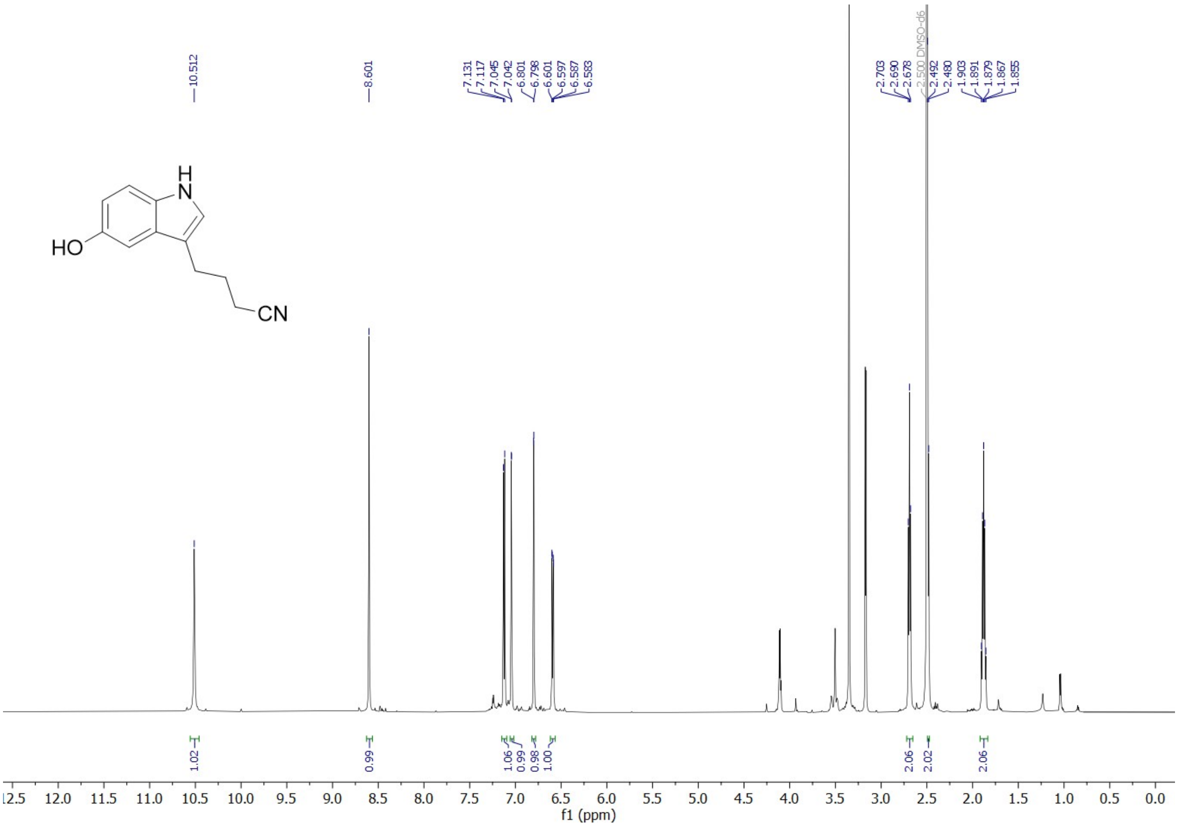

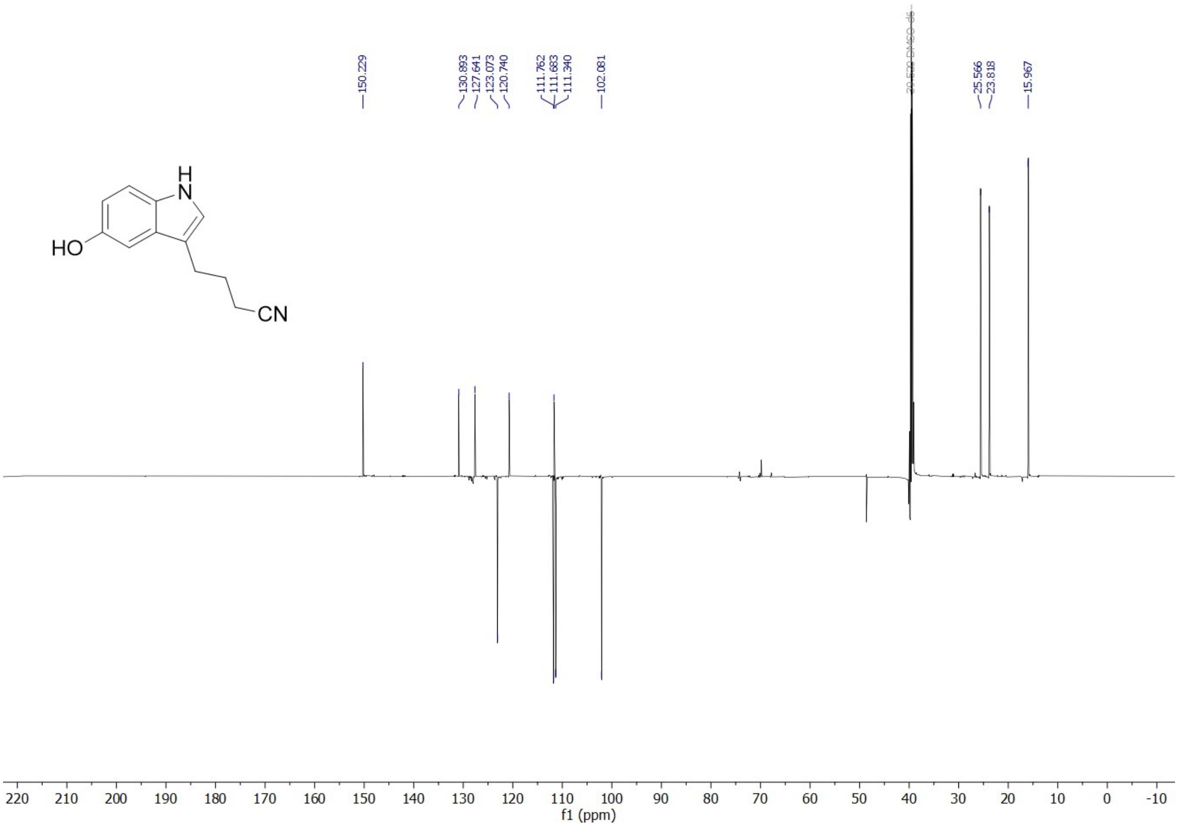

3-(4-Aminobutyl)-1*H*-indol-5-ol (10) – ^{1}H and APT spectra

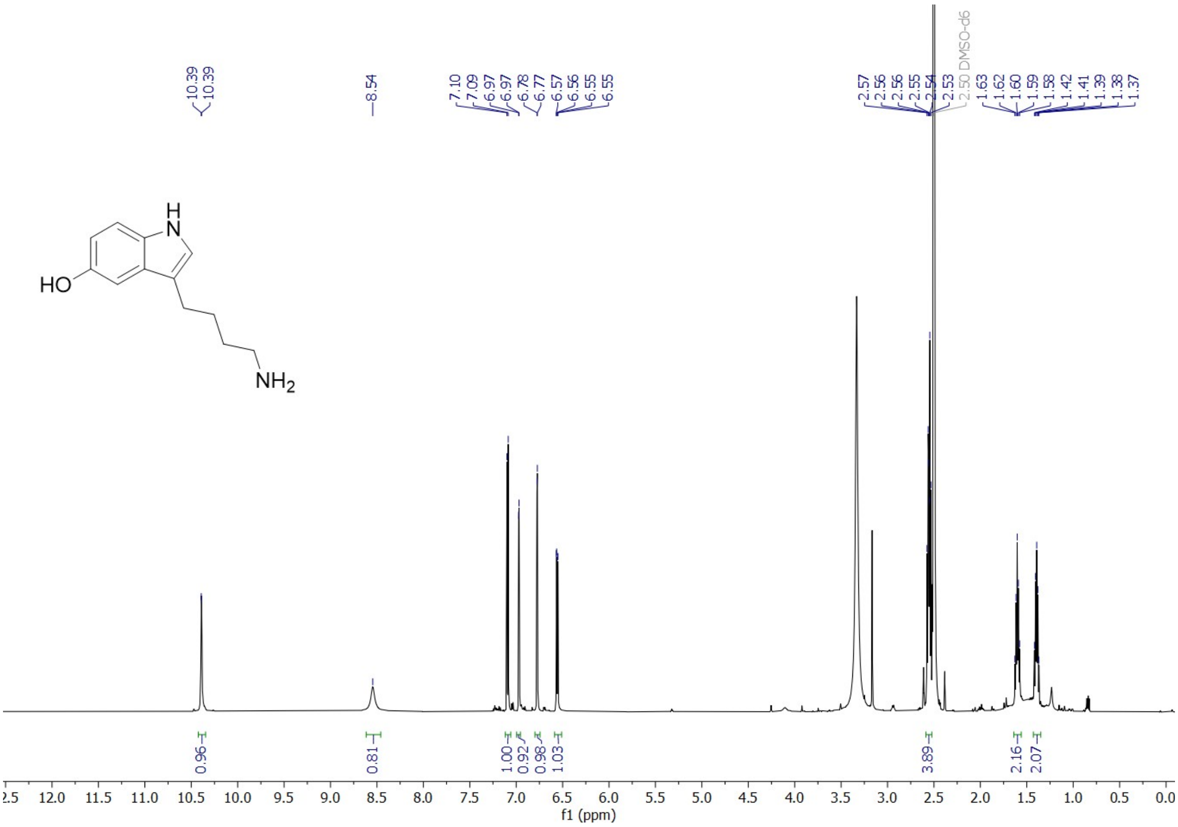

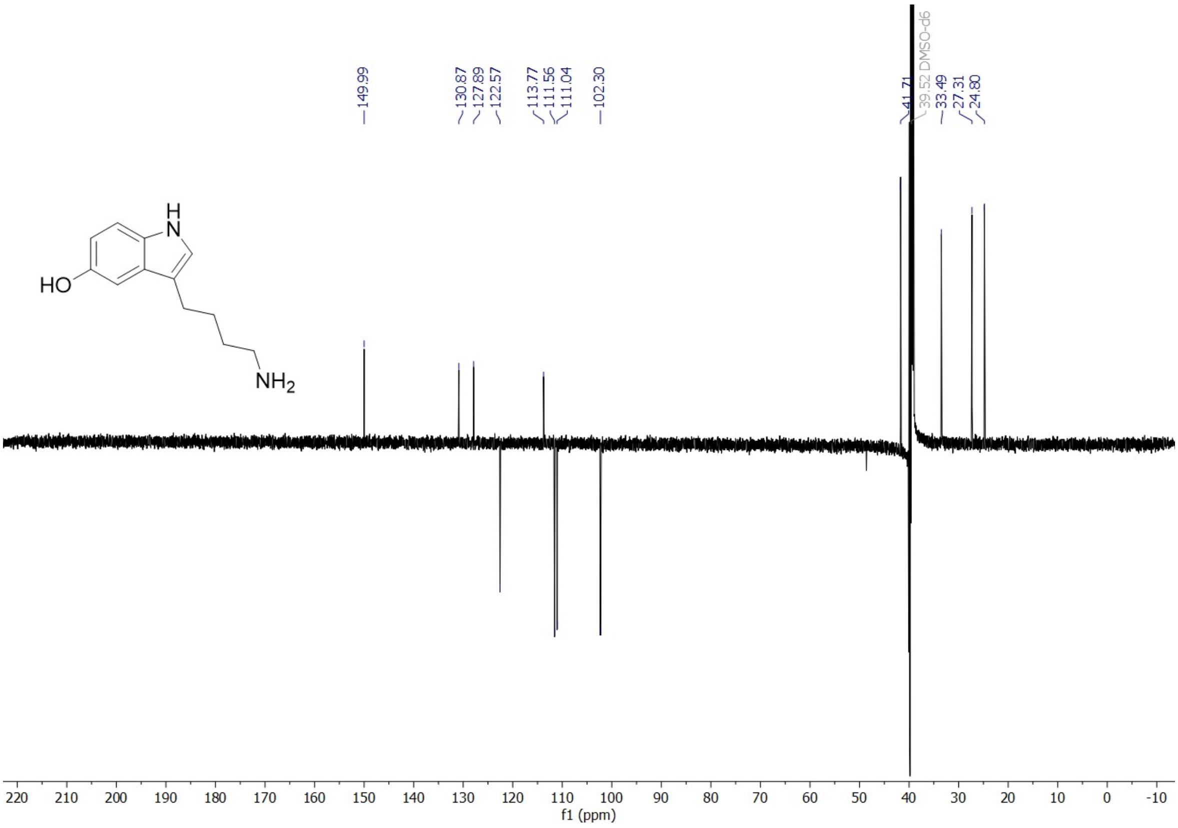

Supplementary Figure 15: ^{1}H and ^{13}C NMR and APT NMR spectra of the intermediate compounds 5, 6, 8, 9, P-5HT (compound 7), and B-5HT (compound 10).

STABILITY STUDIES OF 5HT-DERIVATIVES

As serotonin, as many substrates with free amine and free hydroxyl groups, is known to be a rather unstable compound, we set out to investigate the stability of our synthesised compounds M-5HT (**4**), P-5HT (**7**) and B-5HT (**10**). It was important to us to establish their stabilities to be more confident not only in storage of the pure compounds but also storage and reuse of sample stock solutions for *in vitro* testing.

Therefore, we conducted stability studies by NMR in several solvents like D_2O and DMSO-d_6. Most importantly, we wanted to check if the samples would show any signs of degradation like non-compound related peaks appearing or decreasing peak integrals in comparison to the solvent peak. NMR samples of the three compounds in the desired solvents were prepared and the same samples in the same tubes remeasured over a timeframe of up to four months. For all the following NMR spectra the integral of the proton H2 peak is set to one.

3-(Aminomethyl)-1*H*-indol-5-ol hydrochloride (**4**)

Samples of compound M-5HT (**4**) in D_2O and DMSO-d_6 were prepared and ^{1}H-NMRs measured immediately, after 1 week, 2 weeks, 5 weeks, and 4 months.

Stability study in D_2O

SI Fig. 16 shows the obtained stacked spectra for measurement in D_2O. As it can be seen, no additional peaks occur during this time period. However, especially after 4 months, we can observe a decrease in the peak integrals compared to the solvent peak. Moreover, even though the integral for the singlet at 7.44 ppm (H2) still shows an integral of one, the peaks at 7.11 ppm (H4) and 6.84 ppm (H6) show diminished integrals, accounting for deuterium exchange with the deuterated NMR solvent **(SI Fig. 16a-e)**.

Nevertheless, M-5HT (**4**) shows good stability in D_2O for at least two weeks. Looking at the full ^{1}H spectra, we see a slight increase in the integral of the solvent peak after five weeks. This points towards either minor degradation to material not visible in the NMR or precipitation of the compound.

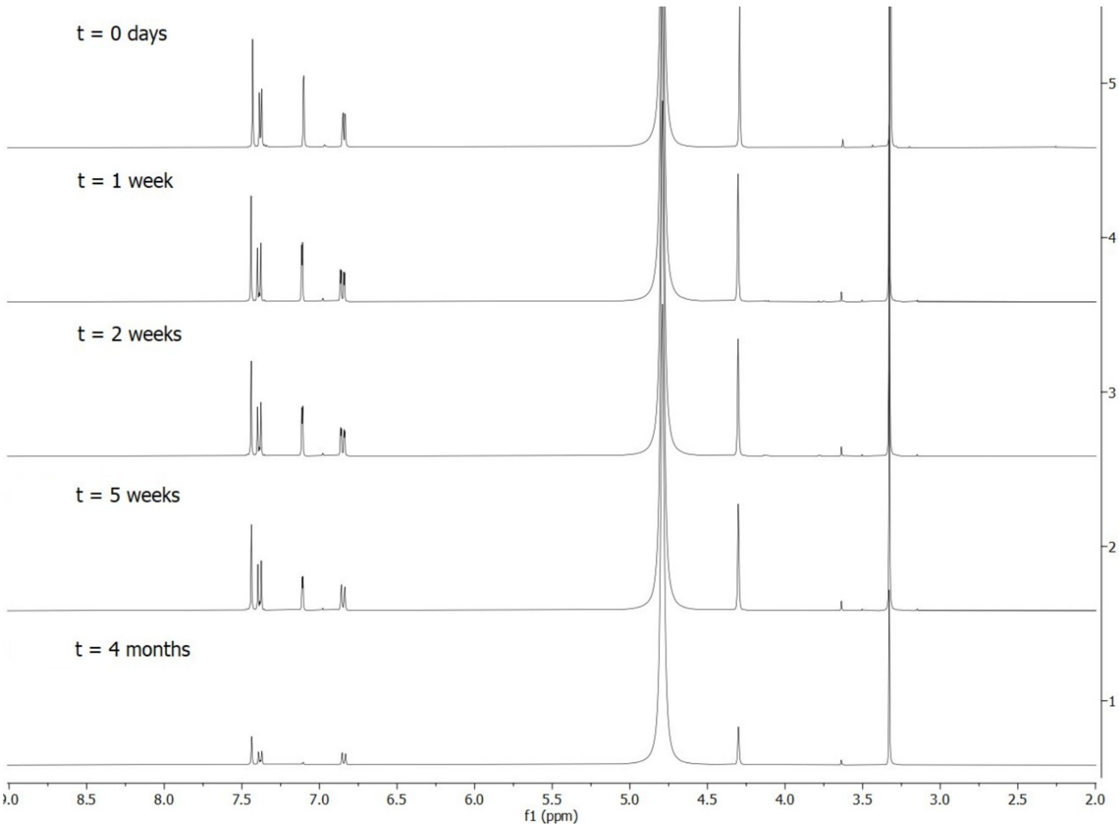

Supplementary Figure 16: Stacked ^{1}H-NMR spectra of compound M-5HT (4) measured after up to 4 months in D_2O. *The individual spectra are expanded in SI Fig. 16a-e.*

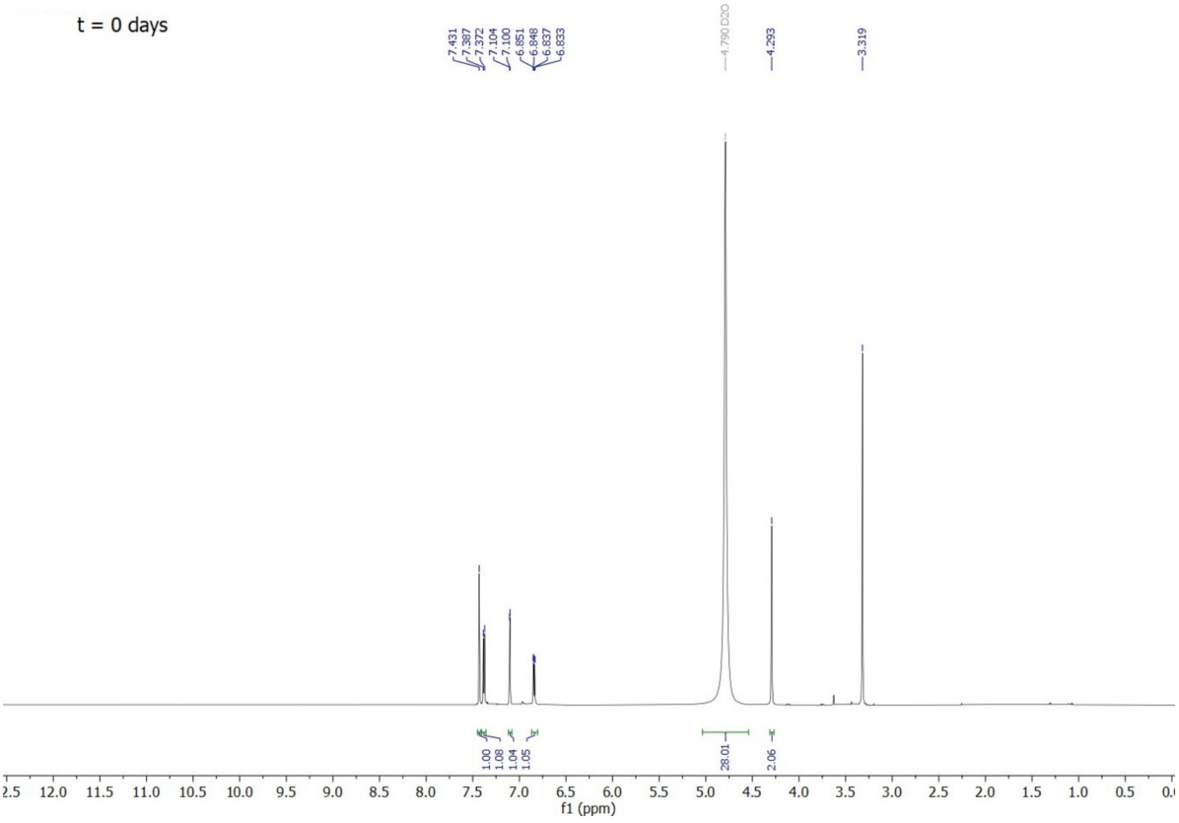

Supplementary Figure 16a: ^{1}H-NMR spectra of compound M-5HT (4) freshly prepared in D_2O.

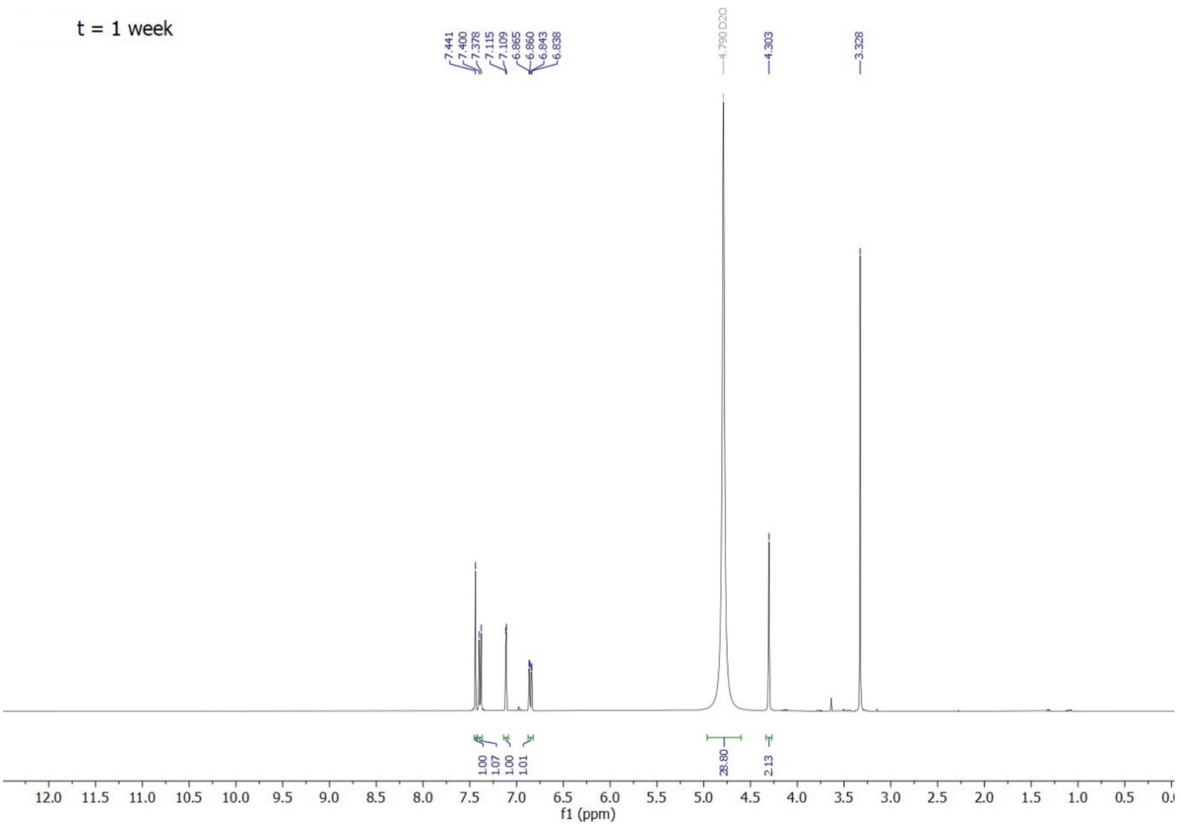

Supplementary Figure 16b: ^{1}H-NMR spectra of compound M-5HT (4) in D_2O after 1 week.

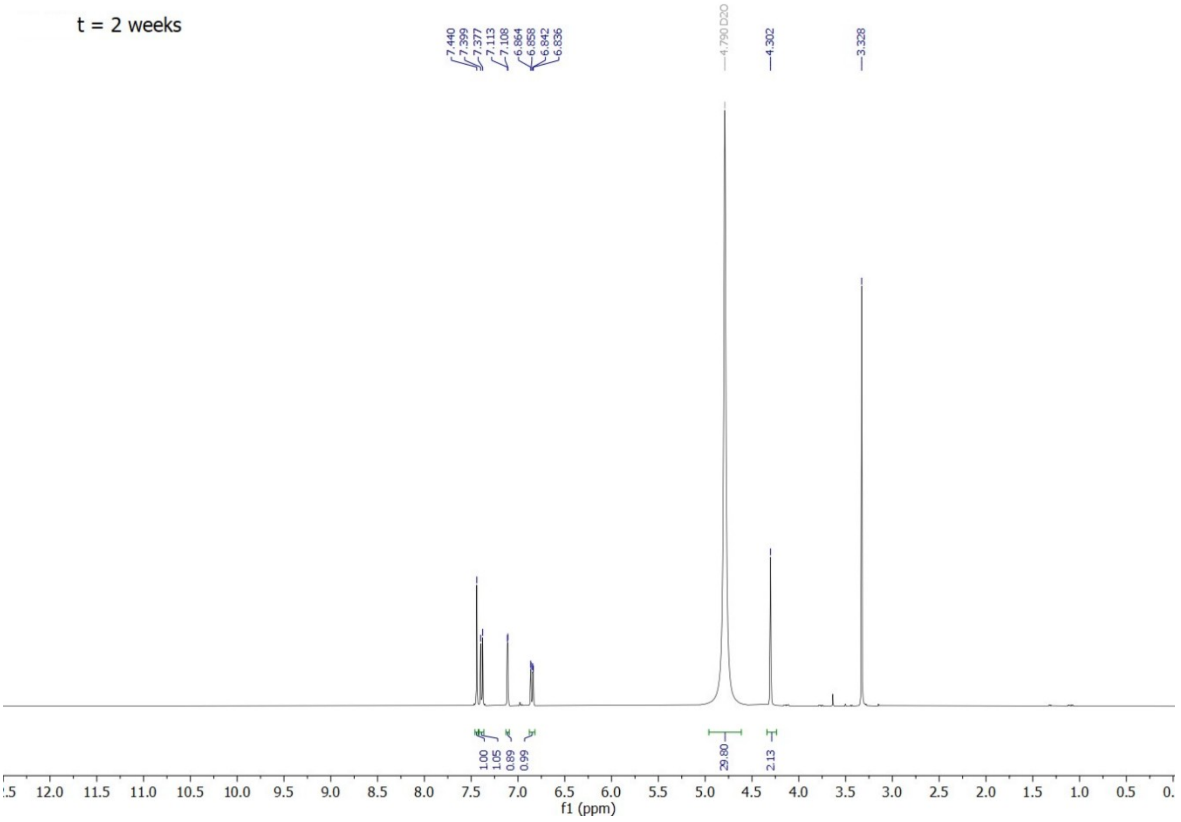

Supplementary Figure 16c: ^{1}H-NMR spectra of compound M-5HT (4) in D_2O after 2 weeks.

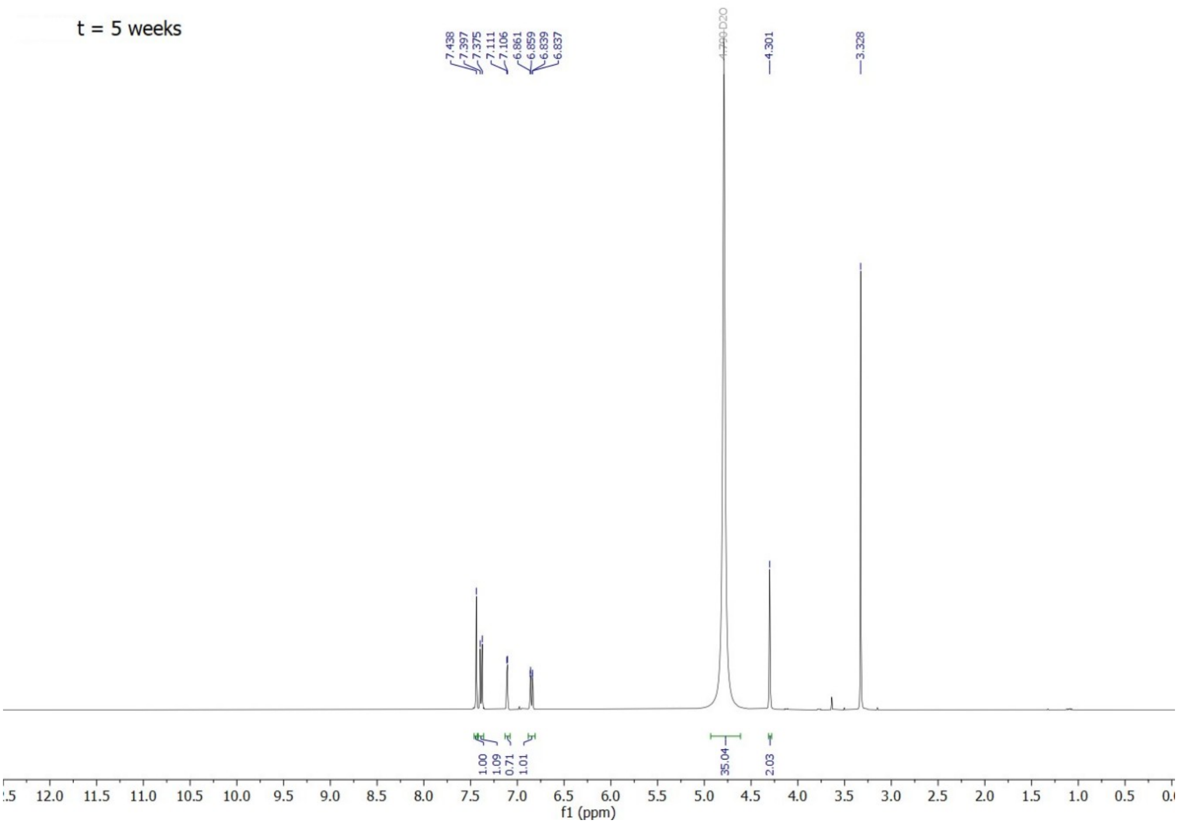

Supplementary Figure 16d: ^{1}H-NMR spectra of compound M-5HT (4) in D_2O after 5 weeks.

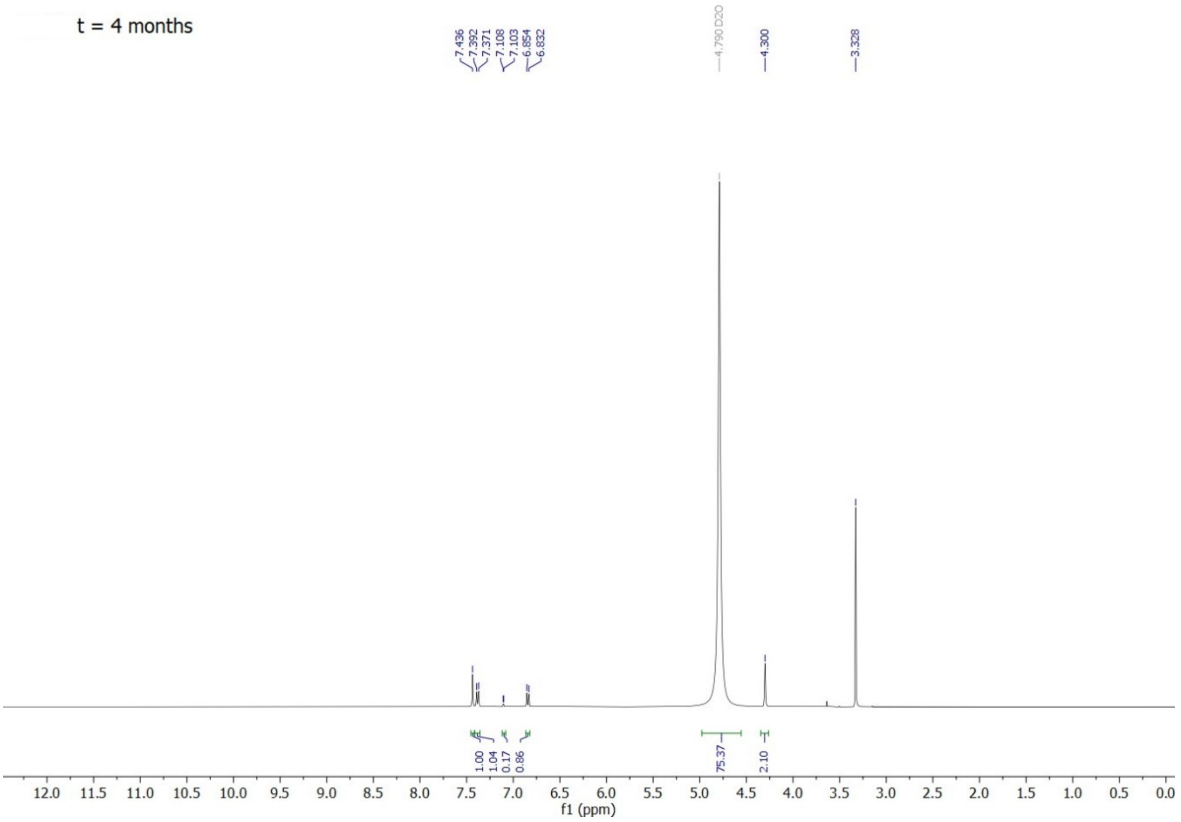

Supplementary Figure 16e: ^{1}H-NMR spectra of compound M-5HT (4) in D_2O after 4 months.

Stability study in DMSO-d_6

The NMR sample of M-5HT (**4**) in DMSO was re-measured after 1.5 weeks, 2.5 weeks, 5.5 weeks, and 4 months. Again, no new peaks appear during this time except for an increasing water peak as can be seen in SI Fig. 17. Like the measurements in D_2O, deuterium exchange can be observed. This also explains the seemingly rapidly growing solvent peak comparing day 0 and week 1.5, which would normally suggest precipitation of the sample. However, the integral of the methanol peak in the sample staying consistent throughout all measurements and the DMSO-d_6 peak integral not changing much any more after reaching 2.77 at 1.5 weeks, indicate that the sample is not precipitating but deuterium exchange happens **(SI Fig. 17a-e)**.

Therefore, the stability of sample M-5HT (**4**) in DMSO-d_6 could also be shown to be very good with hardly any changes in the NMR spectra for 5.5 weeks.

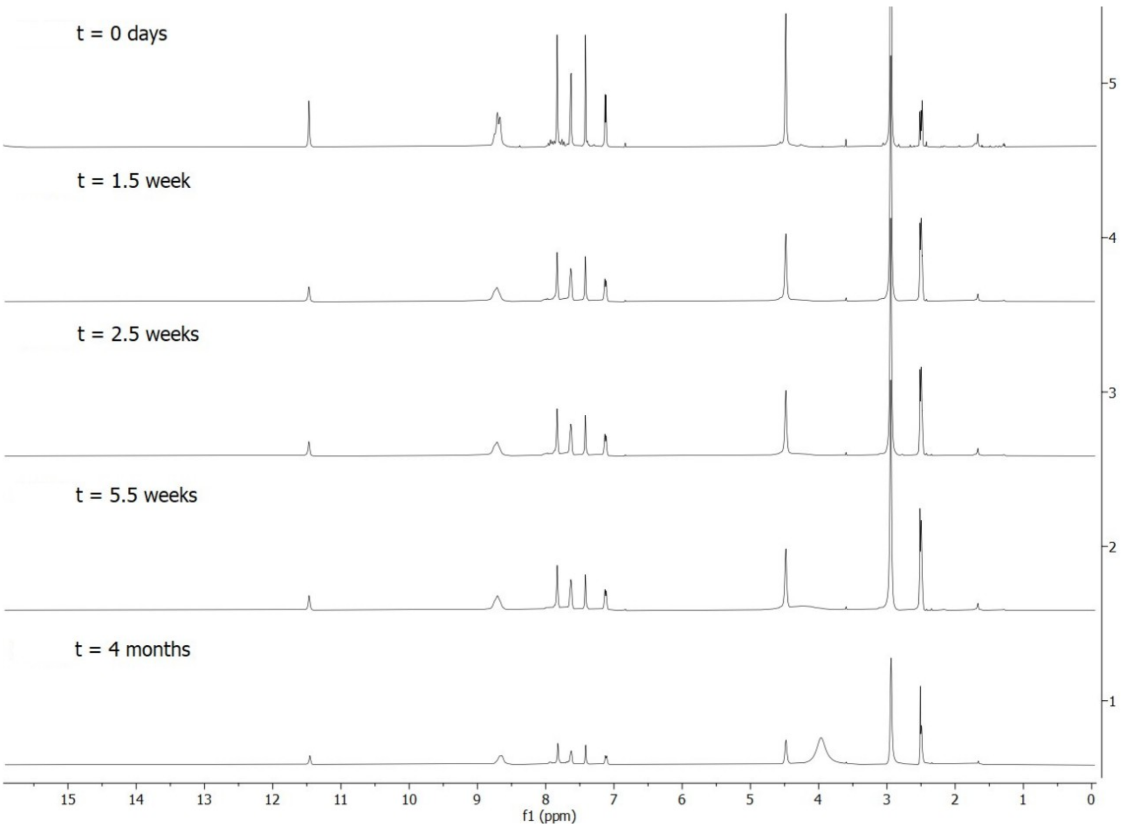

Supplementary Figure 17: Stacked ^{1}H-NMR spectra of compound M-5HT (4) measured after up to 4 months in DMSO-d$_6$. *The individual spectra are expanded in SI Fig. 17a-e.*

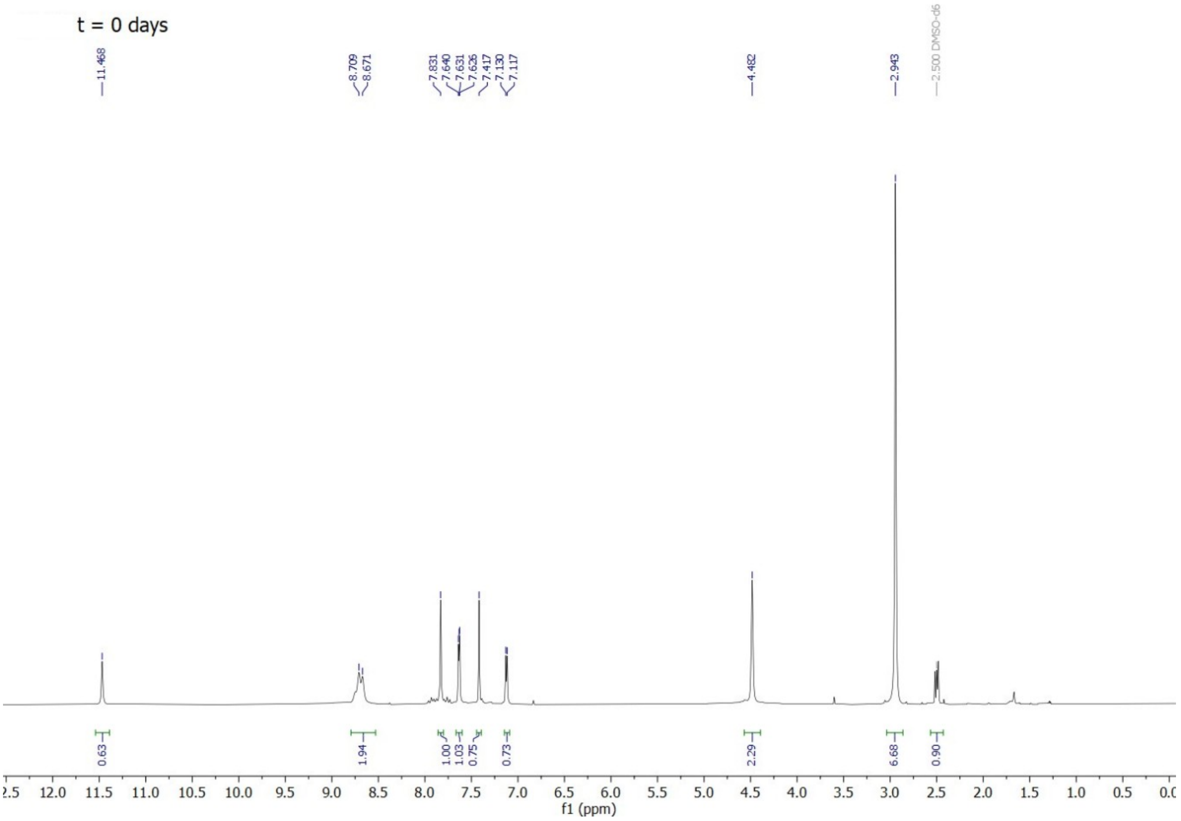

Supplementary Figure 17a: ^{1}H-NMR spectra of compound M-5HT (4) in DMSO-d$_6$ freshly prepared.

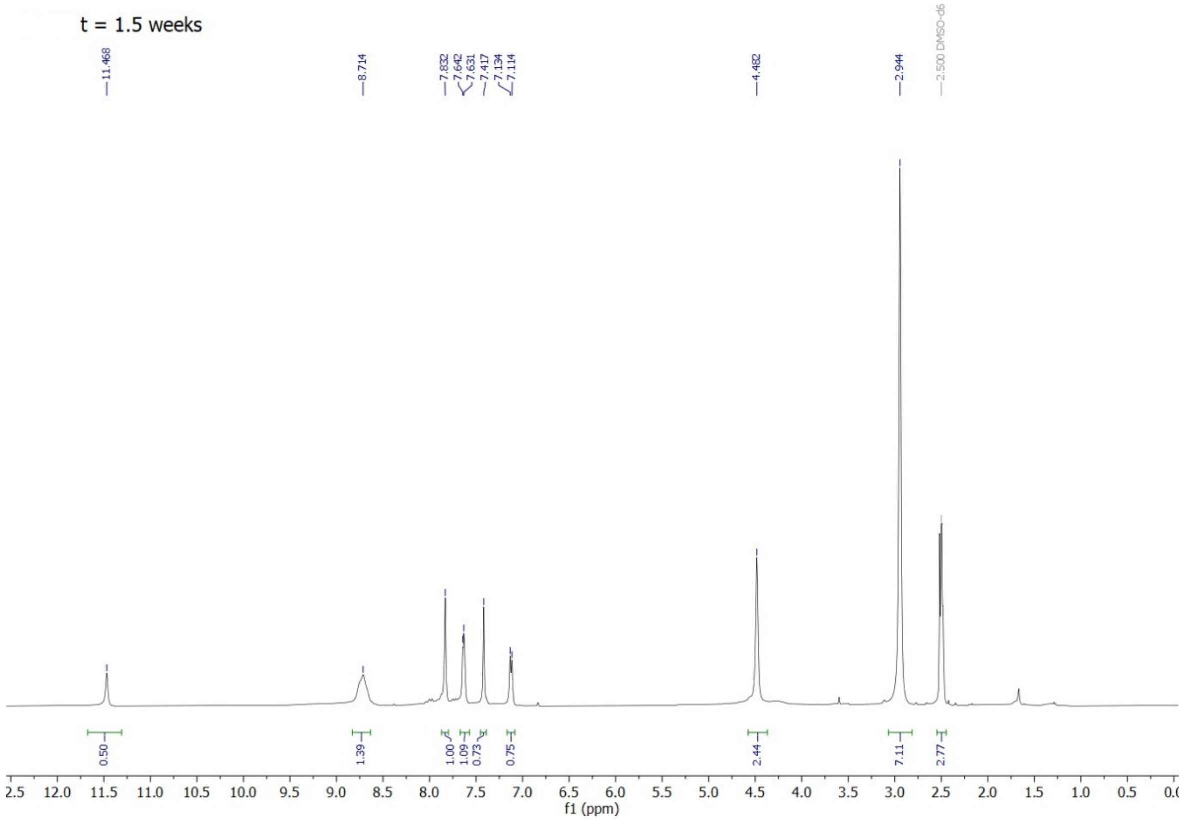

Supplementary Figure 17b: ^{1}H-NMR spectra of compound M-5HT (4) in DMSO-d_6 after 1.5 weeks.

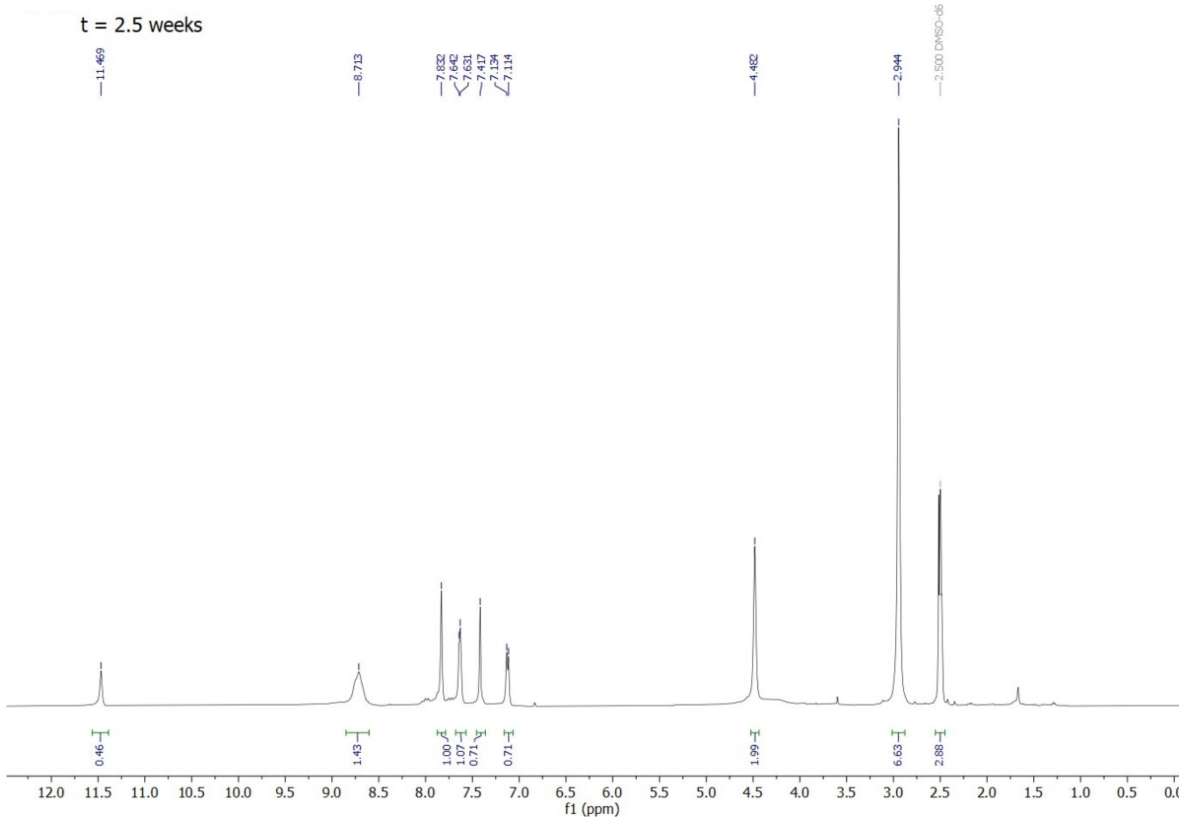

Supplementary Figure 17c: ^{1}H-NMR spectra of compound M-5HT (4) in DMSO-d_6 after 2.5 weeks.

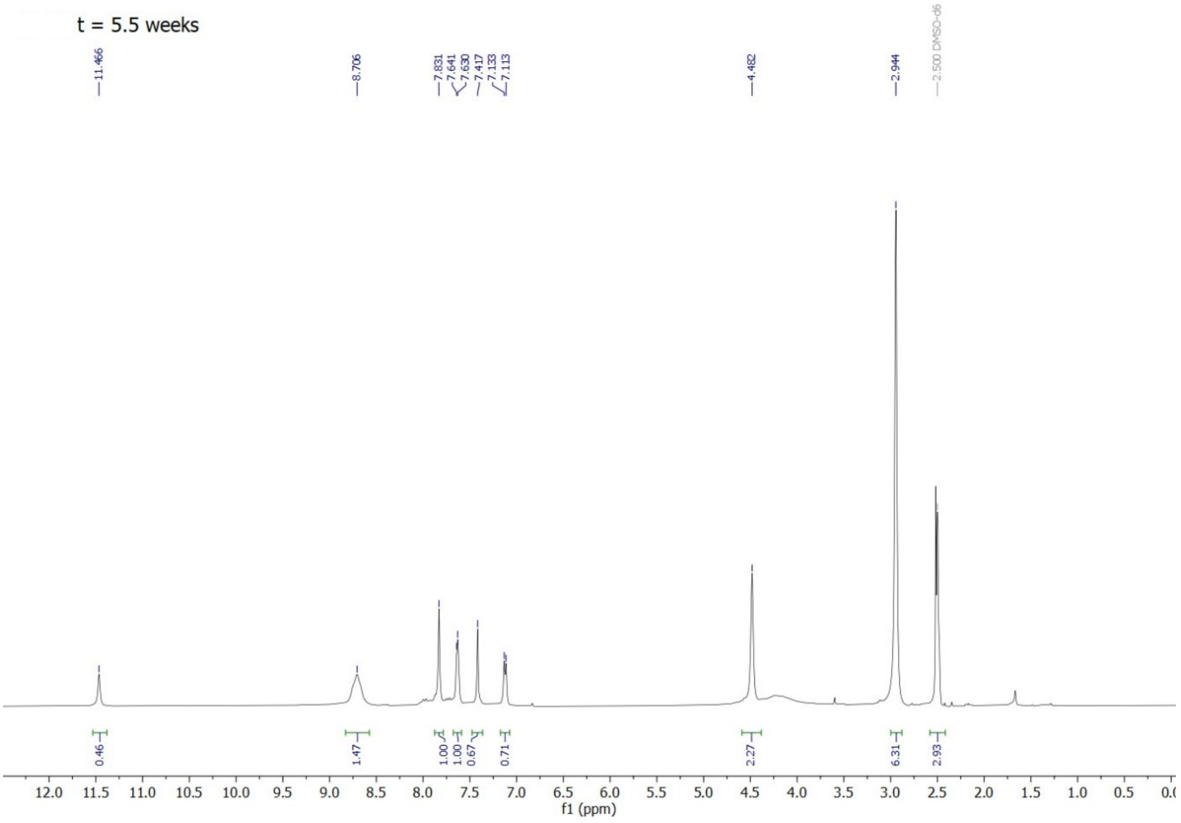

Supplementary Figure 17d: ¹H-NMR spectra of compound M-5HT (4) in DMSO-d_6 after 5.5 weeks.

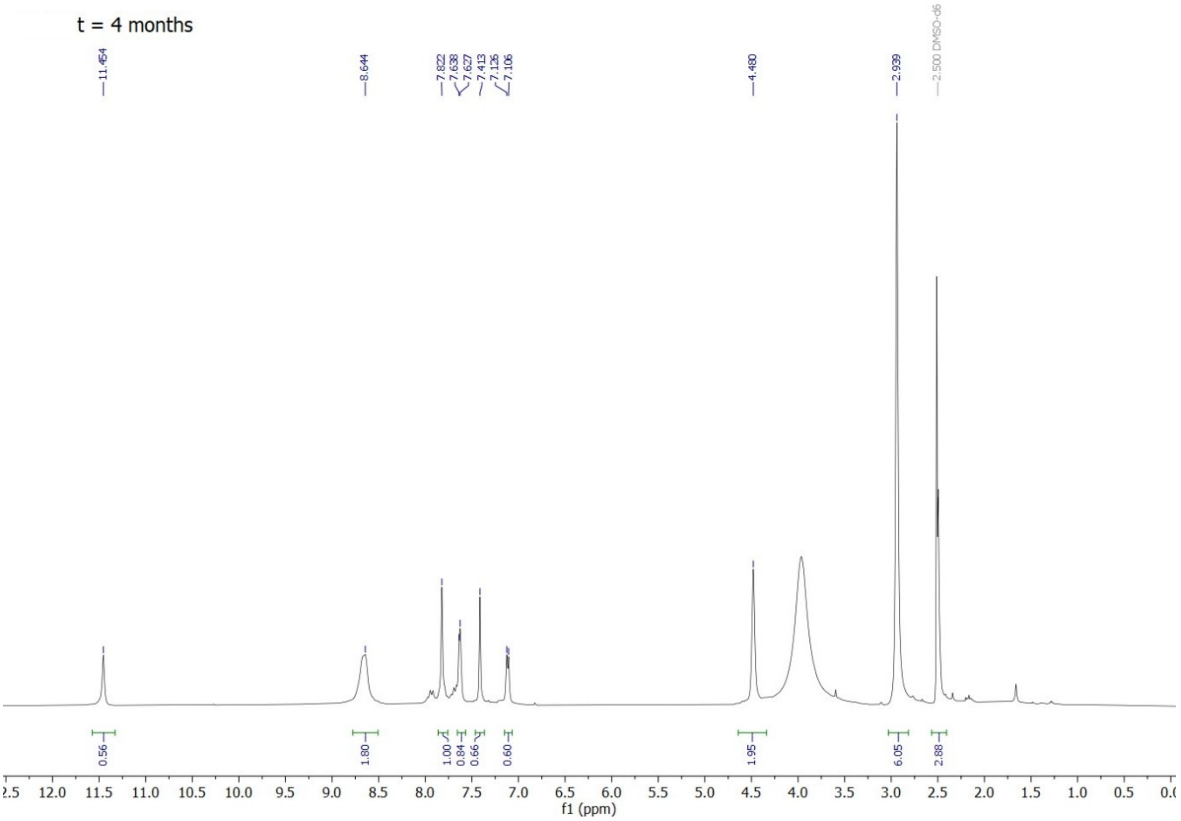

Supplementary Figure 17e: ¹H-NMR spectra of compound M-5HT (4) in DMSO-d_6 after 4 months.

3-(3-Aminopropyl)-1*H*-indol-5-ol (**7**)

A sample of compound P-5HT (**4**) in DMSO-d_6 was prepared and ^{1}H-NMRs measured immediately, after 2 weeks, 3 weeks, and 4 months. SI Fig. 18 shows the obtained stacked spectra for measurement in DMSO-d_6. As the stability studies were performed on preliminary material, the proton NMR shows some solvent and minor impurity peaks next to the compound peaks. Nevertheless, similar to the behaviour of compound M-5HT (**4**), no additional peaks occur during the observed time frame of 4 months. Deuterium exchange seems to be happening here too, as many peaks except the 7.09 ppm (H2) peak show slightly decreasing integrals and the ^{1}H-NMR after 4 months does suggest some precipitation. However, good stability of P-5HT (**7**) in DMSO-d_6 for at least three weeks was found **(SI Fig. 18a-d)**.

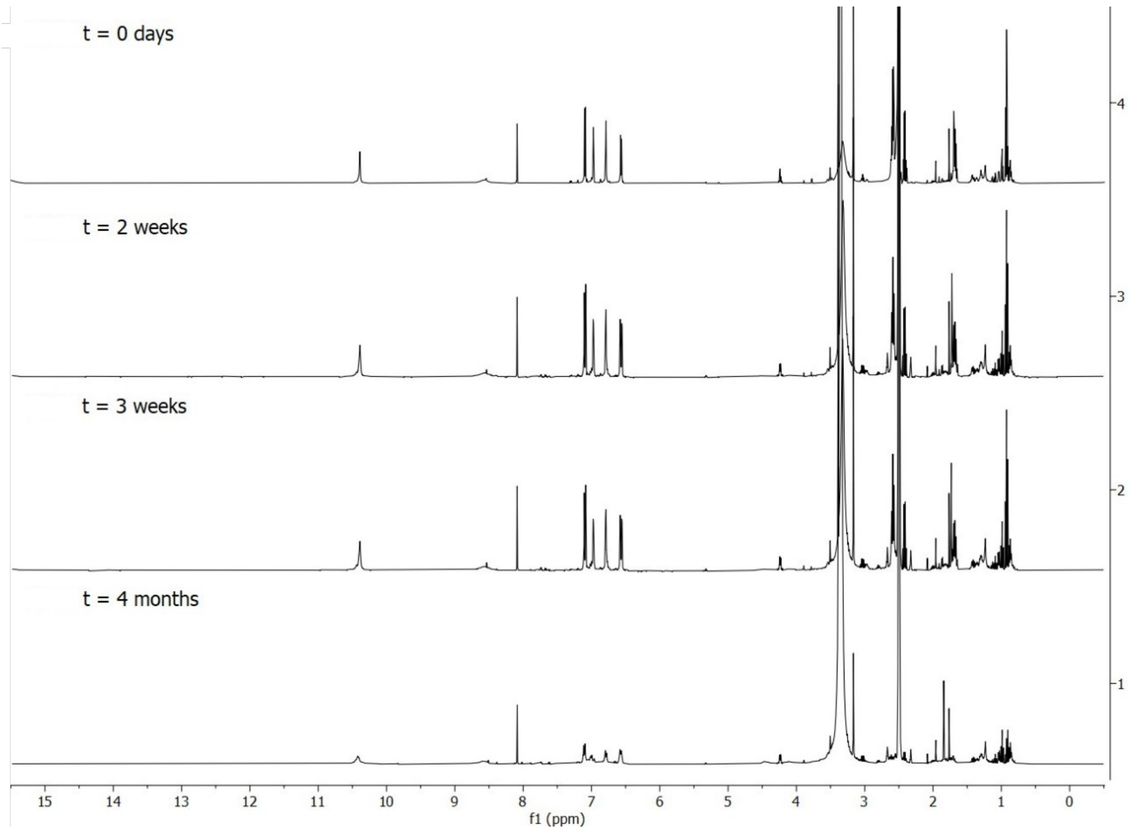

Supplementary Figure 18: Stacked ^{1}H-NMR spectra of compound P-5HT (7) measured after up to 4 months in DMSO-d_6. *The individual spectra are expanded in SI Fig. 18a-d.*

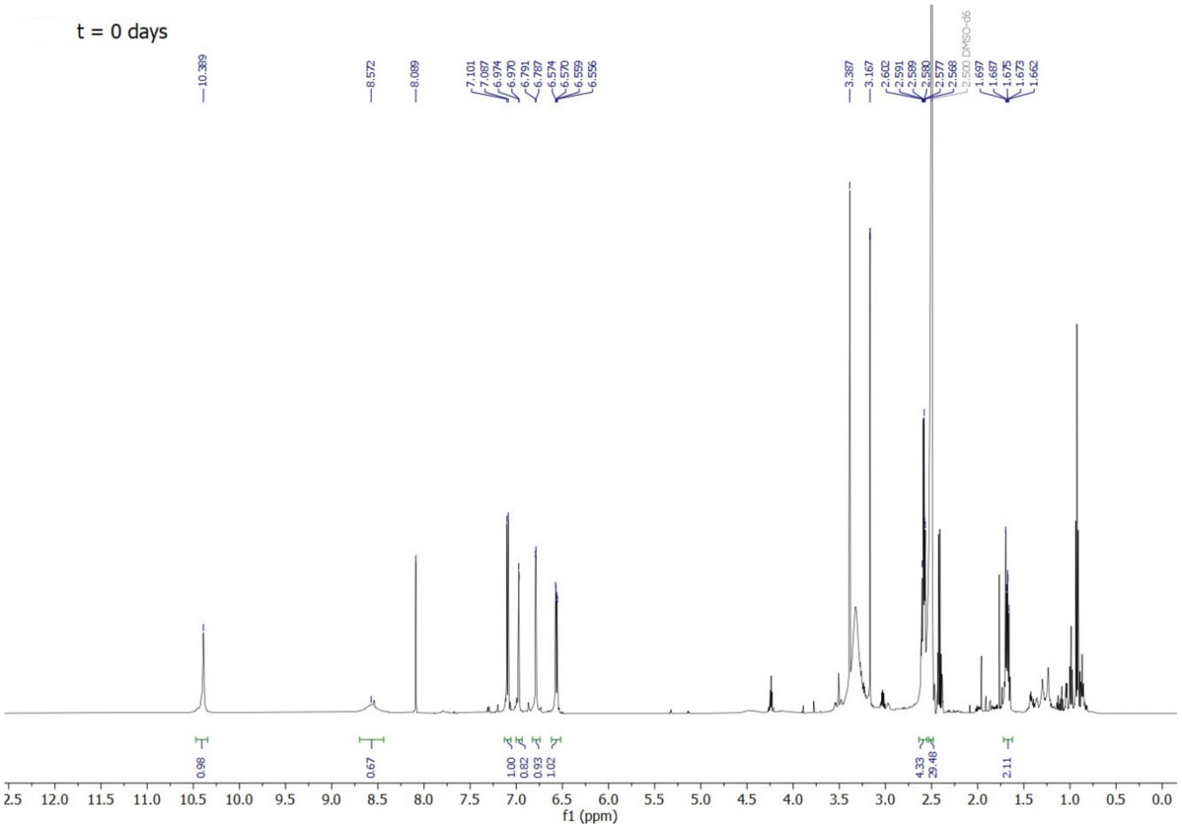

Supplementary Figure 18a: ^{1}H-NMR spectra of compound P-5HT (7) in DMSO-d_6 freshly prepared.

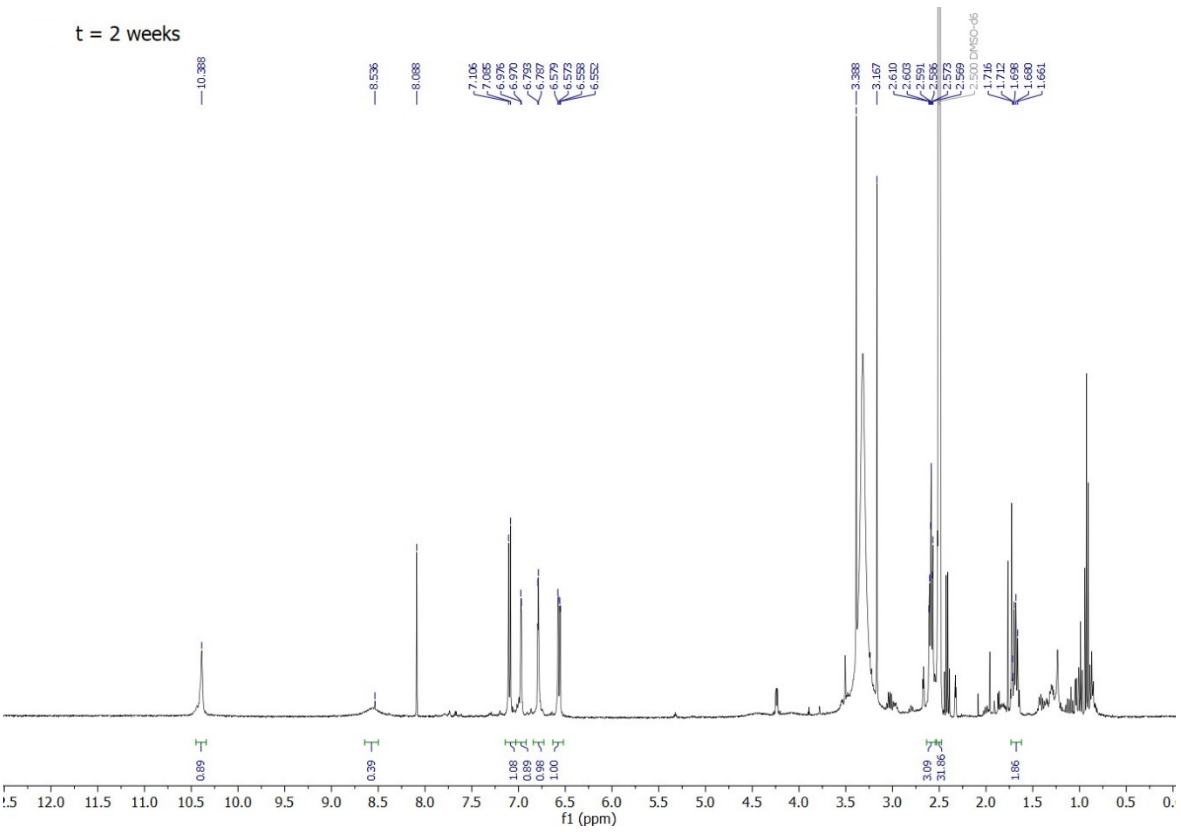

Supplementary Figure 18b: ^{1}H-NMR spectra of compound P-5HT (7) in DMSO-d_6 after 2 weeks.

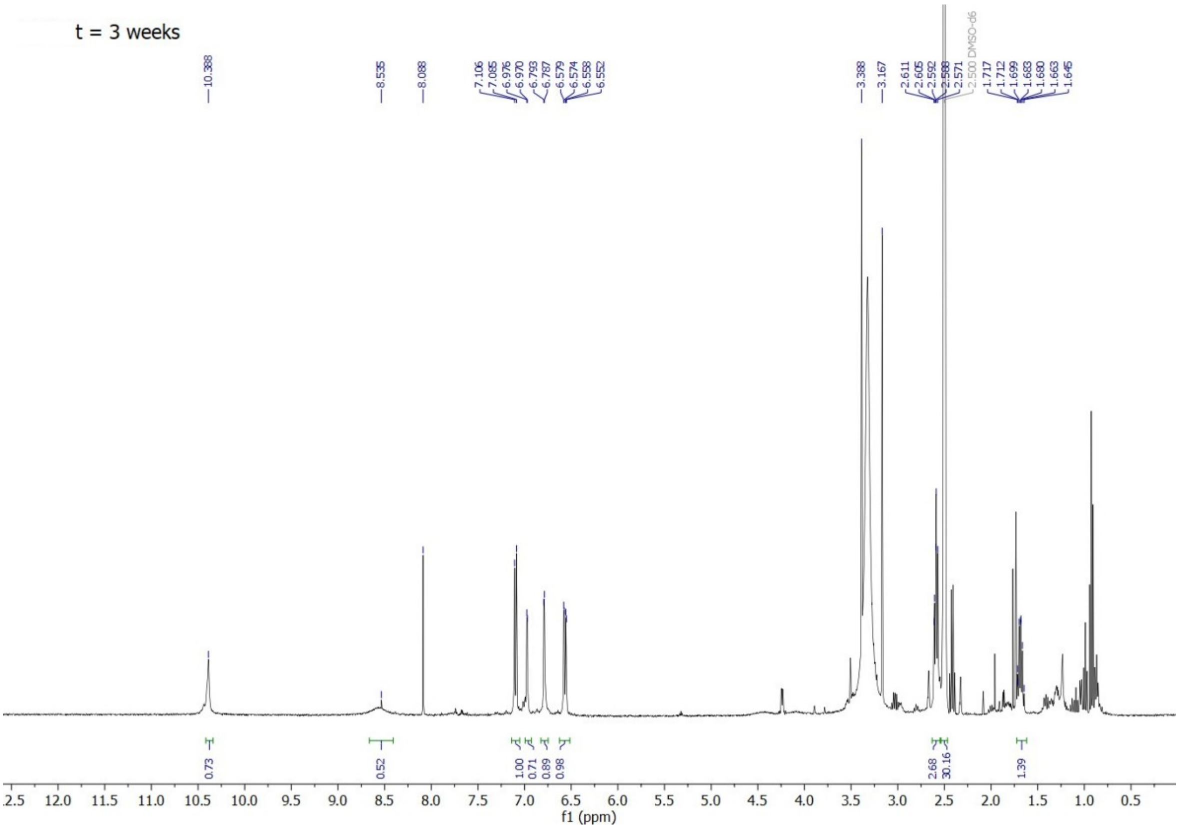

Supplementary Figure 18c: ^{1}H-NMR spectra of compound P-5HT (7) in DMSO-d_6 after 3 weeks.

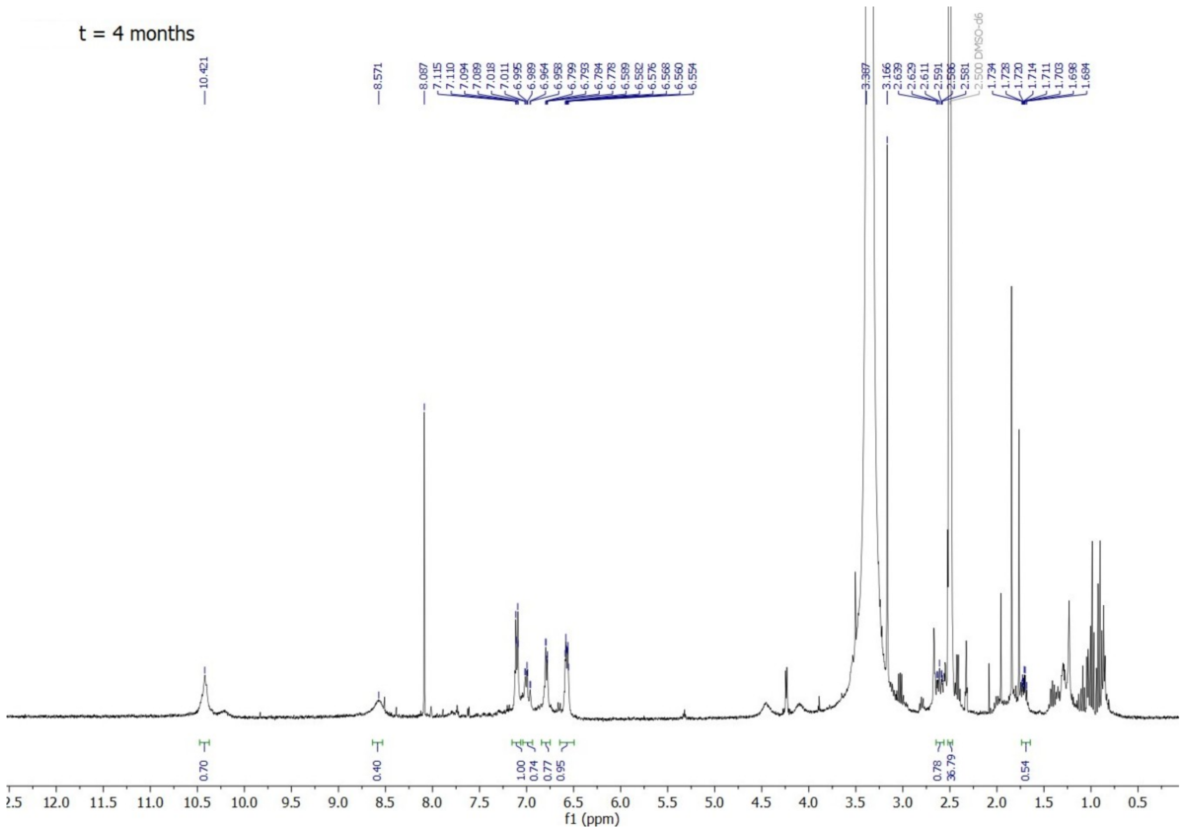

Supplementary Figure 18d: ^{1}H-NMR spectra of compound P-5HT (7) in DMSO-d_6 after 4 months.

3-(4-Aminobutyl)-1*H*-indol-5-ol (**10**)

A sample of compound B-5HT (**10**) in DMSO-d_6 was prepared and ^{1}H-NMRs measured immediately and after 1 month. Like compounds M-5HT (**4**) and P-5HT (**7**), no decomposition or significant change in the integrals could be observed. Thereby, we found that B-5HT (**10**) shows good stability in DMSO-d_6 for at least 1 month **(SI Fig. 19, 19a,b)**.

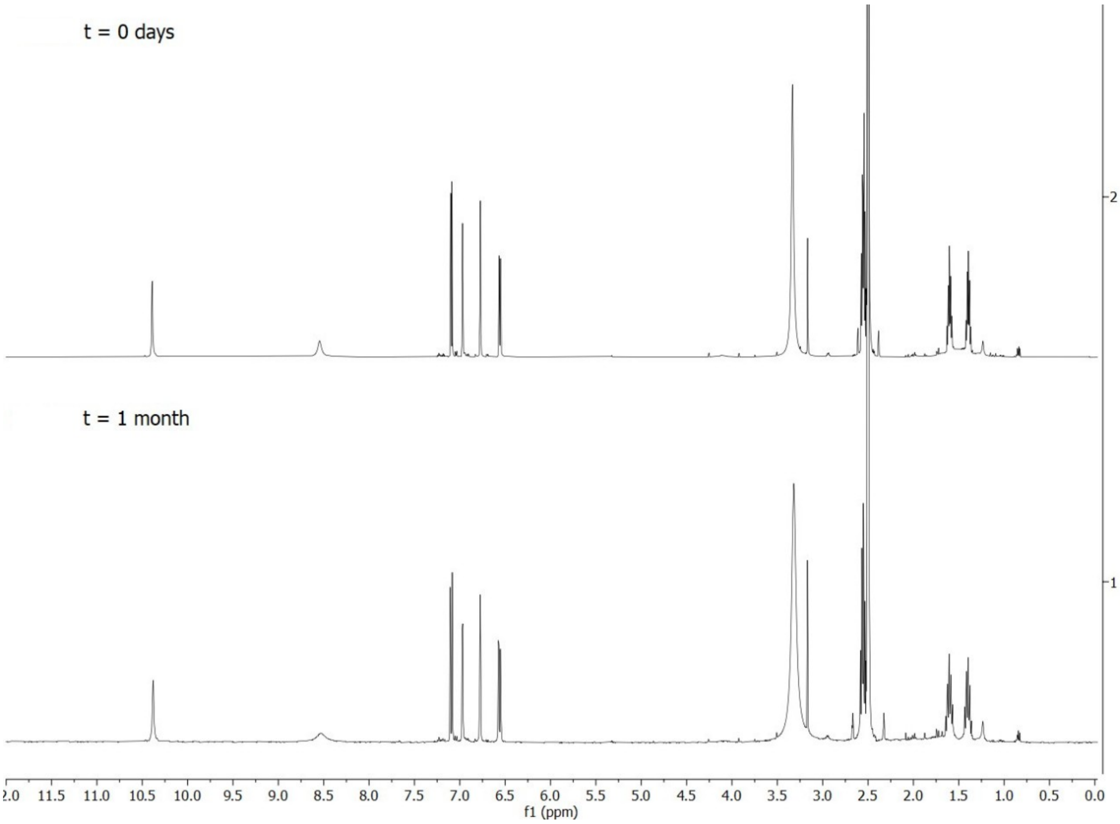

Supplementary Figure 19: Stacked ^{1}H-NMR spectra of compound B-5HT (10) measured immediately and after 1 month in DMSO-d_6. *The individual spectra are expanded in SI Fig. 19a,b.*

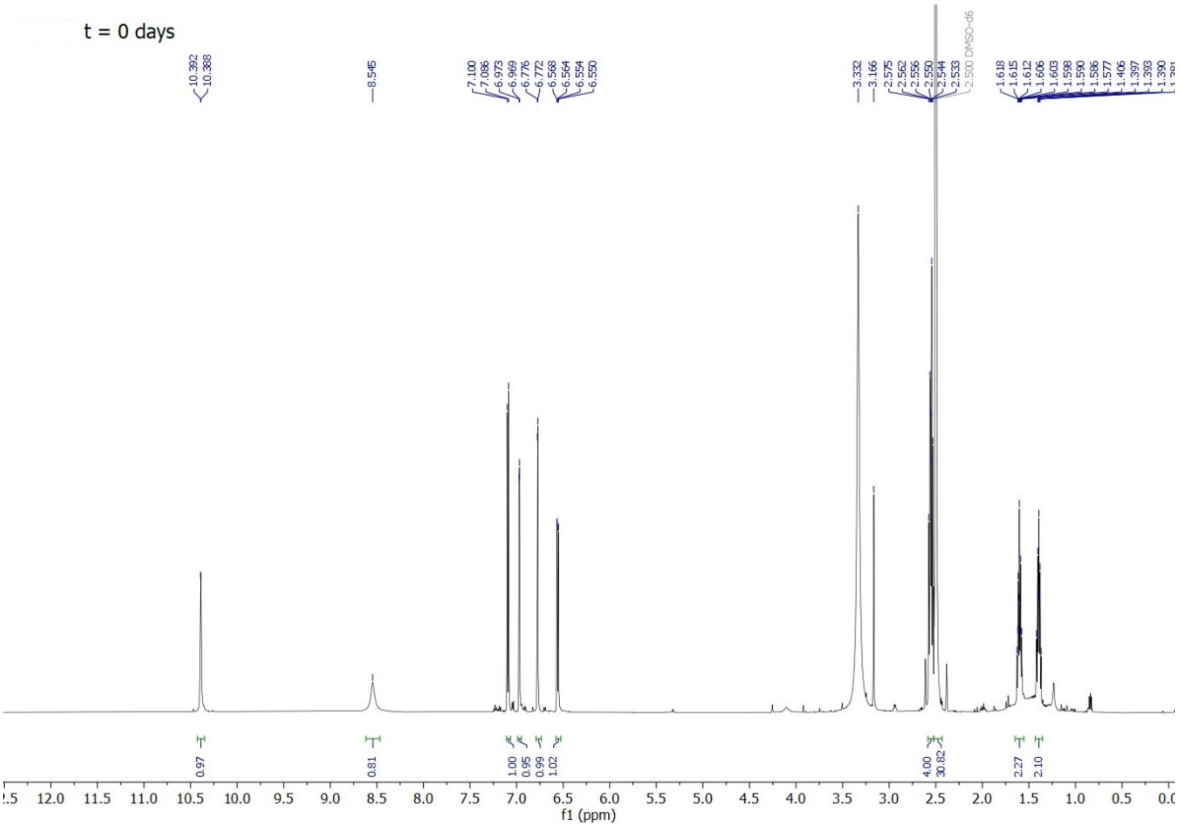

***Supplementary Figure 19a**: ^{1}H-NMR spectra of compound B-5HT (10) in DMSO-d_6 freshly prepared.*

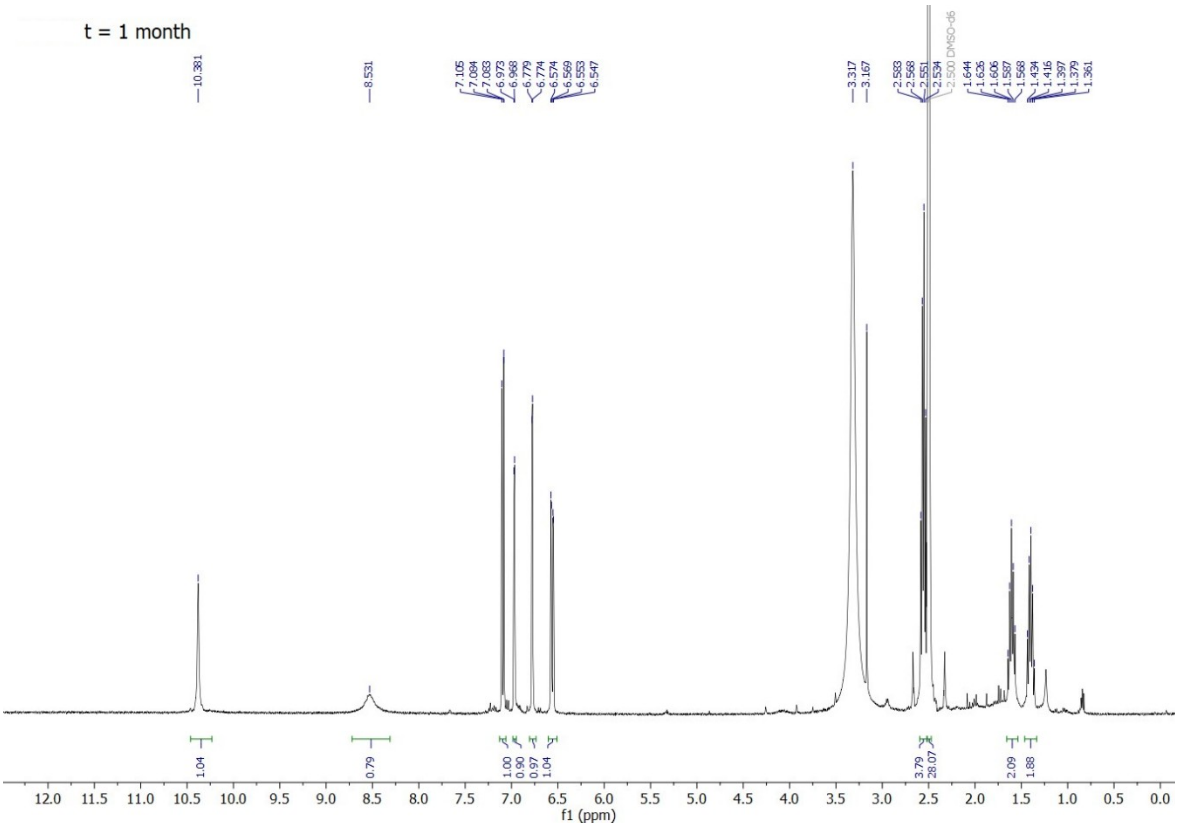

***Supplementary Figure 19b**: ^{1}H-NMR spectra of compound B-5HT (10) in DMSO-d_6 after 1 month.*

SUPPLEMENTARY REFERENCES

1. Schicker, K. *et al.* Unifying Concept of Serotonin Transporter-associated Currents. *J. Biol. Chem.* **287**, 438–445 (2012).

2. Niello, M. *et al.* Persistent binding at dopamine transporters determines sustained psychostimulant effects. *Proc. Natl. Acad. Sci.* **120**, (2023).

3. Bulling, S. *et al.* The Mechanistic Basis for Noncompetitive Ibogaine Inhibition of Serotonin and Dopamine Transporters. *J. Biol. Chem.* **287**, 18524–18534 (2012).

4. Pinder, R. M., Green, D. M., Brewster, K. & Thompson, P. B. J. The influence of chain length upon the activity of 5-hydroxytryptamine. *J. Pharm. Pharmacol.* **25**, 847–848 (2011).

5. Adkins, E. M., Barker, E. L. & Blakely, R. D. Interactions of Tryptamine Derivatives with Serotonin Transporter Species Variants Implicate Transmembrane Domain I in Substrate Recognition. *Mol. Pharmacol.* **59**, 514–523 (2001).

6. Karuvalam, R. P., Pakkath, R., Haridas, K. R., Rishikesan, R. & Kumari, N. S. Synthesis, characterization, and SAR studies of new (1H-indol-3-yl)alkyl-3-(1H-indol-3-yl)propanamide derivatives as possible antimicrobial and antitubercular agents. *Med. Chem. Res.* **22**, 4437–4454 (2013).

7. Katz, A. H. *et al.* Synthesis and analgesic activity of pemedolac [cis-1-ethyl-1,3,4,9-tetrahydro-4-(phenylmethyl)pyrano[3,4-b]indole-1-acetic acid]. *J. Med. Chem.* **31**, 1244–1250 (1988).

8. Stornaiuolo, M. *et al.* Structure-Based Lead Optimization and Biological Evaluation of BAX Direct Activators as Novel Potential Anticancer Agents. *J. Med. Chem.* **58**, 2135–2148 (2015).

9. Salonen, L. M. *et al.* Molecular Recognition at the Active Site of Factor Xa: Cation-π Interactions, Stacking on Planar Peptide Surfaces, and Replacement of Structural Water. *Chem. - A Eur. J.* **18**, 213–222 (2012).

10. Bender, C. F. & Widenhoefer, R. A. Room Temperature Hydroamination of N -Alkenyl Ureas Catalyzed by a Gold(I) N -Heterocyclic Carbene Complex. *Org. Lett.* **8**, 5303–5305 (2006).

11. Youn, S. W., Pastine, S. J. & Sames, D. Ru(III)-Catalyzed Cyclization of Arene-Alkene Substrates via Intramolecular Electrophilic Hydroarylation. *Org. Lett.* **6**, 581–584 (2004).
