# Supplementary material for: Ligand coupling mechanism of the human serotonin transporter differentiates substrates from inhibitors
Source: Nat Commun. 2024 Jan 10;15:417. doi: 10.1038/s41467-023-44637-6 (PMC10776687; doi:10.1038/s41467-023-44637-6)
Supplement: Supplementary file 3 — Reporting Summary [file 41467_2023_44637_MOESM3_ESM.pdf]

nature portfolio

Corresponding author(s): Thomas Stockner

Last updated by author(s): Dec 15, 2023

Reporting Summary

Nature Portfolio wishes to improve the reproducibility of the work that we publish. This form provides structure for consistency and transparency in reporting. For further information on Nature Portfolio policies, see our Editorial Policies and the Editorial Policy Checklist.

Statistics

For all statistical analyses, confirm that the following items are present in the figure legend, table legend, main text, or Methods section.

n/a	Confirmed	
☐	☒	The exact sample size (*n*) for each experimental group/condition, given as a discrete number and unit of measurement
☐	☒	A statement on whether measurements were taken from distinct samples or whether the same sample was measured repeatedly
☒	☐	The statistical test(s) used AND whether they are one- or two-sided<br>*Only common tests should be described solely by name; describe more complex techniques in the Methods section.*
☒	☐	A description of all covariates tested
☒	☐	A description of any assumptions or corrections, such as tests of normality and adjustment for multiple comparisons
☐	☒	A full description of the statistical parameters including central tendency (e.g. means) or other basic estimates (e.g. regression coefficient) AND variation (e.g. standard deviation) or associated estimates of uncertainty (e.g. confidence intervals)
☒	☐	For null hypothesis testing, the test statistic (e.g. *F*, *t*, *r*) with confidence intervals, effect sizes, degrees of freedom and *P* value noted<br>*Give P values as exact values whenever suitable.*
☒	☐	For Bayesian analysis, information on the choice of priors and Markov chain Monte Carlo settings
☒	☐	For hierarchical and complex designs, identification of the appropriate level for tests and full reporting of outcomes
☒	☐	Estimates of effect sizes (e.g. Cohen's *d*, Pearson's *r*), indicating how they were calculated

Our web collection on statistics for biologists contains articles on many of the points above.

Software and code

Policy information about availability of computer code

Data collection	Gromacs 2019.3<br>Clampex 11.0.3
Data analysis	R Studio 2022.07.0<br>Modeller 9.20<br>Pymol 2.5.2<br>MD Analysis 0.19.2<br>GraphPad Prism 9.5.1<br>Clampfit 10.7<br>Python 3.x<br>Force Distribution Analysis (FDA) 2.9.1<br>pyEMMA<br>Microsoft Office 365

For manuscripts utilizing custom algorithms or software that are central to the research but not yet described in published literature, software must be made available to editors and reviewers. We strongly encourage code deposition in a community repository (e.g. GitHub). See the Nature Portfolio guidelines for submitting code & software for further information.

Data

Policy information about availability of data

All manuscripts must include a data availability statement. This statement should provide the following information, where applicable:

- Accession codes, unique identifiers, or web links for publicly available datasets
- A description of any restrictions on data availability
- For clinical datasets or third party data, please ensure that the statement adheres to our policy

All data, including the source data of the figures as well as each start end structure of SERT, extracted from all trajectories, are available at Zenodo (https://zenodo.org) with the DOI: 10.5281/zenodo.10381879. The PDB entry (5I71) for the crystal structure of SERT in the outward-open state with a resolution of 3.15 A [https://www.rcsb.org/structure/5I71] was used as a starting point to generate MD input structures. The PDB entries for SERT in the outward-open (5I71) [https://www.rcsb.org/structure/5I71], the occluded (6DZV) [https://www.rcsb.org/structure/6DZV], and inward-open (6DZZ) [https://www.rcsb.org/structure/6DZZ] were used to generate reference distances across the outer vestibule. The X-ray structure of Drosophila dopamine transporter in complex with S1-bound cocaine (4XP4) [https://www.rcsb.org/structure/4XP4] was used as a template to manually dock cocaine into outward-open ion-bound SERT.

Research involving human participants, their data, or biological material

Policy information about studies with human participants or human data. See also policy information about sex, gender (identity/presentation), and sexual orientation and race, ethnicity and racism.

Reporting on sex and gender	The study did not include human participants
Reporting on race, ethnicity, or other socially relevant groupings	The study did not include human participants
Population characteristics	The study did not include human participants
Recruitment	The study did not include human participants
Ethics oversight	The study did not include human participants

Note that full information on the approval of the study protocol must also be provided in the manuscript.

Field-specific reporting

Please select the one below that is the best fit for your research. If you are not sure, read the appropriate sections before making your selection.

☒ Life sciences ☐ Behavioural & social sciences ☐ Ecological, evolutionary & environmental sciences

For a reference copy of the document with all sections, see nature.com/documents/nr-reporting-summary-flat.pdf

Life sciences study design

All studies must disclose on these points even when the disclosure is negative.

Sample size	No sample size calculations were performed for this study. For in vitro assays, a minimum of n=3 biologically independent experiments measured in triplicates, were conducted. For electrophysiological measurements, a minimum of n=5 independent cells per ligand were recorded. The sample size for ex vivo experiments was modeled based on previously published work, demonstrating fenfluramine-mediated [3H]5HT release in hippocampal brain tissue (DOI 10.1021/acschemneuro.8b00689). In silico sample size: n=10 independent trajectories for each condition (apo, M-5HT, 5HT, P-5HT, B-5HT, and cocaine). All sample sizes are described in detail in every single figure legend.
Data exclusions	Rarely extreme outliers in in vitro assays were cautiously excluded, otherwise data was kept as complete as possible
Replication	Experiments were at least conducted in three (n=3) individual, biologically independent assays, in triplicate if appropriate. Rarely attempts at replication were unsuccessful and in such cases experiments were repeated two more times before excluding any gathered data. For MD simulations, 10 replicas per conditions were conducted. For ex vivo studies, replications were performed with the number of individual mice used in each experiment.
Randomization	No randomization was performed in this study. The compounds (M-5HT, P-5HT, and B-5HT) displayed different colours when dissolved in solvent. In MD simulations, all molecules are always explicitly present.
Blinding	Functional in vitro and ex vivo studies do not require blinding, as the data obtained from these experiments is objective and does not involve any subjective assessment by the experimenter. Similarly, analysis of MD data is objective.

Reporting for specific materials, systems and methods

We require information from authors about some types of materials, experimental systems and methods used in many studies. Here, indicate whether each material, system or method listed is relevant to your study. If you are not sure if a list item applies to your research, read the appropriate section before selecting a response.

Materials & experimental systems

n/a	Involved in the study	
x		Antibodies
	x	Eukaryotic cell lines
x		Palaeontology and archaeology
	x	Animals and other organisms
x		Clinical data
x		Dual use research of concern
x		Plants

Methods

n/a	Involved in the study	
x		ChIP-seq
	x	Flow cytometry
x		MRI-based neuroimaging

Eukaryotic cell lines

Policy information about cell lines and Sex and Gender in Research

Cell line source(s)	ATCC, human kidney cells 293
Authentication	Authenticated by STR profiling at the Medical University of Graz (Cell Culture Core Facility)
Mycoplasma contamination	Mycoplasma contamination was regularly tested and could be ruled out for all cell lines used in the study
Commonly misidentified lines (See ICLAC register)	None

Animals and other research organisms

Policy information about studies involving animals; ARRIVE guidelines recommended for reporting animal research, and Sex and Gender in Research

Laboratory animals	Adult male WT C57Bl6/N mice , 12 weeks old
Wild animals	Study did not involve wild animals
Reporting on sex	Only male mice were included
Field-collected samples	Study did not involve samples collected from the field
Ethics oversight	All animals experiments were conducted in agreement with the ARRIVE guidelines and the UK Animals (Scientific Procedures Act, 1986 and associated guidelines, EU Directive 2010/63/EU for animal experiments) and approved by the national ethical committee on animal care and use (Bundesministerium für Bildung, Wissenschaft und Forschung: BMBWF-2022-0.121.471).

Note that full information on the approval of the study protocol must also be provided in the manuscript.

Plants

Seed stocks	None
Novel plant genotypes	None
Authentication	None

Flow Cytometry

Plots

Confirm that:

- [x] The axis labels state the marker and fluorochrome used (e.g. CD4-FITC).
- [x] The axis scales are clearly visible. Include numbers along axes only for bottom left plot of group (a 'group' is an analysis of identical markers).
- [x] All plots are contour plots with outliers or pseudocolor plots.
- [x] A numerical value for number of cells or percentage (with statistics) is provided.

Methodology

Sample preparation	HEK293 cells stably expressing YhSERT WT were dissociated by incubation in 1X Trypsin-EDTA (0.5 g x L-1) for 5 minutes, resuspended in FBS-free DMEM, and the usage of a 40 µm cell strainer (Cornig Nr. 352235). See Methods section.
Instrument	BD FACSAria Fusion Flow Cytometer (BD Biosciences). See Methods section.
Software	BD FACSDiva version 8.0.2 and FlowJo version (TreeStar) 10.10.0 software. See Methods section.
Cell population abundance	Details are described in the Methods section and population abundances are depicted in the Supplementary Information (SI Fig. 5)
Gating strategy	Population 1 (P1) was defined by a forward scatter (FSC)-area (A) and a side scatter (SSC)-A to facilitate the removal of debris. Population 2 (P2) was defined by FSC-A and FSC-height (H) to identify cells of interest characterised by size and granularity, respectively. The final population, population 3 (P3), was based on N-terminally fused eYFP fluorescence intensity, defined by fluorescein isothiocyanate (FITC)-A (488 nm, 530/30 emission filter) and allophycocyanin (APC)-A (640 nm, 670/30 emission filter). See Methods section.

- [x] Tick this box to confirm that a figure exemplifying the gating strategy is provided in the Supplementary Information.
